# Fostering Adherence to Evidence-Based Care in the Management of Musculoskeletal Shoulder Pain: A Mixed-Methods Study

**DOI:** 10.1093/ptj/pzae176

**Published:** 2024-12-16

**Authors:** Christina Maxwell, Jon Salsberg, Katie Robinson, François Desmeules, Véronique Lowry, Christie Tetreault, Karen McCreesh

**Affiliations:** School of Allied Health, Health Research Institute, University of Limerick, Limerick, Ireland; Ageing Research Centre, Health Research Institute, University of Limerick, Limerick, Ireland; Ageing Research Centre, Health Research Institute, University of Limerick, Limerick, Ireland; School of Medicine, Health Research Institute, University of Limerick, Castletroy, Limerick, Ireland; School of Allied Health, Health Research Institute, University of Limerick, Limerick, Ireland; Ageing Research Centre, Health Research Institute, University of Limerick, Limerick, Ireland; School of Rehabilitation, University of Montreal, Montreal, Quebec, Canada; Faculty of Medicine and Health Sciences, Université de Sherbrooke, Sherbrooke, Quebec, Canada; School of Psychology, University of Saskatchewan, Saskatoon, Saskatchewan, Canada; School of Allied Health, Health Research Institute, University of Limerick, Limerick, Ireland; Ageing Research Centre, Health Research Institute, University of Limerick, Limerick, Ireland

**Keywords:** Concept Mapping, Educational Priorities, Knowledge Translation, Mixed Methods, Public and Patient Involvement (PPI), Shoulder Pain

## Abstract

**Objective:**

The objective was to identify the priorities of individuals with musculoskeletal shoulder pain and their health care providers (HCPs) that are perceived to foster multistakeholder adherence to evidence-based recommendations.

**Methods:**

The study used a mixed-methods design, informed by concept mapping. Patients with shoulder pain (ie, ≥6 weeks) and HCPs involved in their care (recruited via social media, email, etc) were invited to complete an initial survey to identify perceived priorities to foster adherence to evidence-based recommendations. Preliminary data sorting resulted in a final priority list, with a subset (*n* = 20) of respondents rating their importance using a Likert scale. A public and patient involvement (PPI) panel (*N* = 8) assisted in this rating phase, further sorting of priorities based on thematic similarities (ie, into categories and then domains), analysis, interpretation, and developing a concept map illustrating relationships between them.

**Results:**

One hundred and fifty-four participants (HCPs = 133; patients = 21) responded to the initial survey, generating 77 priorities, grouped into 13 categories, and then into 3 domains: (1) Education, (2) Patient-centered care, and (3) Health care communication. Patients prioritized categories relating to the provision of a specific diagnosis, the establishment of a strong therapeutic relationship, and the need for education on progress and recovery timelines, as well as treatment options. HCPs prioritized these same educational categories, also prioritizing the need for tailoring exercise therapy and providing a unified message on best management. PPI panelists identified education on treatment options coupled with a strong therapeutic alliance and a unified message on best management to be of pivotal importance in fostering adherence. Panelists also stressed that future knowledge translation resources must provide tailored education.

**Conclusion:**

HCPs and patients agree on the need to prioritize education related to progress and recovery timelines as well as treatment options, with a strong therapeutic alliance and a unified message on best management also considered of pivotal importance for adherence to evidence-based recommendations.

**Impact:**

To the knowledge of the authors, this is the first study, including a broad range of stakeholder groups spanning across 11 different countries, to explore the priorities that stakeholders perceived to support stricter adherence to evidence-based recommendations for musculoskeletal shoulder pain, with the relationship between these priorities visually illustrated using a concept map. Patients and HCPs were united in their prioritization of education relating to expected progress and recovery timelines, as well as treatment options and supporting evidence. Stakeholders also identified the need for greater emphasis to be placed on establishing a therapeutic relationship and on integrating shared decision-making into clinical practice to further facilitate adherence. Education relating to treatment options and supporting evidence, a strong therapeutic relationship, and a unified message on best management were perceived by the PPI panel as being pivotal in facilitating adherence to evidence-based treatment. The findings of this study highlight the need for improved tailoring of educational resources for shoulder pain, as well as more cohesive messaging from health care providers, both assisting in supporting first-line treatments, such as exercise therapy, for musculoskeletal shoulder pain.

## INTRODUCTION

Shoulder pain is the third most common musculoskeletal (MSK) condition presenting in primary care, with over half reporting persistent pain beyond 6 months.^1^ Health care providers (HCPs) have an obligation to ensure that treatment is both informed by and reflective of research evidence.^2^ Despite this, many inconsistencies exist in the implementation of treatment recommendations for this population,^3–5^ most notably relating to rising surgical rates,^5^ in contrast to strong recommendations against this approach for most common atraumatic shoulder pathologies.^6^^,^^7^

Stakeholders have expressed numerous challenges to implementing evidence-based care recommendations.^8^^,^^9^ HCPs have expressed difficulties in getting patients to “buy-in” to first-line treatment, such as exercise therapy,^8^ with patients’ mistrust in their physical therapist flagged as one potential barrier.^10^ Other potential explanations become apparent when exploring patients’ perspectives, with many expressing apprehensiveness toward exercise, founded in beliefs that their pain is due to structural damage,^8^ a serious pathology,^11^ and/or associating surgery with a more permanent “fix”.^8^ Similar challenges have been echoed by stakeholders in relation to treatment decision-making, with both patients and HCPs expressing a deeply rooted biomechanical view of pain, subsequently influencing the education provided to and sought by patients.^12^

Based on these findings, it is unsurprising that HCPs are questioning the effectiveness of their educational strategies,^13^ with poor education shown to result in feelings of uncertainty among patients.^11^ Identifying such evidence–practice gaps is crucial to understand the rationale for treatment decisions incongruent with best-evidence recommendations. As is the need to identify what factors may be influencing stakeholder motivation to adhere with these recommendations, reflecting on the 3 innate psychological needs postulated to enhance intrinsic motivation—competence, autonomy, and relatedness.^14^ Such findings may help to inform the development of future knowledge translation (KT) strategies supporting the implementation of evidence-based recommendations into clinical practice.^15^ Hence, the aim of this study was to identify the priorities of individuals with musculoskeletal shoulder pain and their HCPs that are perceived to foster multistakeholder adherence to evidence-based treatment recommendations.

## METHODS

### Methodology

**Figure 1:**
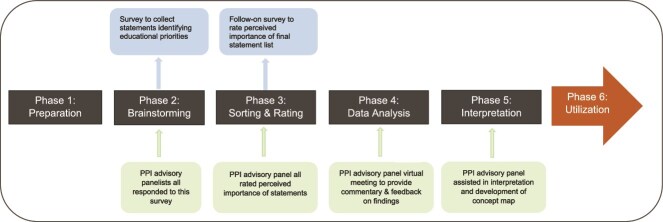
Overview of Concept Mapping Approach Illustrating PPI During Phases 2 to 5, With Surveys Conducted as Part of Phases 2 and 3. PPI = public and patient involvement.

This study adopted a mixed-methods approach informed by concept mapping methodology. Mixed-methods research combines elements of qualitative and quantitative research approaches, helping to provide a fuller picture that can enhance description and understanding of phenomena.^16^ This approach was adopted to help provide a more holistic picture of stakeholders’ priorities relating to adherence with evidence-based recommendations, with the inclusion of a more qualitative approach in the analysis helping to capture unanticipated facets of the topic and also help in the interpretation of quantitative data.^17^ Concept mapping aims to organize and represent ideas from an identified group,^18^ and visually represent these ideas (or categories) using a concept map,^19^ to assist in communicating findings and strengthening conceptual analysis.^20^ It involves 6 phases, including a preparatory phase (1) involving topic familiarization and preparation of the initial survey, with subsequent phases illustrated in Figure 1.^21^ Although the utilization of phase (6), involving stakeholders jointly determining the best ways to use the concept map produced,^22^ is not addressed as part of this study, it is the intention that the findings will inform the development of future KT resources. The flexibility afforded by concept mapping enables it to be tailored to the needs of the project.^23^ Therefore, this study adopted a predominantly qualitative approach to analysis and concept map development, as conducted in previous research,^20^ facilitating the inclusion of a public and patient involvement (PPI) panel to collaboratively analyze the data.^24^ This multistakeholder PPI panel (*N* = 8) assisted in study Phases 2 to 5 (see Figure 1), enhancing the value, integrity, and quality of the research.^25^ This study was approved by the Bon Secours Health System (6/16/22) and the Galway University Hospitals Clinical Research Ethics Committees (Ref: C.A. 2251, 6/22/22).

### Eligibility

Patients with a history (≥6 weeks) of shoulder pain (eg, rotator-cuff related, subacromial impingement, instability, or frozen shoulder) and any HCPs (eg, general practitioners, physical therapists, or orthopedic surgeons) currently involved in their care were considered eligible. All participants provided an electronic signature to self-confirm eligibility and provide consent.

### Sampling and Recruitment

**Figure 2:**
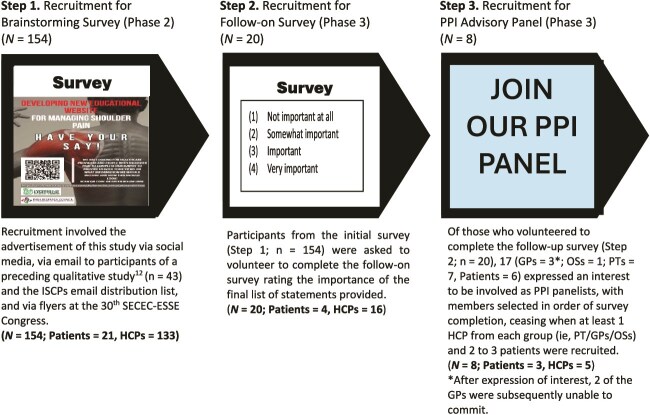
Survey Participants and PPI Advisory Panel Recruitment Steps. GP = general practitioner; HCPs = health care providers; ISCPs = Irish Society of Chartered Physiotherapists; OSs = orthopedic surgeons; PPI = public and patient involvement; PTs = physical therapists; SECEC-ESSE = Société Européenne pour la Chirurgie de l’Epaule et du Coude–European Society for Surgery of the Shoulder and the Elbow.

Using purposeful sampling, participants were recruited that met the defined characteristics outlined in the above eligibility criteria, with such stakeholders considered most likely to yield appropriate and useful information to meet the objective of this study.^26^ Purposeful sampling is considered best practice within qualitative research,^27^ with the provision of clearly defined sampling criteria assisting to enhance the rigor and trustworthiness of these research findings.^28^ Recruitment for the 2 surveys (Phases 2 and 3) and for the PPI panel took place between August 2022 and October 2022, as illustrated in Figure 2. Multiple advertisement strategies were used: emailing participants of a preceding qualitative study,^12^ via social media (Twitter, LinkedIn, and Facebook), the Irish Society of Chartered Physiotherapists email distribution list, hand-held flyers at the 30th Société Européenne pour la Chirurgie de l'Epaule et du Coude–European Society for Surgery of the Shoulder and the Elbow (SECEC-ESSE) Congress, as well as the research team’s personal and professional networks. Advertisement material contained an anonymous survey link. Survey respondents (Phase 2) were asked to provide their contact details if they were interested in volunteering to complete a follow-on survey (Phase 3) and/or as PPI panelists. PPI panel recruitment ceased when at least 1 HCP from each group (ie, Physical Therapist/General Practitioners/Orthopedic Surgeons) and 2 to 3 patients were recruited. Panelists received a €100 compensation voucher.

### Preparation (Phase 1)

Five of the authors (C.M., K.R., K.M., J.S., and F.D.) took part in the preparation of this study, including its aim, schedule, and logistics.

### Brainstorming Survey (Phase 2)

Surveys were conducted using Qualtrics software (Qualtrics, Provo, UT, USA). Basic demographic information was collected: age, sex, country of residence, and occupation. Additional information was sought from HCPs relating to their work setting, years of experience, and postgraduate training. Patients were also asked to provide details relating to duration of pain, diagnosis, and treatment. Respondents were asked to complete a statement in response to 1 of the following focus prompts: for HCPs, “*Adherence to evidence-based treatment would be more likely if…*” and for patients, “*It would be easier for me to follow treatment advice if…*” with the option to submit up to 5 statements. Data collection continued until the research team determined saturation had occurred (ie, no new priorities emerged).^29^

### Sorting and Rating (Phase 3)

The preliminary sorting of phase 2 survey responses was conducted separately for patients (Suppl. Material 1) and HCPs (Suppl. Material 2). Data sorting involved editing of raw statements (Round 1) (C.M.) and then condensing of these based on thematic similarities (Round 2) (C.M., V.L., K.M., K.R.) to create a final list. HCPs and patients who had volunteered from the initial survey, including those from the PPI panel, then completed the follow-on Phase 3 survey rating the relative importance of these statements using a 4-point Likert scale (1 = not important to 4 = very important). Similar synthesis-reduction processes have been employed in concept mapping studies relating to knee arthritis and cognitive health.^30–32^

Prior to Phase 4, final statements were further condensed, sorted, and grouped into preliminary categories (Suppl. Material 3) (C.M., V.L.). This involved minor editing to shorten statements, renamed as “priorities” (eg, patient final statement 3 “*I had a clear pathway of care*” shortened to “*clear care pathway*”). To ensure a central focus on the patient perspective, preliminary categories were created by the grouping of patient priorities based on thematic similarities, with category titles chosen to best represent these grouped priorities (eg, Category 4 “*A specific diagnosis*” including the priority “*Clear cause of pain*”), with all HCP priorities subsequently mapped onto these patient categories (C.M., V.L.).

### Analysis and Interpretation (Phases 4 and 5)

**Figure 3:**
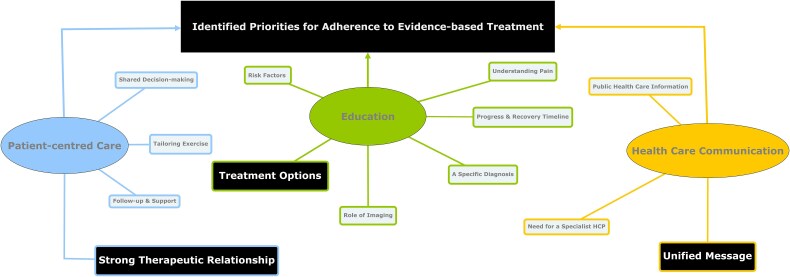
Concept Map Illustrating Categories Prioritized Among Stakeholders as Important for Adherence to Evidence-Based Treatment Recommendations, With the 3 Categories Perceived by the Public and Patient Involvement (PPI) Panel to be of Key Importance Highlighted in Black (ie, Strong Therapeutic Relationship, Unified Message, Treatment options). HCP = health care provider.

Descriptive statistics were run for the perceived level of importance of each priority (C.M.), as identified in Phase 3, with chi-square test analyses conducted to determine if there were statistically significant differences between the proportion of patients and HCPs who perceived categories as a priority (or not) (C.T.). A consultative collaborative analysis approach was then adopted, involving the researchers conducting the data analysis and then subsequently presenting preliminary finding to the PPI panel for commentary and feedback.^24^ This collaborative approach to analysis has been shown to enhance the thoroughness of interpretations drawn,^24^ facilitate a more in-depth understanding,^33^ as well as to improve the accessibility and quality of the research.^34^ Using Microsoft Teams (V.1.5.00.27363; Microsoft Corporation, Redmond, WA, USA) and Google Jamboard (https://jamboard.google.com/; Google, Mountain View, CA, USA), a virtual meeting was held with the PPI panel to present the preliminary categories for commentary and feedback (Suppl. Material 3), facilitated by 4 of the authors (C.M., J.S., V.L., and K.R.). Verbal consent was obtained for audio-recording and transcription. Two panelists were unable to attend (O.S. and G.P.); however, they provided detailed feedback via email. The meeting notes, audio, and transcript were reviewed by C.M., with amendments reflecting panel interpretations and recommendations. The panel predominantly focused on categories where there was less consensus between patients and HCPs, identified by a statistically significant difference and/or where there were greater percentage differences in contribution to the category (ie, ≥10%). The latter was calculated based on the number of initial survey respondents who contributed statements to the priorities within each category by each stakeholder group, divided by the total number within the stakeholder group. Panelists were also asked to consider the rated importance and the focus of these priorities when interpreting these data. Subsequently, a member-checking stage took place with PPI panelists where they were emailed a detailed discussion summary and a draft concept map (Suppl. Material 4), adding an additional layer of interpretative value.^35^ Panelists provided minor suggestions to amend the grouping of priorities, to further condense/edit to enhance clarity, and also assisted in the grouping of categories into domains based on thematic similarities. The final concept map illustrated these identified domains, categories, and relationships between categories (Figure 3).

### Role of the Funding Source

The Irish Research Council, who provided funding for this review, had no involvement in the study design; in the collection, analysis, and interpretation of data; in the writing of the report; or in the decision to submit this article for publication.

## RESULTS

A summary of participant demographics, HCP occupational characteristics, and patient shoulder pain characteristics, including the number who engaged in each study phase, is presented in Tables 1 and 2.

**Table 1:** Health Care Provider Demographic Information and Occupational Characteristics*^a^*

**Data Collected**	**Brainstorming Survey (Phase 2)**	**Rating Survey** **(Phase 3)**	**PPI Panel** **(Phases 2–5)**
Total, *n*	133	16	5
Age, y, mean (SD)	44 (11)	45 (9)	42 (8)
Sex (m/f)	50/83	8/8	2/3
Country (*n*)	DE (1) Italy (1) UAE (1) NG (1) UK (7) CA (1) BE (3) USA (1) NZ (1) AU (1) IRL (115)	IRL (1) BE (2) Italy (1) UK (2)	IRL (5)
Occupation	PT (118) OS (7) AT (1) PM (2) GP (4) Chiro (1)	PT (14) GP (1) OS (1)	PT (3) OS (1) GP (1)
Work setting	PP (87) PC (32) Private Hosp (7) Public Hosp (27) Sport (3) Other (7) (Education [2]; Research [1]; NS [3]; Cancer Support Center [1])	PP (9) PC (1) Private Hosp (2) Public Hosp (4) Education (1)	PP (3) Public Hosp (1) Private Hosp (1) PC (1)
Experience treating SP, y, mean (SD)	18 (10)	18 (6)	16 (4)
Relevant PG training	MSc (37) PhD (5) Diploma (10) Surgical fellowship (6) NS/none (71)	PhD (2) MSc (8) PG Cert (1) Shoulder fellowship (1) None (4)	MSc (1) PG Cert (1) None (1) Shoulder fellowship (1) NS (1)

*^a^* AT = athletic therapist; AU = Australia; BE = Belgium; CA = Canada; Chiro = chiropractor; DE = Germany; f = female; GP = general practitioner; Hosp = hospital; IRL = Ireland; IT = Italy; m = male; MSc = masters; NG = Nigeria; NS = not specified; NZ = New Zealand; OS = orthopedic surgeon; PC = primary care; PG = postgraduate; PG Cert = postgraduate certificate; PhD = Doctor of Philosophy; PM = physical therapy manager; PP = private practice; PPI = public and patient involvement; PT = physical therapist; SP = shoulder pain; UAE = United Arab Emirates; UK = United Kingdom; USA = United States of America.

**Table 2:** Patient Demographic Information and Shoulder Pain Characteristics*^a^*

**Data Collected**	**Brainstorming Survey**	**Rating Survey**	**PPI Panel**
Total, *n*	21	4	3
Age, y, mean (SD)	52 (13)	60 (15)	52 (6)
Sex (m/f)	5/15	2/2	1/2
Country (*n*)	DE (1) UK (8) IRL (12)	IRL (4)	IRL (3)
Symptom duration (mo), mean (SD)	55 (78)	101 (135)	132 (146)
Diagnosis received	RCRP (14) FS (3) NS (3) SIS (1)	RC tear/injury (3) FS/RCRP (1)	RC tear (2) FS/RCRP (1)
Treatment received (*n*)	PT (17) Acupuncture/DN (4) Analgesics (5) Injection (4) MT (5) Surgery (1) Cupping (1) Hydrodilation (1) Thermal capsulorrhaphy (1) Shockwave (1)	PT (4) MT (1) Injection (1) Thermal capsulorrhaphy (1)	PT (3) MT (1) Injection (1) Thermal capsulorrhaphy (1)
Occupation (*n*)	Education (1) Research (5) IT (2) HCP (7) Manual (1) Engineer (1) NS (4)	Research (2) IT (1) Retired/NS (1)	Research (2) IT (1)

^a^ DE = Germany; DN = dry needling; f = female; FS = frozen shoulder; HCP = health care provider; IRL = Ireland; IT = information technology; m = male; MT = manual therapy; NS = not specified; PPI = public and patient involvement; PT = physical therapy; RCRP = rotator cuff–related pain; SIS = subacromial impingement; UK = United Kingdom.

### Brainstorming Survey (Phase 2)

A total of 154 people completed the survey (HCPs = 155, patients = 21), contributing 548 raw statements (HCP = 487, patients = 61). Round 1 of the preliminary data sorting yielded 692 statements (HCP = 616, patient = 76). Round 2 condensing decisions led to a final statement list of 77 unique priorities (HCP = 45, patient = 32). The raw survey responses, initial editing, condensing decisions, and final statement lists for patients and HCPs are in Supplementary Materials 1 and 2.

### Sorting and Rating (Phase 3)

Twenty-eight participants (HCPs = 24, patients = 4) expressed an interest in further participating in the study, with 8 volunteering as PPI panelists (HCPs = 5, patients = 3) and 20 responding to the rating survey (all 8 panelists; 12 participants). Following preliminary sorting, 14 preliminary categories were presented to the PPI panel (Suppl. Material 3). These were subsequently further condensed to 13 categories with the inclusion of 1 additional priority (within category 4), based on the panel’s feedback. Three overarching domains were identified: (1) Education (6 categories), (2) Patient-centered care (4 categories), and (3) Health care communication (3 categories). An overview of these overarching domains, categories, and stakeholder-specific priorities, including their rated importance, are summarized in Table 3.

**Table 3:** Overarching Domains: Prioritized Categories and Included Priorities Identified by Stakeholders to Facilitate Adherence to Evidence-Based Treatment*^a^*

**Domain**	**Categories** *^b^*	**Group**	**ID** *^c^*	**Priorities** *^d^*	**Importance**		**Proportion of Stakeholders Prioritizing Each Category** *^e^*	
					**Mean (SD)**	**Overall Importance** *^f^*	**Total % (No.)**	**Difference Between Groups (%)**
**Education**	1. Expected progress and recovery timeline	Patient	14	**Patients understood the exact healing process**	4 (0)	**Very important**	**48%** (10)	0%
			4	**Patients understood how long before they can expect to see improvement and the difficulties in predicting this**	3.75 (0.5)	**Very important**		
			6	**Patients understood what to expect in terms of a realistic timeframe for recovery and return to normal activities**	3.5 (0.58)	**Very important**		
			9	**Patients knew how much time off work would be required after surgery**	3.5 (0.58)	**Very important**		
			19	Patients knew that although exercise is not a quick fix, it is the best option for long-term symptom relief	3.25 (0.96)	Important		
			32	Patients provided with before and after visual of improvements achieved with treatment (eg, picture or diagram)	3.25 (0.5)	Important		
		HCP	1	**Patients were informed of level of recovery to expect at various time intervals depending on treatment, setting expectations that improvement with conservative treatment can often take an extended period (eg, minimum 12 wk)**	3.88 (0.34)	**Very important**	**48%** (64)	
			7	**Patients given time-based, meaningful, and realistic goals (eg, relating to sleep, pain management, function)**	3.6 (0.74)	**Very important**		
			21	HCPs and patients understood that injection and surgery do not offer a “quick fix” solution	3.25 (0.86)	Important		
			11	Patients were able to see early signs of progress in relation to pain and range of motion in response to treatments (eg, with exercise therapy)	2.75 (0.58)	Important		
	2. Treatment options and supporting evidence	Patient	17	**Patients provided with clear information on range of treatment options and supporting evidence (eg, pros/cons of alternatives to exercise)**	4 (0)	**Very important**	**38%** (8)	**38%** *^g^*
			21	**Patients provided with information relating to surgical success rates and potential risks**	3.5 (1)	**Very important**		
			20	Patients informed of the benefits of exercise compared to pharmacological treatment (eg, paracetamol)	3.25 (0.96)	Important		
			26	Patients provided with physical therapy that included manual therapy +/− dry needling	3.25 (0.96)	Important		
		HCP	10	**HCPs and patients understood evidence-based treatment recommendations, highlighting to patients the superiority of exercise therapy as first-line treatment rather than hands on therapy, surgery, or injections**	3.69 (0.6)	**Very important**	**75%** (101)	
			13	**HCPs and patients understood research evidence comparing the effectiveness, long-term outcomes, and risks/benefits of treatment options**	3.69 (0.48)	**Very important**		
			20	**If first contact HCPs promoted and set early patient expectations for first-line treatment options; reducing unnecessary imaging and surgical referral**	3.63 (0.5)	**Very important**		
			30	**Information/evidence-based guidelines on shoulder pain management more easily accessible for HCPs and patients**	3.56 (0.51)	**Very important**		
			32	**HCPs understood need for timely referral for first-line treatment, and to provide information about shoulder pain and rehabilitation for those on surgical waiting lists**	3.56 (0.63)	**Very important**		
			34	**HCPs and patients provided clear, concise, and understandable evidence-based information (eg, infographics, videos, handouts)**	3.56 (0.63)	**Very important**		
			4	**HCPs and patients knew the benefits of exercise and its effectiveness as first-line treatment (eg, patient testimonials to support this)**	3.5 (0.63)	**Very important**		
			44	**HCPs and patients understood natural history of atraumatic shoulder pain**	3.5 (0.82)	**Very important**		
			36	PTs provided greater emphasis on education as part of rehabilitation, providing clear rationale behind conservative management and proposed mechanisms underpinning these	3.13 (0.72)	**Important**		
	3. A specific diagnosis	Patient	11	**Patients provided with clear and specific diagnosis for pain and what the injury was**	3.5 (0.58)	**Very important**	38%	20%
			30	Patients provided with visual representation of the body to help understand pain and identify the body part with pain (eg, diagram or picture)	3 (0.82)	Important		
			13	Patients provided with clearer explanation for cause of pain	2.75 (1.26)	Important		
		HCP	9	**Patients provided information relating to diagnosis, nature of pain, and factors that can cause pain**	3.31 (0.79)	**Very important**	18%	
			37	Patients knew shoulder pain difficult to diagnose and often classified as nonspecific	2.75 (0.86)	Important		
			3	HCPs provided education on specific shoulder pathology, identifying the specific structure involved, and explaining reason for pain and how it occurred (eg, using anatomy models/scan)	2 (1)	Somewhat important		
	4. Understanding pain and learning ways to manage it	Patient	*^h^*	**Patients knew what the best sleeping or resting position is to reduce pain**	4 (0)	**Very important**	14%	14%
			5	Patients knew how to prevent a boom-and-bust cycle of pain	2.5 (1)	Somewhat important		
		HCP	5	**Patients were provided guidance on how to manage flare-ups/fluctuations in pain in response to normal daily activities/exercise**	3.56 (0.63)	**Very important**	28%	
			6	**Patients knew what to expect in terms of pain during exercise and what level of exercise-induced pain is acceptable**	3.5 (0.52)	**Very important**		
			31	**HCPs and patients understood pain science, in particular, that pain does not always equal harm or indicate the presence of something sinister (eg, pain during exercise)**	3.44 (0.73)	**Very important**		
			33	**Patients were better informed about how to manage pain, including the role of analgesia to relieve pain and support engagement with rehabilitation**	3.38 (0.81)	**Very important**		
	5. The role of imaging	Patient	27	Patients understood MRI information and medical terminology to enable discussion with HCP	3 (0.82)	Important	14%	12%
			18	Patients provided with early referral for imaging (eg, MRI) to know specific cause for pain and recommended treatment	2.75 (0.96)	Important		
		HCP	35	**HCPs and patients understood imaging is not necessary for successful management and is only appropriate for those nonresponsive to first-line treatment/presence of red flags**	3.81 (0.54)	**Very important**	26%	
			23	**HCPs and patients understood that imaging findings do not always correlate with pain**	3.75 (0.58)	**Very important**		
			24	**Relevance of radiology (if any) and what normal age-related changes would be expected**	3.63 (0.62)	**Very important**		
			40	Patients understood shoulder pain is common and age-related changes are a normal part of life	3.06 (1)	Important		
	6. Risk factors for developing pain and their influence on recovery	Patient	1	Patients knew what activities could increase/decrease risk of developing pain	3 (0.82)	Important	10%	3%
		HCP	38	**Patients provided more education relating to factors that can impact recovery potential and contribute to shoulder pain (eg, stress, obesity, smoking, fear avoidance, comorbidities, etc)**	3.63 (0.5)	**Very important**	7%	
			41	**HCPs and patients had awareness of positive prognostic indicators, such as high self-efficacy and the belief that evidence-based treatment will work**	3.38 (0.89)	**Very important**		
**Patient-centered care**	7. A strong therapeutic relationship	Patient	16	Patients reassured by HCP likelihood of positive treatment response	3.25 (0.96)	Important	48%	**27%** *^g^*
			12	If patients had good rapport and trust in competence of HCP	3.25 (0.96)	Important		
			31	HCP **less** abrupt and rushed with time at end of treatment session	3.25 (0.5)	Important		
		HCP	17	**HCPs and patients developed and maintained strong therapeutic relationship, built on honesty and trust, with HCPs showing empathy and listening carefully to a patient’s whole story, goals, and addressing all fears and concerns**	3.63 (0.62)	**Very important**	21%	
			15	**HCPs acknowledged and explored patient fears and concerns (eg, regarding exercise), and provided reassurance in relation to treatment progress and prognosis**	3.56 (0.63)	**Very important**		
			18	**HCPs took more time to educate patients regarding their condition, imaging findings, and effective treatments, directing them toward reliable information sources**	3.38 (0.72)	**Very important**		
	8. Follow-up and support throughout rehab journey	Patient	15	Patients had better access to additional resources and support to complete exercises (eg, phone app, handouts, visual demonstration, and in-person feedback)	3.25 (1.5)	Important	29%	12%
			24	Patients reviewed frequently by HCP to monitor progress, help motivate and provide feedback, as well as reassurance on right treatment path	3 (0.82)	Important		
		HCP	22	Patients had regular follow-up contact with HCP and provided with resources to support exercise adherence throughout rehabilitation journey (eg, reminders, exercise tracker apps, diaries, printed exercise sheets)	3 (0.52)	Important	17%	
			14	Patients had opportunities to practice exercises with their physical therapist and get ongoing support and feedback throughout their treatment (eg, virtually or face-to-face)	2.94 (0.68)	Important		
	9. Tailoring exercise therapy and preparing for the effort involved	Patient	25	**Patients understood level of commitment required to engage in rehabilitation program**	3.5 (0.58)	**Very important**	24%	12%
			23	Patients prescribed meaningful and convenient exercises for day-to-day life	3.25 (1.5)	Important		
			7	Patients understood importance of following treatment advice and exercise consistency to prevent reoccurrence	3 (0.82)	Important		
		HCP	19	**Patients understood the motivation, effort, and commitment required for exercise therapy, felt a sense of ownership, and had confidence to self-manage their rehabilitation (eg, how to pace, regress, and progress exercises)**	3.5 (0.63)	**Very important**	36%	
			12	**Patients understood benefits and importance of persevering with exercise therapy**	3.5 (0.52)	**Very important**		
			2	**PTs were better able to adapt and tailor rehabilitation programs to suit individual needs, interests, or goals (eg, number of exercises, dosage, available time, level of difficulty, value-based activities, etc)**	3.31 (0.7)	**Very important**		
			8	Patient involvement in exercise prescription by providing choice in what exercises they select and complete	3.19 (0.83)	Important		
			26	Patients had access to high-quality supervised group-based exercise classes (eg, specific shoulder classes, clinical Pilates)	2.19 (0.83)	Somewhat important		
	10. Shared decision-making	Patient	28	Patients provided with HCP opinion to guide what treatment to explore first	3 (0.82)	Important	5%	3%
			29	Patients engaged in collaborative decision on treatment path with HCP	3 (1.41)	Important		
		HCP	29	**Better communication among HCPs/HCPs and patients in relation to discussing evidence-based treatments and outcomes, and supporting shared decision-making and informed treatment choices**	3.38 (0.62)	**Very important**	8%	
**Health care communication and access**	11. Unified message on best management pathway	Patient	2	**Patients knew which HCP to be referred to for treatment and how to access them**	3.75 (0.5)	**Very important**	14%	17%
			3	Patients provided with clear care pathway	3.25 (0.96)	Important		
			10	HCP team providing a unified diagnosis rather than conflicting messages	3.25 (0.96)	Important		
		HCP	25	**HCPs provided unified and consistent message in relation to the best management approach, with greater collaboration and agreement on how best to present evidence-based findings to patients**	3.75 (0.45)	**Very important**	31%	
			16	**Patients provided with clear and unambiguous information on what will improve/exacerbate symptoms (eg, activity modification, healthy lifestyle, etc)**	3.56 (0.63)	**Very important**		
	12. Need for specialist health care provider	Patient	22	**Patients treated by specialist shoulder physical therapist**	4 (0)	**Very important**	10%	3%
		HCP	27	All members of the health care team were considered equal	3 (1.1)	Important	7%	
			45	If patients first contact involved access to specialist/advanced practice shoulder physical therapist with ability directly access imaging, if required	2 (1)	Somewhat important		
	13. Public health care information, policy, and research aligned with current best evidence	Patients	8	Patients had knowledge of long waiting lists for surgery and high likelihood they would need to pay privately	2.25 (1.26)	Somewhat important	5%	6%
		HCP	28	**Public better informed about shoulder pain, role of exercise, benefits of physical activity, why MRI and surgery are not necessarily the answer, and the importance of active involvement in the recovery process (eg, via public/media campaigns)**	3.38 (0.72)	**Very important**	11%	
			42	**There was more information for HCPs to support research exploring the best management, with more financial funding to support these activities**	3.38 (0.5)	**Very important**		
			39	**Health care policy relating to shoulder pain more aligned with current best evidence, with improved financial support for patients to engage in rehabilitation irrespective of public or private health care**	3.31 (0.87)	**Very important**		
			43	More resources available to improve public health literacy from a young age.	3.13 (0.89)	Important		

^a^ apps = software application; HCP = health care provider; ID = identification; MRI = magnetic resonance imaging; PTs = physical therapists.

^b^ Ordered in each domain according to the highest number of patient stakeholders prioritizing each category (ie, Total %No.).

^c^ Identification (ID) numbers correspond to IDs from final statement lists for patients and HCPs (see Suppl. Materials 1 and 2).

^d^ Ordered in each category based on highest rated importance within each group. Those of highest importance highlighted in **bold**.

^e^ Calculated based on number of brainstorming survey respondents who identified priorities within each category. For example, Category 1: 10 Patient identified priorities (ie, 10/21 (total number of patients) = 48%).

^f^ Overall importance based on mean importance ratings (Phase 3) (ie, 1–1.75 = Not important at all, 1.76–2.5 = Somewhat important, 2.51–3.25 = Important, 3.36–4 = Very important).

^g^ Statistically significant difference identified on chi-square test analyses, with a *P* ≤.05 considered statistically significant.

^h^ Additional priority added based on the feedback provided by the public and patient involvement (PPI) advisory board.

### P‌PI Commentary and Feedback

PPI panelists focused on those categories where there was less consensus between stakeholder groups (ie, statistically significant difference or ≥10% difference in contribution between stakeholder groups). Only 2 categories, Categories 2 and 7, showed statistically significant differences between stakeholder groups. For Category 2, more HCPs perceived it as a priority compared to patients (*χ*^2^[1, *N* = 154] = 10.80, *P* = .001) with a medium effect size (*phi* = .29). For Category 7, more patients perceived it as a priority compared to HCPs (*χ*^2^[1, *N* = 154] = 5.53, *P* = .02) with a small effect size (*phi* = −.21). The PPI panel also delved into the potential reasons for less consensus within 6 other categories (ie, Categories 3, 4, 5, 8, 9, 11), showing ≥10% difference in contribution between groups.

#### Category 2: Treatment Options and Supporting Evidence

A high number of stakeholders across both groups contributed to this category, with twice as many HCPs contributing (76% vs 38%, respectively). Some HCP panelists speculated that a possible reason for the importance of this category might be the pressure felt from patients to know “*how do we fix it? Give me the solution*” (HCP1). Although patient panelists concurred with the importance placed on this category, it was noted that for most patients having a “*roadmap for recovery*” (P1) would likely take precedence, accounting for the greater proportion identifying priorities within Category 1 (Expected progress and recovery timeline).

#### Category 3: A Specific Diagnosis

This category was more prevalent among patient respondents (38% vs 18%). Most patient panelists agreed with the “*importance of a diagnosis*” (P3) and concurred with the need to “*understand the why*” (P2) for their symptoms. It was also highlighted that a nonspecific diagnosis can be perceived as “*lazy*” (P2), and that if a specific diagnosis is not possible the HCPs needs to explain “*why and what are the consequences… for my outcomes*” (P3). However, it was also agreed that too much information can be “*overwhelming*” (P1). HCP panelists expressed an appreciation that it was “*human nature*” (HCP1) to want to know the source of symptoms. However, although many HCPs panelists did not feel there was “*much value in a specific diagnosis*” (HCP1) to inform their management, some did highlight that a specific structural diagnosis can be “*easier [for patients] to understand*” (HCP5).

#### Category 4: Understanding Pain and Learning Ways to Manage It

A slightly higher number of HCPs contributed to this category (28% vs 14%). Many HCP panelists felt that the low number of patients prioritizing this category related to many patients “*automatically think[ing] of medication*” (HCP3) as the sole means to manage their pain, with negative patient perception of this approach viewed only as a means to “*mask the pain*” (HCP2) rather than “*fix it*” (HCP1). Some patient panelists also added that they did not wish to be viewed as “*some type of addict just looking for the pain killer*” (P2). Patient panelists also expressed the need to include an additional priority, “*What is the best sleeping or resting position to reduce pain*” (P2), identifying this as their “*biggest problem*” (P3) (Tab. 3, priority Ø).

#### Category 5: The Role of Imaging

A slightly higher number of HCP stakeholders contributed to this category (26% vs 14%). Panelists discussed the challenges presented by the opposing priorities identified. Most HCP panelists recounted their negative experiences of patients being provided with imaging reports that they “*don’t know what to do with*” (HCP3), describing the ensuing difficulties of trying to re-educate patients (ie, explain normal age-related changes). In contrast, other HCP panelists perceived some benefits, providing patients with “*reassurance that there’s nothing seriously wrong*” (HCP2). One of the patient panelists agreed with this point, expressing the perception that imaging can be “*super crucial*” (P2) to rule out “*underlying things*” (P2). However, this was at odds with another of the patient panelist’s perspective, expressing instead their disinterest in relation to imaging findings; “*I didn’t see any relevance in it whatsoever*” (P1), with the panel ultimately highlighting the need for tailored information.

#### Category 7: A Strong Therapeutic Relationship

This category was among the most highly prioritized by patients (48%), equal to that of Category 1 (Expected progress and recovery timeline) (48%), with significantly fewer HCPs prioritizing Category 7 (48% vs 21%). HCP panelists related this to the belief among HCPs that a therapeutic relationship was an “*assumed standard*” (HCP1) and perhaps HCPs did not feel this needed to be explicitly prioritized, especially given that the HCP priorities within this category were all highly rated. In contrast, another HCP noted the failure of some HCPs to prioritize the therapeutic relationship, with some “*not going out of their way*” (HCP4) to make an effort to build these relationships. Another explanation related to the impact of the COVID-19 pandemic, with HCPs perhaps deprioritizing this aspect of care with HCPs simply “*not seeing them regularly enough to build a relationship*” (HCP4). Patient panelists expressed consensus in relation to the importance of this category, noting the importance of having a HCP “*that I can like and I trust*” (P2).

#### Category 8: Follow-Up and Support Throughout Rehab Journey

A slightly higher number of patients contributed to this category (29% vs 17%). HCP panelists commented on the lack of influence they feel they have on this aspect of care, highlighting that while HCPs “*know it’s important, it’s largely impractical*” due to time restraints and waiting lists (HCP4), referencing also to the possible impact of emerging research with “*a trend towards less follow-up*” (HCP3). Two HCP panelists commented that they felt the importance attributed to this by some patients related to a “*sense of entitlement*” (HCP1) to follow-up and high levels of support for those paying private health insurance. Patient panelists felt that these differences related more to the fact that while HCPs are treating multiple patients, for the patient, they are probably only interacting with the 1 HCP, with the follow-up support forming “*a larger part of the patient’s world*” (P1). Another point raised related to the perceived greater need for support for adherence to physical therapy exercises, considered “*really boring*” (P3) by 1 patient panelist.

#### Category 9: Tailoring Exercise Therapy and Preparing for the Effort Involved

A higher percentage of HCP stakeholders prioritized this category compared to patients, the third highest within the HCP stakeholder group (36% vs 24%). Despite both groups highly rating the importance of the priorities within this category, conflicting needs were identified between groups, with patient priorities appearing more passive, referring to “convenience” (priority 23) and “following” HCP advice (priority 7), compared to HCP priorities referring to “ownership” (priority 19) and active “involvement” (priority 8). HCP panelists commented that the effort required to engage in exercise must be communicated in a “non-threatening” (HCP1) manner, while patient panelists expressed the need for HCPs to prepare them for the “discipline” (P1) required.

#### Category 11: Unified Message on Best Management Pathway

A high percentage of HCP stakeholders contributed to this category (31% vs 14%), with priorities all rated highly important by HCPs. HCPs panelists commented that they were unsurprised by these findings, stating that HCPs are likely more “*acutely aware of discrepancies*” (HCP1) within HCP treatment approaches. Another HCP panelist expressed the belief that patients are less interested in whether HCPs are adopting similar approaches “*as long as the approach works for them*” (HCP3). One of the patient panelists postulated that the mismatch in treatment approaches among HCP is likely due to a lack of supporting research evidence, empathizing with the challenge this poses and the potential it has to “*undermine confidence*” (P3) if HCPs admit “*how unclear*” the research evidence is (P3). Another panelist highlighted the view that patients expect to be provided with a “*customized*” (P1) treatment, not a “*one-size approach*” (P1), and that the uniformity in practice sought by HCPs is perhaps not what patients are looking for.

### Concept Map

A number of linkages between categories were identified by the panel, representing the perceived directional relationship between the categories. These linkages were illustrated within a draft map circulated to the panel for feedback (Suppl. Material 4). Feedback mainly focused on the need to reduce and simplify relationships between categories, such as using 1 bidirectional line to illustrate opposing viewpoints (eg, Categories 2 and 3), also identifying additional relationships between categories (eg, Categories 7 and 11), with amendments made to reflect these proposed changes (Suppl. Material 5). As illustrated within this revised map (Suppl. Material 5), there were 3 categories perceived by the PPI panel to be of pivotal importance, as depicted by the greatest number of identified linkages associated with these categories (ie, education on treatment options (Category 2), a strong therapeutic relationship (Category 7), and a unified message (Category 11) from HCPs on best management pathway). However, the consensus was that the map was still visually difficult to interpret given the number/crossover of linkages depicted. Therefore, the final concept map illustrates the prioritized categories, with linkages removed, with the 3 categories perceived by the PPI panel to be of key importance highlighted in black (Fig. 3).

## DISCUSSION

### Summary of Key Findings

This study identified a broad spectrum of patient and HCP priorities that are perceived to foster patient and HCP adherence to evidence-based treatment for shoulder pain. There were a number of areas of overlap between stakeholder groups, namely, relating to the prioritization of education of expected progress and recovery timelines as well as treatment options and supporting evidence. Points of divergence were also identified, with patients prioritizing education related to a specific diagnosis, and HCPs instead prioritizing understanding pain and learning ways to manage it, as well as the role of imaging. Relating to patient-centered care, the most prioritized category among patients was a strong therapeutic relationship, followed by follow-up and support throughout the rehab journey. Although a strong therapeutic relationship was rated by HCPs as highly important, a lower percentage of HCPs identified this as a priority, instead prioritizing tailoring exercise therapy and preparing for the effort involved. In relation to health care communication, few patients contributed to this category, with HCPs prioritizing a unified message on best management. Less than 15% of stakeholders identified priorities within the remaining categories, with as few as 7% of the total stakeholders prioritizing shared decision-making. As evident from the concept map, education relating to treatment options and supporting evidence, a strong therapeutic relationship, and a unified message on best management were perceived by the PPI panel as being of pivotal in facilitating multistakeholder adherence to evidence-based treatment.

### Comparison With Existing Literature

A key finding of this study was the identification across both stakeholder groups of the need for education. This finding is unsurprising considering that 2 of the innate psychological needs that must be satisfied to support intrinsic motivation to adhere is the need for competence and autonomy.^14^ The most important priorities identified by stakeholders within this domain included education related to surgery and exercise therapy. In relation to knee osteoarthritis, similar findings have been identified, with stakeholders also prioritizing education relating to surgery and/or exercise.^30^^,^^31^ Consistent associations have been shown between positive patient recovery expectations and improved patient-reported outcomes for first-line treatments of shoulder pain, such as physical therapy.^36^ Given the identified need among stakeholders for education relating to expected progress and recovery timelines, these findings highlight the importance of providing such education to patients to facilitate realistic expectations. The importance that patients with shoulder pain place on recovery timelines has also been identified in previous qualitative research.^37^ However, as evidenced in a recent qualitative evidence synthesis, there is much ambiguity among stakeholders in relation to recovery timelines for this condition, making it challenging for HCPs to provide clear education.^9^

The relevance of a specific diagnosis for rotator cuff–related shoulder pain is something that has been extensively debated.^38^ While there are some researchers urging clinicians to desist from identifying a patho-anatomical structure to diagnose shoulder pain,^39^ many recent high-quality clinical practice guidelines for MSK shoulder pain continue to highlight the importance of differentiating between patho-anatomical shoulder diagnoses to guide treatment.^40^^,^^41^ Patient stakeholders within the current study prioritized education relating to a specific diagnosis. These findings echo those of previous research, suggesting that a specific diagnosis could be critical to a patient’s understanding of their condition,^8^^,^^42^ with evidence of widespread biomechanical beliefs underpinning this understanding, influencing the education provided to and sought by patients.^12^ In contrast, within the general field of MSK pain, although some stakeholders continue to identify a need for diagnostic imaging to locate “the source” of pain,^43^ other researchers in the field of MSK pain express some concern over the potential for diagnostic overmedicalization.^44^ It is clear that more evidence is needed to reach consensus on the necessity of having a specific diagnosis in the field of MSK pain management. However, these findings echo the interpretations drawn from the PPI panel within the current study, highlighting the need for education relating to diagnosis and imaging to be tailored to the individual’s needs.

Research shows that inconsistent communication from HCPs can lead to patient confusion about shoulder pain diagnosis, prognosis, and treatment options. To improve patient education, clear and unbiased communication of research evidence is crucial.^11^^,^^12^ Decision aids have been suggested as a way to support shared decision-making and improve treatment delivery, but their effectiveness varies, with a recent study indicating that a decision tool had no impact on patient intentions regarding shoulder surgery.^45^ Despite recommendations for a patient-centered approach,^46^ and the availability of many conceptual models designed to support shared decision-making in clinical practice,^47^ there is little evidence of the effective integration of a shared decision-making approach in shoulder pain management,^12^ with only 7% of stakeholders prioritizing it in our study.

Another key finding of this study was the significant variation between stakeholder groups prioritizing a strong therapeutic relationship. Although a low proportion of HCPs identified priorities relating to this category, both HCPs conducting the importance rating survey and PPI panelists identified this relationship to be of pivotal importance. The centrality of a strong therapeutic relationship has been highlighted previously by stakeholders across a range of musculoskeletal disorders,^48^^,^^49^ including shoulder pain, considered to improve treatment adherence,^10^ decision-making,^12^ and trust in pain education.^42^ These findings are further supported given the known psychological need for “relatedness” to enhance self-motivation to adhere.^14^ One HCP panelist proposed that the reason for the low number of HCPs identifying priorities within this category was that it was an “*assumed standard*” and as such did not require explicit prioritization. However, the lack of integration of a shared decision-making process and the identification of negative assumptions and stereotyping among HCPs within previous research studies would question the accuracy of such assumptions.^12^

### Strengths and Limitations

One of the main strengths of this study is the inclusion of a PPI panel, shown to strengthen the quality, relevance, and impact of research.^50^ The inclusion of a PPI panel, and the subsequent triangulation of researchers and PPI co-researcher perspective, as well as the member-checking stage to verify completeness and accuracy of interpretations, enhanced the trustworthiness and integrity of this research, providing greater credibility and confirmability of the results.^35^^,^^51^ While there are many advantages of using a concept map, such as reducing the volume of the data presented, pictorially displaying linkages and maintaining the richness of the participants’ meaning, one of the main disadvantages is its growing complexity as more linkages are added.^52^ This was echoed by 1 of the patient panelists who suggested that a more simplified map would aid patient interpretation. Although there were fewer patient survey respondents, the inclusion of 3 patient PPI panelists contributed further to the attainment of data saturation. The authors utilized multiple recruitment strategies to include a broad spectrum of stakeholder views. Nonetheless, a large proportion of survey respondents were physical therapists (ie, 89% within brainstorming survey and 87% within rating survey), as were 4 of the authors, as well as 3 of the PPI panel, and as such, responses and the interpretations drawn from the findings of this study may have been biased by this professional view. However, the multistakeholders involved in the PPI panel and research team, as well as the continuous verification process underpinning this research study (ie, regular discussions and debriefing sessions seeking critical commentary and feedback), helped facilitate reflexivity among the research team, together enhancing dependability and confirmability,^53^ and thus strengthening the reliability and validity of study findings.^54^ Survey respondents were located across 11 countries; however, the PPI panelists were all located in Ireland. Although this may potentially limit the transferability of the interpretations drawn by the PPI panel,^55^ as 2 of the authors involved in the PPI virtual meeting were Canadian (V.L., J.S.), and 2 of the patient PPI panelists (P2, P3) had re-located from other countries (eg, Canada, USA), it was felt that international viewpoints were also considered in the interpretations drawn. That said, future research is certainly warranted to investigate if cultural differences in perceived priorities to adherence are identified within health care practices in other countries.

### Implications for Clinical Practice and Research

The authors encourage HCPs to familiarize themselves with the identified educational priorities of patients, understanding the need to personalize the level of detail and volume of information they provide thereby facilitating a more tailored approach. However, simply having an awareness of such educational priorities or evidence-based treatment recommendations is not of itself sufficient to elicit lasting improvements in clinical practice.^56–58^ There is an identified need for the development of a KT educational resource to provide stakeholders with up-to-date education relating to these identified educational priorities. The inclusion of a facility to identify patient information needs was identified by the PPI panel as a crucial step in this process, enabling a more tailored approach. Without tailoring information to the individual and acknowledging the ineffectiveness of a “one size fits all” approach to education,^42^ the authors suggest that future KT strategies will continue to fall short. It is also clear from the findings of this study that HCPs do not appear to consciously prioritize the pivotal role of the therapeutic relationship and engagement in shared decision-making. HCPs are therefore urged to actively prioritize these key aspects of patient centeredness, with HCPs, employers, and policymakers considering what training and consultation time would be required to facilitate meaningful shared decision-making practices. Future research should explore more effective ways to support the improved integration of all aspects of patient-centered care into clinical practice. It should also investigate what differences, if any, exist in stakeholder priorities and their perceived relationships across different countries and health care settings, helping to further inform the development and universality of future KT resources.

## CONCLUSIONS

A broad spectrum of priorities was identified across both patient and HCP groups. While there were many areas of overlap and agreement in terms of identified educational needs, specific points of divergence centered around the identified need by patients to have a specific diagnosis and by the degree to which stakeholder groups prioritized a strong therapeutic relationship. Although the PPI panel considered establishing a strong therapeutic relationship to be of pivotal importance, greater emphasis must be placed on integrating this aspect of patient-centered care and shared decision-making into clinical practice to further facilitate multistakeholder adherence to evidence-based treatment recommendations. Educational resources for shoulder pain diagnosis and management should be tailored, with cohesive messaging, to support first-line treatments like exercise therapy for MSK shoulder pain.

## Supplementary Material

2023-0709_R1_Supplementary_Material_pzae176

## Data Availability

The authors confirm that the data supporting the findings of this study are available within the article [and/or] its Supplementary Material.
