# Supplementary material for: Fostering Adherence to Evidence-Based Care in the Management of Musculoskeletal Shoulder Pain: A Mixed-Methods Study
Source: Phys Ther. 2024 Dec 16;105(3):pzae176. doi: 10.1093/ptj/pzae176 (PMC11955010; doi:10.1093/ptj/pzae176)

Supplementary Material 1. Preliminary sorting of raw patient survey responses, including initial editing (round 1), condensing decisions (round 2), and final statement list for inclusion in importance rating survey

Round 1: Editing – Patient brainstorming responses, edits, and revised priorities (R1)

Patient No.	Response No.	Original Response	Revised priority	Priority ID after R1
1	1.1	Back and shoulder pain were addressed on prenatal, antenatal, or breastfeeding courses in which i participated.	Education about ways to prevent shoulder pain provided to those at risk of developing (e.g., to expectant mothers as part of the antenatal information/courses available)	1.1
	1.2	The public health nurse who visted after baby was born had been better enabled to refer me to appropriate resources.	Ability of public health nurses to refer for appropriate resources.	1.2
2	2.1	I would like a clear pathway of care	Clear pathway of care	2.1
	2.2	I didn't realise how long it would take for any improvement	Understanding of expectant timeline for improvement	2.2
3	3.1	I had better understanding of how difficult it is to predict improvement	Better understanding of how difficult it is to predict improvement	3.1
	3.2	If I knew how to better stop boom and bust	Knowledge of how to prevent boom and bust cycle of pain	3.2
	3.3	If I had realistic timings so understood when takes so long	Provision of realistic recovery timings to be better prepared for when it can take longer	3.3
4	4.1	I followed all treatment advice given	The importance of following all treatment advice given	4.1
	4.2	I paid for private surgery as NHS treatment was a long waiting list and meant I needed time off work	The need to pay for surgery to avoid long waiting lists	4.2a
			Time off work required post-surgery	4.2b
	4.3	I was told it was different problems each time I was seen	If HCPs communicated less conflicting information and were more unified in their diagnosis	4.3
5	5.1	Not explained - gp just said that it was inflamed	Provided with a better explanation by their GP	5.1
	5.2	I have a good repor with physio and was going to him anyways due to previous neck injuries	Having a good rapport and previous positive experience with physiotherapist for another injury	5.2
6	6.1		Clearer idea of the cause of my pain	6.1a

		... I had a clearer idea of the cause of my pain and shoulder instability.	Clearer idea of my diagnosis	6.1b
	6.2	.... If I was reassured that sticking with my exercise programme will prevent reoccurrence.	Reassurance that sticking with exercise programme will prevent reoccurrence	6.2
7	7.1	If was explained exactly what the injury was and the healing process	Explanation of exactly what the injury was	7.1a
			Explanation of exact healing process	7.1b
	7.2	there was an online resource or app with exercise tips and instructions for the different stages of recovery	Online resource or AOO to provide exercise tips and instructions for the different stages of recovery	7.2
	7.3	after seeing a physiotherapist, I felt much happier that the issue was fixable	Reassurance from physiotherapy that issue was 'fixable'	7.3
8	8.1	If i had been given realistic.time.frame and reassurance	Given realistic timeframe	8.1a
			Given reassurance	8.1b
	8.2	Given options for treatment and evidence for same	Given treatment options available	8.2a
			Given evidence supporting different treatment options available	8.2b
	8.3	If i had a realistic plan for return to my sport (swimming)	Given realistic plan for returning to sport	8.3
9	9.1	It would be easier to follow treatment advice if I had recieved an MRI sooner and new the specific cause of my shoulder pain	Earlier imaging (MRI) to know specific cause of pain	9.1
	9.2	It would be easier for me to follow treatment advice if I had a specific diagnosis sooner	Provided with specific diagnosis at earlier time point	9.2
	9.3	It would be easier for me to follow treatment advice if i had known the likley hood of improvment in my treatment plan	Knowledge of likelihood of improvement with treatment plan	9.3
	9.4	It would be easier for me to follow treatment advice if I was refered the right treatment because excercise therapy was the only treatment that showed long term symptom relief	Being referred for the right treatment that provided long-term symptom relief, such as exercise therapy	9.4

	9.5	It would be easier for me to follow treatment advice if i knew the benefits of strenghtening the surrounding muscle vs pharmacological treatment	Knowledge of the benefits of a strengthening exercise programme compared to pharmacological treatment	9.5
10	10.1	How important it is to do the exercises the physio gives you, to do them regularly and consistently, and for enough time to enable them to be effective	Importance of adherence with exercises prescribed by physiotherapist	10.1
			Importance of doing prescribed exercises regularly and consistently	10.2
			Knowledge of the importance of giving exercise therapy enough time to be effective	10.3
	10.2	I had been told surgery was in fact contraindicated in individuals with my problems	Knowledge that surgery is contraindicated	10.2
	10.3	How important it is to see a physio who specialises in shoulders	Importance of seeing physiotherapist who specialises in shoulders	10.3
	10.4	Long standing good shoulder health (what to avoid, what to actively do) in everyday activities	Knowledge of what to avoid in everyday activities to ensure long standing good shoulder health	10.4a
			Knowledge of what to actively do to support long standing good shoulder health	10.4b
11	11.1	I have a great therapeutic alliance with my hcp, and they show they really cared	Having a great therapeutic alliance with treating HCP, who shows they really care	11.1
	11.2	it was explained to me that surgery doesn’t always work and carries risks	Education that surgery doesn’t always work and carries risks	11.2
	11.3	the exercises are meaningful and can be carried out in my day to day life	Exercises were prescribed that were meaningful	11.3a
			Exercises could be carried out in day-to-day life	11.3b
	11.4	I am reviewed often enough that my motivation is boosted and my progress checked	Frequent review by HCP to boost motivation	11.4a
			Frequent review by HCP to check progress	11.4b
	11.5	It is explained to me that exercises aren’t a quick fix, but the better fix long term	Explained that exercises aren’t a quick fix, but they are the better fix in the long-term	11.5
12	12.1	If it was explained to me at the outset, when the injury occurred, as to what I needed to commit to as a programme for rehabiliation	Early education from the outset on the commitment required to engage in a programme of rehabilitation	12.1
	12.2	exercise therapy was presented in a documented form without me having to try and interpret this at clinics I was attending	Presentation of exercise therapy in a documented form	12.2

13	13.1	I had frequent Physiotherapy sessions with good manaul therapy carried out	Frequent physiotherapy with good manual therapy carried out	13.1
	13.2	Consistent deep dry needling	Consistent deep dry needling	13.2
14	14.1	Given more information on the condition	Given more information on the condition	14.1
	14.2	Alternatives to exercises were explained	Alternatives to exercise were explained	14.2
15	15.1	It would have been useful to have had the ultrasound after the initial accident, not 18 months after when given exercises weren't helping. It was only after this that different exercises helped.	Having an ultrasound would be more useful immediately after the initial accident, rather than 18 months following failed exercise therapy, as it was only after this that different exercises helped	15.1
	15.2	It was explained after the ultrasound what was wrong and what treatment would be helpful, surgery was not deemed to be useful for this injury	It was explained after the ultrasound scan what was wrong and what treatment would be helpful, in this case surgery was not recommended	15.2
16	16.1	I'm a researcher I researched the crap out of my shoulder. I understood the mri info and could talk to the dr and physio with medical terminology	An understanding of MRI information and the ability to talk to healthcare providers with medical terminology	16.1
	16.2	I regret having the thermal capsulorrhaphy but it was needed.		Excluded after initial review.
	16.3	Honestly I'm lazy at times. I know I need to do it abd I trust some physios more than others. But I get lazy	Feeling lazy and lacking motivation to do exercises	16.2a
			I trust some physiotherapists more than others, and this influences motivation to do exercises	16.2b
17	17.1	WANTED EXPLANATION WHY I HAD SUCH EXCRUCIATING PAIN IF NOT BROKEN	Wanted an explanation why I had such excruciating pain if I didn't break a bone	17.1
	17.2	WANTED REASSURANCE THAT I WOULD GET BETTER EVENTUALLY IF I EXERCISED	Reassurance that I will get better eventually if I exercises	17.2
	18.1	My physio clearly outlined the range of options for my condition and give his professional opinion as to which option(s)should be explored first	Clear information provided by Physiotherapist on range of treatment options available for condition	18.1a
18			Physiotherapist provided opinion on which treatment option to explore first	18.1b

	18.2	The establishment of trust in the competence of my physio is of utmost importance	Establishing trust in the level of competency of treating Physiotherapist is of utmost importance	18.2
	18.3	Patient feedback, and modeling of exercises, is important to me	Feedback and modelling of exercises	18.3
	18.4	When the treatment regime is lengthy, and it can be easy to "give up", it is vital that the physio provides hope - and evidence - that we are, collaboratively on the right path	When the treatment regime is lengthy, it is vital that the physiotherapist provides hope to motivate to not “give up”	18.4a
			When the treatment regime is lengthy, it is vital that the physiotherapist provides evidence on right treatment path	18.4b
			Collaboratively decide if on right treatment path	18.4b
	18.5	An actual diagram, or pictorial representation of the body,would have been a helpful aid	A visual representation of the body would have been helpful (e.g., diagram, picture)	18.5
19	19.1	If I’d known that there was light at the end of the tunnel with exercise therapy	Known there was light at the end of the tunnel with exercise therapy	19.1
	19.2	If I’d had clear milestones/expectations of improvements along the way with exercise therapy	Clear milestones/expectations of improvement along the way with exercise therapy	19.2
20	20.1	had advice from a physiotherapist with expertise in this area.	Advice from a Physiotherapist with expertise in this area	20.1
	20.2	if I actually had sound advice besides recommendation for surgery.	If I actually had sound advice besides recommendation for surgery.	20.2

21	21.1	a pictorial full-body diagram (back and front) would have been beneficial at the start of therapy, whereby the "offending" body part was identified in relation to the whole body	A pictorial full-body diagram at the start of therapy whereby the “offending” body part was identified.	21.1
	21.2	Having all the treatment options laid out, and explained as to their respective benefits and shortcomings, was a decisive help. This also helped to establish a good therapeutic alliance from the outset of treatment	Having all the treatment options laid out with an explanation of their respective benefits and shortcomings.	21.1a
			Having my treatment options and their benefits and shortcomings helped establish a goof therapeutic alliance from the outset of treatment.	21.1b
	21.3	Regular positively-focused feedback on progress - and mapping/showing clear evidence of improvement over the course of treatment, is of significant benefit, and key to keeping me (the client/patient) engaged with the exercise regime - which can ofter be disheartening when doing exercises at home!	Regular positively focused feedback on progress	21.3a
			Mapping/showing clear evidence of improvement over the course of treatment is of significant benefit & was key to helping me keep engaged with the exercise regime which was often disheartening when doing exercises at home.	21.3b
	21.4	The provider (physio etc) must be subtle about session-endings, bring the session to a natural phased ending, and not "rush" the patient out the door - akin to "your time is up today"	The healthcare provider bringing the treatment session to a natural phased ending and not rushing me out because the session time was up.	21.4
	21.5	At the end of treatment programme, to outline in, say, pictorial format the actual gains achieved as a result of therapy - a kind of "before and after" pictorial/diagram overview	Having a before and after pictorial/diagram overview at the end of treatment to outline the actual gains achieved	21.5

Round 2: Condensing decisions – grouping of revised patient educational priorities (R1), based on thematic similarities, creating final statements (R2), each reflecting the overall interpretation of grouped R1 priorities.

Priority ID after R1	Revised priorities after R1	Other R1 priorities that are same/similar	Final statement after R2	Final Statement ID
1.1	Education about ways to prevent shoulder pain provided to those at risk of developing (e.g., to expectant mothers as part of the antenatal information/courses available)	10.4a Knowledge of what to avoid in everyday activities to ensure long standing good shoulder health<br>10.4b Knowledge of what to actively do to support long standing good shoulder health	I knew what everyday activities could increase or decrease the risk of me developing shoulder pain	1
1.2	Ability of public health nurses to refer for appropriate resources.		If I knew which healthcare provider I should be referred to for appropriate treatment, and how I can access them.	2
2.1	Clear pathway of care		I had a clear pathway of care	3
2.2	Understanding of expectant timeline for improvement	3.1 Better understanding of how difficult it is to predict improvement<br>8.1a Given realistic timeframe<br>19.2 Clear milestones/expectations of improvement along the way with exercise therapy	I knew how long to expect to see an improvement, and how difficult this is to predict.	4
3.2	Knowledge of how to prevent boom and bust cycle of pain		I knew how to prevent a boom-and-bust cycle of pain	5
3.3	Provision of realistic recovery timings to be better prepared for when it can take longer	8.3 Given realistic plan for returning to sport	I knew what a realistic timeframe is to expect in relation to recovery and return to normal activities. (e.g., sport)	6
4.1	The importance of following all treatment advice given	6.2 Reassurance that sticking with exercise programme will prevent reoccurrence<br>10.1a Importance of adherence with exercises prescribed by physiotherapist	I understood how important it is it to follow treatment advice and to be consistent with exercises prescribed to prevent reoccurrence.	7

		10.1b Importance of doing prescribed exercises regularly and consistently		
4.2a	The need to pay for surgery to avoid long waiting lists		I knew there would be a long waiting list for surgery, and I would need to pay privately	8
4.2b	Time off work required post-surgery		I knew how much time off work is needed after surgery	9
4.3	If HCPs communicated less conflicting information and were more unified in their diagnosis		My healthcare provider team was unified in their diagnosis and didn't provide conflicting information	10
5.1	Provided with a better explanation by their GP	6.1b Clearer idea of my diagnosis<br>7.1a Explanation of exactly what the injury was<br>9.2 Provided with specific diagnosis at earlier time point<br>14.1 Given more information on the condition	I was provided with a clear and specific diagnosis for my pain or what the injury was.	11
5.2	Having a good rapport and previous positive experience with physiotherapist for another injury	11.1 Having a great therapeutic alliance with treating HCP, who shows they really care<br>18.2 Establishing trust in the level of competency of treating Physiotherapist is of utmost importance<br>16.2b I trust some physiotherapists more than others, and this influences motivation to do exercises	I had a good rapport and trust in the competence of my healthcare provider	12
6.1a	Clearer idea of the cause of my pain	17.1 Wanted an explanation why I had such excruciating pain if I didn't break a bone	I was clear on the cause of shoulder pain	13
7.1b	Explanation of exact healing process		I understood the exact healing process	14
7.2	Online resource or APP to provide exercise tips and instructions for the different stages of recovery	12.2 Presentation of exercise therapy in a documented form<br>18.3 Feedback and modelling of exercises	I had access to additional resources and support to complete my exercise programme (e.g., phone application, handouts, visual demonstration, and feedback in-person)	15
7.3	Reassurance from physiotherapy that issue was 'fixable'	8.1b Given reassurance<br>9.3 Knowledge of likelihood of improvement with treatment plan<br>17.2 Reassurance that I will get better eventually if I exercises	I was reassured from my healthcare provider of the likelihood of improvement in response to my treatment plan	16

		19.1 Known there was light at the end of the tunnel with exercise therapy		
8.2a	Given treatment options available	8.2b Given evidence supporting different treatment options available<br>14.2 Alternatives to exercise were explained<br>18.1a Clear information provided by Physiotherapist on range of treatment options available for condition<br>21.1b Having all the treatment options laid out with an explanation of their respective benefits and shortcomings.	I was provided with clear information on the range of treatment options available and the evidence supporting these.	17
9.1	Earlier imaging (MRI) to know specific cause of pain	15.1 Having an ultrasound would be more useful immediately after the initial accident, rather than 18 months following failed exercise therapy, as it was only after this that different exercises helped<br>15.2 It was explained after the ultrasound scan what was wrong and what treatment would be helpful, in this case surgery was not recommended	I was referred early for imaging (e.g., MRI) to know specific cause of pain and what treatment was most recommended.	18
9.4	Being referred for the right treatment that provided long-term symptom relief, such as exercise therapy	10.1c Knowledge of the importance of giving exercise therapy enough time to be effective<br>11.5 Explained that exercises aren't a quick fix, but they are the better fix in the long-term	I knew that exercise therapy does not provide a quick fix, but it can provide the best long-term relief of symptoms.	19
9.5	Knowledge of the benefits of a strengthening exercise programme compared to pharmacological treatment		I knew what the benefits of a strengthening exercise programme are compared to pharmacological treatment (e.g., paracetamol).	20
10.2	Knowledge that surgery is contraindicated	10.2 If I actually had sound advice besides recommendation for surgery<br>11.2 Education that surgery doesn't always work and carries risks	I had knowledge of the success rates of surgery, the risks involved, and that it is contraindicated.	21
10.3	Importance of seeing physiotherapist who specialises in shoulders	20.1 Advice from a Physiotherapist with expertise in this area	I was treated by a Physiotherapist who specialises in shoulder pain management	22
11.3a	Exercises were prescribed that were meaningful	11.3b Exercises could be carried out in day-to-day life	I had exercises prescribed that were more meaningful and could be carried out in my day-to-day life	23

11.4a	Frequent review by HCP to boost motivation	11.4b Frequent review by HCP to check progress<br>16.2a Feeling lazy and lacking motivation to do exercises<br>18.4a When the treatment regime is lengthy, it is vital that the physiotherapist provides hope to motivate to not "give up"<br>18.4b When the treatment regime is lengthy, it is vital that the physiotherapist provides evidence on right treatment path<br>21.3a Regular positively focused feedback on progress	I was reviewed frequently by my healthcare provider to monitor and provide positive feedback on my progress; helping to motivate and reassure me I was on right treatment path.	24
12.1	Early education from the outset on the commitment required to engage in a programme of rehabilitation		I understood the level of commitment required when engaging in a programme of rehabilitation	25
13.1	Frequent physiotherapy with good manual therapy carried out	13.2 Consistent deep dry needling	I had physiotherapy that included good manual therapy and/or dry needling	26
16.1	An understanding of MRI information and the ability to talk to healthcare providers with medical terminology		I understood MRI information and medical terminology to enable me to talk to healthcare providers.	27
18.1b	Physiotherapist provided opinion on which treatment option to explore first		I had the opinion of my healthcare provider on what treatment options to explore first.	28
18.4c	Collaboratively decide if on right treatment path		I decided what was the right treatment path collaboratively with my healthcare provider.	29
18.5	A visual representation of the body would have been helpful (e.g., diagram, picture)	21.1 A pictorial full-body diagram at the start of therapy whereby the "offending" body part was identified	I had a visual representation of the body to help with my understanding and to identify the offending body part (e.g., diagram or picture).	30
21.4	The healthcare provider bringing the treatment session to a natural phased ending and not rushing me out because the session time was up.		My healthcare provider was less abrupt at the end of the treatment session and didn't rush me out because my time was up.	31
21.5	Having a before and after pictorial/diagram overview at the end of treatment to outline the actual gains achieved		I had a before and after visual overview to outline the actual improvements achieved at the end of treatment (e.g., picture or diagram).	32

Patient Final Statement List (for inclusion in importance rating survey)

Final Statement ID	Final Statements
1	I knew what everyday activities could increase or decrease the risk of me developing shoulder pain
2	If I knew which healthcare provider I should be referred to for appropriate treatment, and how I can access them.
3	I had a clear pathway of care.
4	I knew how long to expect to see an improvement, and how difficult this is to predict
5	I knew how to prevent a boom-and-bust cycle of pain
6	I knew what a realistic timeframe is to expect in relation to recovery and return to normal activities. (e.g., sport)
7	I understood how important it is it to follow treatment advice and to be consistent with exercises prescribed to prevent reoccurrence
8	I knew there would be a long waiting list for surgery, and I would need to pay privately
9	I knew how much time off work is needed after surgery
10	My healthcare provider team was unified in their diagnosis and didn't provide conflicting information
11	I was provided with a clear and specific diagnosis for my pain or what the injury was
12	I had a good rapport and trust in the competence of my healthcare provider.
13	I was clear on the cause of shoulder pain
14	I understood the exact healing process
15	I was reassured from my healthcare provider of the likelihood of improvement in response to my treatment plan
16	I had access to additional resources and support to complete my exercise programme (e.g., phone application, handouts, visual demonstration, and feedback)
17	I was provided with clear information on the range of treatment options available and the evidence supporting these.
18	I was referred early for imaging (e.g., MRI) to know specific cause of pain and what treatment was most recommended.
19	I knew that exercise therapy does not provide a quick fix, but it can provide the best long-term relief of symptoms.
20	I knew what the benefits of a strengthening exercise programme are compared to pharmacological treatment (e.g., paracetamol).
21	I had knowledge of the success rates of surgery, the risks involved, and that it is contraindicated.
22	I was treated by a Physiotherapist who specialises in shoulder pain management
23	I had exercises prescribed that were more meaningful and could be carried out in my day-to-day life
24	I was reviewed frequently by my healthcare provider to monitor progress; help motivate me and provide me with feedback.
25	I understood the level of commitment required when engaging in a programme of rehabilitation
26	I had physiotherapy that included good manual therapy and/or dry needling
27	I understood MRI information and medical terminology to enable me to talk to healthcare providers.
28	I had the opinion of my healthcare provider on what treatment options to explore first.
29	I decided what was the right treatment path collaboratively with my healthcare provider.
30	I had a visual representation of the body to help with my understanding and to enable me to identify the offending body part (e.g., diagram or picture).
31	My healthcare provider was less abrupt at the end of the treatment session and didn't rush me out because my time was up.
32	I had a before and after visual overview to outline the actual improvements achieved at the end of treatment (e.g., picture or diagram).

Supplementary Material 2. Preliminary sorting of original healthcare provider (HCP) survey responses, including initial editing (round 1), condensing decisions (round 2), and final statement list for inclusion in importance rating survey

Round 1: Editing – HCP original brainstorming responses, editing, and revised priorities (R1)

HCP No.	Response No.	Original Response	Revised priority	Priority ID after R1
1	PT1.1	Patients were better informed on what to expect in terms of function at various intervals along their rehab journey.	What level of functional return to expect at various intervals along the rehabilitation journey.	1.1
	PT1.2	Patients were better informed on what to expect in terms of timeline of healing + rehab + return to sport	What to expect in terms of healing times.	1.2a
			What to expect in terms of rehabilitation timeline.	1.2b
			What to expect in terms of timeline to return to sport.	1.2c
	PT1.3	Patients were informed regarding long term pain + function e.g 6 months 12 months 18 months post intervention + comparisons made between treatment options e.g. exercise vs surgery.	What can be expected in terms of pain and function in the long-term when comparing between treatment options available.	1.3
	PT1.4	Physiotherapists were better able to adapt/individualise rehab to focus on sport/function specific requirements	Physiotherapists were better able to adapt and individualize rehabilitation	1.4
2	PT2.1	use shoulder model to educate patient on anatomy and how exercise therapy helps this	Use of anatomy models to provide education in relation to shoulder anatomy	2.1a
			How exercise can treat the condition.	2.1b
	PT2.2	explain pain patterns and what to expect during exercise	Explanation in relation to expected pain patterns	2.2a
			What to expect in terms of pain during exercise	2.2b
	PT2.3	discuss goal setting and ensure timely and realistic ones are set	Setting time-based and realistic goals	2.3

	PT2.4	change exercises regularly and avoid giving too many	Regularly changing exercises	2.4a
			Avoid giving too many exercises	2.4b
	PT2.5	emphasise the need for patience with the programme- it takes time	Emphasizing the need for patience with rehabilitation programme	2.5
3	PT3	Patients understood role of exercise in reducing pain	Role of exercise in pain relief	3
4	PT4.1	Patient understands the shoulder and evidence based research	Understanding of shoulder pain	4.1a
			Understanding of evidence-based research recommendations	4.1b
	PT4.2	Patient understands pathology of shoulder	Understanding of shoulder pathology	4.2
	PT4.3	Patient sees positive results	Experiences positive outcomes of treatment	4.3
	PT4.4	Patient pain decrease	Experiences reduced pain levels in response to treatment	4.4
	PT4.5	Patient is compliant	Importance of compliance with treatment	4.5
5	PT5.1	They were given ststistical results post evidence based treatment	Given statistical results of what outcome to expect post evidence-based treatment	5.1
	PT5.2	Understood pain thresholds	Understanding of pain threshold	5.2
	PT5.3	Regular feedback and re assurance	Provided regular feedback	5.3a
			Provided regular reassurance	5.3b
	PT5.4	Clear unambiguous what will improve shoulder pain and what will exaccerbate symptoms	Clear and unambiguous information on what will improve shoulder pain and what will exacerbate symptoms	5.4
	PT5.5	An honest patient / therapist relationship	Honest patient-therapist relationship	5.5
6	PT6.1	patients were not over loaded with rehab routines.	Avoidance of overloading with rehabilitation routines	6.1
	PT6.2	Practitioners offered more education to support patient.	More education provided by practitioner to support patient.	6.2
7	PT7. 1	Better educated on self management and exercise therapy. More education on the move away from hands on, and more education on what really helps progression and decreases likelihood of reoccurrence	Self-management	7.1a
			Exercise therapy	7.1b
			Shift in treatment focus away from manual therapy treatment	7.1c

			What helps progression	7.1d
			Likelihood of reoccurrence of pain	7.1e
	PT7.2	More education on the pathway when someone has pain. Tendency to always go to GP who then refers for MRI and then to consultant who can generally recommend surgery before a realistic attempt at physio	Recommended treatment pathway to facilitate realistic attempt at physiotherapy before GP referral for imaging and surgical opinion	7.2<br>7.2
	PT7.3	Also education on the implications of surgery and the timeline of rehab after, not just a quick fix	Implications of surgery	7.3a
			Timeline of rehabilitation post-surgery	7.3b
			Emphasize surgery is not a quick-fix solution	7.3c
8	PT8.1	patients were better informed on what to expect in terms of pain when exercising. And Patients felt supported by HCPs and had a point of contact to engage with if uncertain about rehab journey. Someone to reassure them and manage expectations	What to expect in terms of pain when exercising	8.1a
			Feeling supported by healthcare providers	8.1b
			Had identified point of contact to engage with if there was any uncertainty about rehabilitation journey.	8.1c
			Provide reassurance	8.1d
			Manage patient expectations	8.1e
	PT8.2	patients/HCPs better understood normal imaging findings and their lack of association with shoulder pain	Understand lack of association between shoulder pain and imaging findings	8.2a
			Understanding of what normal imaging findings to expect	8.2b
	PT8.3	exercise interventions were more fun/engaging	Exercise interventions were more fun and engaging	8.3
	PT8.4	communication about imaging findings and best management for same was consistent across HCPs - everyone delivering the same message	Unified and consistent message from healthcare providers in relation to imaging findings	8.4a
			Unified and consistent message delivered from healthcare providers in relation to best management approach	8.4b
	PT8.5	patients had a sense of ownership over their rehab journey. Less prescriptive exercise and more choice/ability to engage in exercise that is interesting/enjoyable/possibly with social component	Sense of ownership over rehabilitation	8.5a
			Exercise that was less prescriptive	8.5b
			More choice with exercises	8.5c

			Exercises that were more interesting and enjoyable	8.5d
			Exercise with a social component	8.5e
9	PT9.1	the physio set the expectation with the patient in terms of pain when exercising, how long it might be until the patient noticed an improvement and what are the short and long term effects v surgery/injection etc.	Setting expectations in terms of pain when exercising	9.1a
			Setting expectations for how long it might be to feel noticeable improvement	9.1b
			Understanding what to expect in the short and long-term comparing exercise with surgery and/or injection	9.1c
	PT9.2	exercises were practiced in clinic (full sets and reps) during the 1st session	Practising exercises in-person (full sets and reps) during initial appointment.	9.2
	PT9.3	a follow up phone call was made to check in with the patient between appts	Follow-up phone call to check-in with patient between appointments.	9.3
10	PT10.1	HCPs would communicate the same management strategy.	Healthcare providers communicating the same management strategy.	10.1
	PT10.2	not only surgeons were considered 'specialists' in this field.	Not only **Orthopedic** surgeons were considered ‘specialists’ in this field.	10.2
	PT10.3	Popular media could inform 'our' story.	Popular media informing ‘our’ story	10.3
11	PT11.1	GPs had a better understanding of shoulder pain and evidence based treatment.	GPs had a better understanding of shoulder pain	11.1a
			GPs had a better understanding of evidence-based treatment.	11.1b
	PT11.2	f**Orthopedic** surgeons referred back to Physio more instead of doing surgery	**Orthopedic** surgeons referred back to physiotherapy more instead of opting for surgery.	11.2
12	PT12.1	Physiotherapists had specific training in how to enhance and optimise the therapeutic relationship	Physiotherapists had specific training to enhance and optimise the therapeutic relationship	12.1
	PT12.2	Physiotherapists, **Orthopedic** surgeons, and GPs had specific training in how to discuss evidence-based treatments with patients and weigh up the pros, cons, risks and benefits of the various different options (e.g. surgery, physiotherapy, injections) and ultimately provided the patient with an informed choice	Specific training for healthcare providers on how to discuss evidenced-based treatments with patients, including pros/cons/risks/benefits of treatments, to support informed choices.	12.2

	PT12.3	if there was greater collaboration and agreement between **Orthopedic** surgeons and physiotherapists in how to present evidence-based findings to patients.	Greater collaboration and agreement between **Orthopedic** surgeons and physiotherapists in how to present evidence-based findings.	12.3
	PT12.4	If first line primary care providers (GPs) had more knowledge about the lack of correlation between structural pathology on imaging and clinical symptoms.	If GPs were more knowledgeable about the lack of correlation between structural pathology and clinical symptoms.	12.4
13	PT13.1	Patients were provided with education about why exercise therapy is the best first line treatment	Why exercise therapy is the best first-line treatment	13.1
	PT13.2	Patients were given ownership over exercise programme by providing options and control over progressions/regressions	Ownership over exercise programme through giving options and control over progressions/regressions.	13.2
	PT13.3	Time was spent during the session to teach the exercises and ensure the patient is confident in performing them	Allocate time during session to teach exercises and ensure patient confidence in performing these.	13.3
	PT13.4	Patients were provided with clear written, visual, and oral information about what they should do	Provision of clear written, visual, and oral information about what patients should do	13.4
	PT13.5	Patients were given exercises that they could do quickly and easily at home with minimal or no equipment	Exercises that can be completed quickly and easily at home	13.5a
			Exercises performed at home with minimal or no equipment	13.5b
14	GP1.1	...HCPs were provided with clear guidelines, easy to read and on their desktops	HCPs given clear, easy to read guidelines	14.1a
			HCPs given access to guidelines that they could read on their desktops	14.1b
	GP1.2	...GPs were provided with a series of short concise and GP orientated educational workshops at suitable times	Short and concise GP-orientated educational workshops, ran at suitable times	14.2
	GP1.3	... a full systems campaign involving media educating the public about injuries like this, the role of exercise therapy and why MRI and surgery are not the answers	Multi-model campaign involving media to educate public about shoulder pain, the role of exercise and why MRI and surgery are not the answer.	14.3
15	PT14.1	patients were educated about pain	Pain education	15.1
	PT14.2	benefits of exercise .. making strong	Benefits of exercise, specifically strength training.	15.2
	PT14.3	time lenght of recovery	Recovery timeline	15.3
	PT14.4	scan education	Scan education	15.4
	PT14.5	outcomes better than surgery down the line	Long-term outcomes of physiotherapy better than surgery	15.5

16	PT15.1	coherent message delivered to patient from all involved	Coherent message delivered from all involved	16.1
	PT15.2	Patient aware in the absence of signifiant trauma, often findings in imaging morphological changes and most likely not the reason for your symptoms	Awareness that in the absence of significant trauma, often morphological changes on imaging not the reason for symptoms	16.2
	PT15.3	Realistic expectation setting - minium of 4 months of consistent exercise	Setting realistic expectations for exercise (minimum 4 months consistent adherence)	16.3
	PT15.4	Pt aware if shoulder symptoms are not caused by serious trauma, research shows an exercise program, together with addressing lifestyle factors, as effective as surgery	Awareness of research evidence supporting exercise combined with addressing lifestyle factors as effective as surgery for atraumatic shoulder pain	16.4
	PT15.5	Shared descision making, patient getting input on management plan & implementation of management plan eg. if exercise, whats realisitic ? x 2 week	Shared decision-making in relation to management plan and its implementation (e.g., if exercise therapy– what's realistic in terms of frequency?)	16.5
17	GP2.1	appointment with physio or similar is timely (NHS referrals in UK can take 3+ months)	Timely appointment with physiotherapy or similar	17.1
	GP2.2	realistic view of outcomes is provided	Realistic view of treatment outcomes	17.2
	GP2.3	simple schedule of exercises is provided	Provision of simple exercise schedule	17.3
	GP2.4	follow up by professional during course of treatment	Provided with follow-up during course of treatment	17.4
	GP2.5	realistic information about lack of benefit of surgery is provided	Lack of benefit associated with surgery	17.5
18	PT16.1	HCP had better understanding of neurophysiology of pain	Healthcare providers improved understanding of neurophysiology of pain	18.1
	PT16.2	HCP had better understanding of shear volume of evidence supporting exercise therapy	Healthcare providers had better understanding of mounting research evidence supporting exercise therapy	18.2
	PT16.3	Patients had cohesive experience with advice from ALL HCP (including GP/consultants) regarding lack of effectiveness of surgery	Cohesive advice from all healthcare providers	18.3a
			All HCPs provided advice regarding the lack of effectiveness of surgery	18.3b
	PT16.4	Adequate pain management options could be provided immediately	Immediate provision of adequate pain management options	18.4
19	PT17.1	HCP were fully informed about current evidence, clinical practice guidelines and recommended care pathways	Healthcare providers informed about current evidence, clinical practice guidelines and recommended care pathways	19.1

	PT17.2	HCP took the time to explain current evidence to consumers and engage in SDM for their care	HCPs took their time to explain current evidence	19.2a
			HCPs engaged in shared decision-making	19.2b
	PT17.3	HCP provided clear management plan so consumers are aware of different management options	Provision of clear management plan	19.3a
			Awareness of different management options	19.3b
	PT17.4	Consumers had access to evidence based information in simple plain language	Consumer access to evidence-based information	19.4a
			Evidence-based information in simple plain language for consumers	19.4b
20	PT18.1	Patients were informed fully on timescales and what to expect with recovery, normal to plateau at times and can be uncomfortable doing some activities for longer periods.	Fully informed on recovery timescales	20.1a
			Recovery expectations (i.e., normal to plateau at times, some activities may be uncomfortable for longer periods)	20.1b
	PT18.2	Important to know surgery is not a “quick fix”	Surgery is not a “quick fix”	20.2
	PT18.3	Surgeons on board with the same message	Surgeons on board with the same message	20.3
	PT18.4	Rehab to continue to late stages and advice throughout	Rehabilitation to continue to late stages of recovery	20.4a
			Advice provided throughout rehabilitation at each stage	20.4b
21	PT19.1	Expectations of physiotherapy and exercise therapy were communicated to the patient from the very first point of primary care contact	First point of primary care contact setting patient expectations for physiotherapy and exercise therapy	21
22	OC1.1	Patients were aware of the substantial role non-operative therapy has in treating shoulder pathology	Awareness of substantial role of non-operative therapy	22.1
	OC1.2	Patients and health care professionals were aware of the timescale involved in recovery of function	HCPs and patients aware of timescales for functional recovery	22.2
	OC1.3	Patients and healthcare professional understand that Imaging findings are not the only factor involved in management decisions	Patients and HCPs understanding that imaging findings are not the only factor involved in management decisions	22.3
23	C1.1	Bettrr unformed on why thery were doing an exercise in a particular way(eg isometrics)	Better information provided to explain rationale for type of exercise prescribed	23.1
	C1.2		Understanding that pain does not equal harm	23.2a

		If they understood hurt does not equal harm and to use shoulder to tolerance	Use shoulder as much as can tolerate	23.2b
	C1.3	Rest not necessarily helpful	Rest is not necessarily helpful	23.3
24	PT20.1	The treatments showed some early signs of general shoulder improvement in range or pain	Treatment that demonstrated early signs of improvement in range of motion or pain	24.1
	PT20.2	If the patients were shown easily understood grafics from evidence base research	Easy to understand infographics from evidence-based research	24.2
	PT20.3	Patients understood their condition, but sometimes the condition might not be so easy to diagnose	Understanding of their condition	24.3a
			Understanding of the difficulties in making a diagnosis	24.3b
	PT20.4	If the treatments are of short nature when performing daily with no more than 3 exercises in hep	Treatments that are quick and could be performed daily (e.g., maximum 3 home exercises)	24.4
	PT20.5	They feel better after they perform the hep and start to see slow gaons	Feel improvement post-exercise and see slow gains/improvement	24.5
25	PT21.1	patients were informed regarding lifestyle factors and their influences	Lifestyle factors and their influences	25.1
	PT21.2	patients were informed of the approximate (lengthy) time scale for resolution	Information regarding approximate lengthy timeline to symptom resolution	25.2
	PT21.3	patients informed regarding lack of correlation with imaging and outcomes	Lack of correlation between imaging and outcomes	25.3
	PT21.4	HCP considered finding the driver for the problem rather than concentrating on the pathological tissue	HCPs focus on identifying the driver for the problem rather than the pathological tissue	25.4
	PT21.5	HCPs enesured that patients understood explanation and how to accurately carry out exercises	Checking patient understanding of explanation given	25.5a
			Understanding of how to accurately carry out exercises	25.5b
26	PT22.1	Exercise were specific to a person's ADLs i.e. reaching for top shelf, rather than simply completing lateral raises	Individualised exercises specific to ADLs	26.1
	PT22.2	Patients were informed that pain does not mean damage. Some exercises will be more painful and some days they will experience more pain and this is a normal part of rehab	Pain does not mean damage	26.2a
			Fluctuations in pain levels day-to-day and between different exercises is normal part of rehabilitation	26.2b

	PT22.3	There is a strong therapeutic relationship in which the client feels their gears and concerns have been listened to and addressed	Strong therapeutic relationship in which patient feels their fears and concerns have been listened to and addressed	26.3
	PT22.4	Exercise volume is relatively low so as to not become tedious to complete but effective	Low volume of exercise to ensure effectiveness without making adherence tedious	26.4
27	PM1.1	patients had appropriate information prior to engaging with treatment and throughtout the tretment programme	Appropriate information provided prior to and throughout treatment programme	27.1
	PM1.2	patients reveive regular HSCP input throughtout their programme on an interval basis either virtual or FTF	Regular input from their HCP throughout treatment programme on an interval basis (virtually or F2F)	27.2
28	PT23.1	patients understood the relationship of the treatment to their goals	Understood relationship between treatment and their goals	28.1
	PT23.2	patients were educated on the efficacy of the treatment provided	Efficacy of treatment provided	28.2
	PT23.3	patients were educated on pain science	Pain science	28.3
	PT23.4	providers make an effort to and have a good relationship with their patient	HCPs make effort to have a good relationship with their patient	28.4
29	GP3.1	Patients understood their diagnosis fully	Full understanding of diagnosis	29.1
	GP3.2	Patients had a good relationship with their health care provider	Good relationship with their healthcare provider	29.2
	GP3.3	Patients had realistic goals	Realistic goals	29.3
	GP3.4	Patients had the time and resources to practice the exercises	Time and resources to practice exercises	29.4
	GP3.5	Patients had adequate pain relief so that exercises were as comfortable as possible	Adequate pain relief to allow exercises to be as comfortable as possible	29.5
30	PT24.1	if physiotherapists were better informed	If Physiotherapists were better informed	30.1
	PT24.2	if physiotherapists were better payed	If Physiotherapists were better paid	30.2
	PT24.3	if physiotherapists could spent more time with the patient	If Physiotherapists could spend more time with the patient	30.3
31	PT25.1	patients were informed consistently by different HCPs.	Consistent information provided by different HCPs	31.1
	PT25.2	Barriers to second line treatment or consult was triaged better.	More of a barrier to second-line treatment options	31.2a

			Better triaging for second-line treatment	31.2b
	PT25.3	HCPs were educated about clinically applicable EBP.	HCPs educated on clinically applicable evidence-based practice.	31.3
	PT25.4	(shoulder) health care policy was more aligned with current best evidence.	Health care policy relating to shoulder pain more aligned with current best evidence.	31.4
32	PT26.1	Patients were better informed in what to expect in terms of pain when exercising	Better information provided to manage expectations relating to pain while exercising	32.1
	PT26.2	Exercises were simplified and quick and easy to do	More simplified, quick, and easy-to-do exercises	32.2
	PT26.3	Evidence based guidelines were easily accessible and agreed	Evidence-based guidelines were easily accessible	32.3a
			Evidence-based guidelines that were agreed	32.3b
33	PT27.1	Patients understand global and specific benefits of exercising	Understanding of global and specific benefits of exercise	33.1
	PT27.2	Patients understand age related changes and accept them as parte of life	Understanding of age-related changes and accept these are normal part of life	33.2
	PT27.3	Patients know that we can't be asymtomatic for the whole life	Understanding can’t be asymptomatic all their life	33.3
	PT27.4	That our body needs time to heal and or adapt	Body needs time to heal and/or adapt	33.4
	PT27.5	Patients accept that psychological factors influence outcomes	Acceptance that psychological factors influence outcomes	33.5
34	PT28.1	Patients were educated at their initial consultation re. diagnosis, predicted recovery time, role of analgesia, role of physiotherapy and exercise, role of imaging and what's required for their injury	Early education at initial consultation regarding diagnosis	34.1a
			Early education at initial consultation regarding predicted recovery timeline	34.1b
			Early education at initial consultation regarding the role of analgesia	34.1c
			Early education at initial consultation regarding the role of physiotherapy and exercise	34.1d
			Early education at initial consultation regarding role of imaging	34.1e
			Early education at initial consultation on what is required for their injury	34.1f

	PT28.2	There is good communication between the MDT e.g GP or consultant, physiotherapist and patient with 2 way channels of communication between all members as appropriate	Good communication between all involved HCPs and patient as appropriate	34.2
	PT28.3	Patient understands their diagnosis and can discuss it, have their fears and questions addressed, question the available treatment routes, feel like they're participating in their recovery process. Can understand the psychosocial factors that can influence shoulder pain	Understanding of diagnosis and can discuss it	34.3a
			Address patient fears and questions	34.3b
			Question available treatment routes	34.3c
			Feel they are participating in their recovery process	34.3d
			Understanding of the psychological factors that can influence their pain	34.3e
	PT28.4	Patients can see and understand their progress, realistic short term goals around pain management, sleep, function	Able to understand and see progress	34.4a
			Realistic short-term goals relating to pain management	34.4b
			Realistic short-term goals relating to sleep	34.4c
			Realistic short-term goals relating to function	34.4d
	PT28.5	There is feedback and communication to referrers re. outcome for patients. Often GPs only hear about the patients who don't do well or require further intervention, not enough about the patients who make a good recovery.	Better feedback and communication with referrers irrespective of outcome (i.e., Good, or bad recovery in response to treatment)	34.5
35	PT29.1	patients weren't unneccessarily referred for imaging	Reduced unnecessary referral for imaging	35.1
	PT29.2	patients were reassured about the benefits of exercise at first contact e.g.	First contact practitioner reassurance about the benefits of about the benefits of exercise	35.2
	PT29.3	waiting lists weren't so long that patients are disheartened and frustrated when they eventually seen	Reduced length of waiting lists to avoid patients being disheartened and frustrated by the time they are seen	35.3
	PT29.4	patients were educated regarding anatomy and diagnosis, and benefits of exercise	Education regarding anatomy	35.4a
			Education regarding diagnosis	35.4b
			Education regarding the benefits of exercise	35.4c
36	PT30.1	Patients recognise that pain does not equal damage.	Pain does not equal damage	36.1
	PT30.2	A clicking joint can be a good thing.	A clicking joint can be a good thing	36.2

	PT30.3	Patients recognise that, in many cases, regardless of the outcome of the MRI, US, X-ray, we need to get the shoulder moving and start using it again.	Knowledge that irrespective of outcome of imaging, will need to get the shoulder moving and start using it again.	36.3
	PT30.4	We want patients to feel stretches/exercises, but they should be tolerable.	Prescribing exercises and stretching that patients can feel but should be tolerable	36.4
	PT30.5	Patients recognise that we may not achieve full range make the pain go away completely, but we can significantly improve function and pain management.	Recognition that the outcome of physiotherapy may not achieve full resolution of pain and range of movement, but it can significantly improve function and help manage pain.	36.5
37	PT31.1	.......patients had early access to first line treatments	Early access to first-line treatment	37.1
	PT31.2	......there was a team-based approach to managing care	Team-based approach to managing care	37.2
	PT31.3	......they had a better understanding of shoulder pathology	Better understanding of shoulder pathology	37.3
	PT31.4	.....they had access to evidence-based and standardised information regarding managing shoulder pain	Access to evidence-based standardised information regarding managing shoulder pain	37.4
	PT31.5	...we took time to educate our patients regarding interpretation of any imaging/investigations they've had done	Taking time to educate patients regarding imaging findings or other investigations they've had done	37.5
38	PT32.1	Patients were aware of evidence & believe it will work	Awareness of evidence	38.1a
			Patient belief that evidence-based treatment will work	38.1b
	PT32.2	Were given aids to help e.g apps, resources, diaries	Provision of aids to help e.g., apps, resources, diaries	38.2
	PT32.3	Had advice on how to manage pain levels	Advice on how to manage pain levels	38.3
	PT32.4	Have high self-efficacy	High self-efficacy	38.4
	PT32.5	Are not dealing with other joint issues comorbidities	Not dealing with other joint issues and comorbidities	38.5
39	PT33.1	Patients had less rather than more to complete	Patients had less rather than more time to complete	39.1
	PT33.2	Instructions were simple	Simple instructions	39.2
	PT33.3	Education around what to expect is given rather than just information on the exercise	What to expect in response to exercise rather than solely information on the exercise	39.3
	PT33.4	It is built into their specific day & demands	Exercises built into specific day and demands of the patient	39.4

40	PT34.1	Patients understood that exercise is an effective management strategy	Understanding that exercise is an effective management strategy	40.1
	PT34.2	Patients understood that they will recover well with a suitable exercise programme	Understand to expect a good recovery if following a suitable exercise programme	40.2
	PT34.3	That it is acceptable to have some pain while exercsing but that this is neither harmful nor something to be anxious about	Some pain while exercising is acceptable	40.3a
			Pain during exercise is neither harmful of something to be anxious about	40.3b
	PT34.4	Patients understood that imagin is not necessary for successful management of their shoulder problem	Imaging is not necessary for the successful management of shoulder pain	40.4
	PT34.5	That adverse findings on imaging do not equate to symptoms nor correlate with time to recovery	Adverse findings on imaging do not equate to symptoms	40.5a
			Adverse findings on imaging do not correlate with time to recovery	40.5b
41	PT35.1	Informationen about the time to recovery and pain during this period	Information on time to recovery	41.1a
			Information on pain during the recovery period	41.1b
	PT35.2	Remind the patient to do exercises (mobile app)	Reminder to do exercises (e.g., app)	41.2
	PT35.3	Inform that the Operation isn't a quick solution!	Surgery is not a quick solution	41.3
	PT35.4	Control of compliance (e.g videocall). Don't leave them alone	Provide support to help control compliance and avoid leaving patients alone (e.g., videocall)	41.4
42	PT36.1	treatment was provided in a timely fashion	Provision of treatment in a timely fashion	42.1
	PT36.2	patient's had a broader understanding on the role of physiotherapy	Broader understanding of the role of physiotherapy	42.2
	PT36.3	**Orthopedic** consultant's promoted the role of physiotherapy and conservative management	**Orthopedic** consultants promoted the role of physiotherapy and conservative management	42.3
	PT36.4	improved patient education around the role of injections and surgery - not just a quick fix approach	Improved education around the role of injections and surgery	42.4a
			Improved education that injection and surgery do not provide a quick fix approach	42.4b
	PT36.5		Improved education on the role of exercise	42.5a

		Improved education surrounding the role of exercise and the timeframes involved in rehab	Improved education on the timeframes involved in rehabilitation	42.5b
43	PT37.1	there was a greater emphasis on patient education as part of the rehabilitation programme (i.e. group information)	Greater emphasis on patient education as part of rehabilitation programme (i.e., group information)	43.1
	PT37.2	All healthcare professionals were consistent in their messaging to patients regarding pain and lack of correlation with structural pathology on MRI scans, etc.	HCPs were consistent in their messaging to patients	43.2a
			HCPs educated patients regarding pain and the lack of correlation with structural pathology on MRI scans	43.2b
44	PT38.1	The same message re its benefits were coming from all healthcare providers caring for patient. IE GP ortho doc and physio	Same message from all HCPs caring for patient	44.1a
			All HCPs provided education regarding the benefits of exercise therapy	44.1b
	PT38.2	The emphasis throughout the HC system was on active treatment and management rather than on investigation particularly for primary care	Emphasis throughout the healthcare system on active treatment and management rather than on investigation within primary care	44.2
	PT38.3	Patients and physios better understood the reason behind exeryand how to increase and reduce dosage based on patient response and how to manage flare ups	Better understanding amongst physiotherapists in relation to exercise i.e., how to modify dosage based on patient response	44.3a
			Better understanding amongst patients in relation to exercise i.e. manage flare-ups etc	44.3b
45	OC2.1	Patients understand that improvement may take several weeks	Understanding that improvement may take several weeks	45.1
	OC2.2	Patients understand other treatment options which are available in addition to therapy, e.g. injections, surgery	Understanding of other treatment options available in addition to physiotherapy, such as injections and surgery	45.2
	OC2.3	Understand the nature of the shoulder problem (impingement, rotator cuff tear, arthritis)	Understand the nature of the shoulder pain (i.e., impingement, rotator cuff tear, arthritis)	45.3
	OC2.4	Realise that many other patients have similar symptoms	Understanding that many other patients have similar symptoms	45.4
46	PT39.1	GPs were better informed about the need for rehabilitation prior to more invasive treatments, and prioritised explaining this to patients	GPs better informed about the need for rehabilitation prior to more invasive treatments	46.1a
			GPs prioritised explaining to patients they will need to engage in rehabilitation before considering invasive treatments	46.1b

	PT39.2	We had a national shoulder advice booklet or website, which all physios, GPS and surgeon referred to, so that we are delivering a consistent message	Consistent message delivered from all HCPs	46.2a
			All HCPs using national shoulder advice booklet or website to refer to	46.2b
	PT39.3	Rehabilitation was better funded or compensated to patients: many/most health insurers cover 4 or 6 physio session per year, or fewer, while public patients wait months for a primary care Physiotherapy appointment	Better funding or compensation for patients to engage in rehabilitation irrespective of private or public healthcare (e.g., private health insurers often cover 4-6 physiotherapy sessions, while those waiting on primary care physiotherapy are waiting months)	46.3
47	PT410.1	GPs had a better understanding of the evidence behind shoulder pain rather than referrig everyone for injections	GPs better understanding of the evidence behind shoulder pain rather than referring everyone for injections	47.1
	PT40.2	There was more public knowledge of managing shoulder pain.	More public knowledge of managing shoulder pain	47.2
	PT40.3	Waiting lists were not so long in primary care	Reduced length of waiting lists in primary care	47.3
	PT40.4	There was more specific shoulder classes for patients to attend	More specific shoulder classes for patients to attend	47.4
48	PT41.1	patients got written information on their problem	Written information on their shoulder problem	48.1
	PT41.2	patients got consistent information from all heathcare providers	Consistent information from all healthcare providers	48.2
	PT41.3	treatment regimens were short and easy to follow	Short and easy to follow treatment regimens	48.3
	PT41.4	they had regular reassessment with exercise prescribers	Regular re-assessment with exercise prescribers	48.4
49	PT42.1	Patients understood imaging	Understanding of imaging findings	49.1
	PT42.2	Patients had access to group supervised exercise	Access to group supervised exercise classes	49.2
	PT42.3	Patients understood pain	Understanding of pain	49.3
	PT42.4	Patients understood duration of recovery	Understanding of duration of recovery	49.4
	PT42.5	Patients understood motivation required	Understanding of motivation required	49.5
50	PT43.1	Patients were informed about exercise therapy. And pain relief	Informed about exercise therapy	50.1a
			Informed about pain relief	50.1b
	PT43.2	Patient preferred steroid injections	Patient preferred steroid injections	50.2

	PT43.3	Pt will opt for surgery rarely as far as they can manage basic tasks and pain not severe	Provided patient can manage basic tasks and pain is not severe, patients will rarely opt for surgery	50.3
51	PT44.1	if there was better understanding in general practice of the benefits of exercise for the treatment of shoulder pain and not automatically referring for surgery dependent wholly on imaging	GPs had a better understanding of the benefits of exercise	51.1a
			GPs did not automatically refer for surgery based wholly on imaging findings	51.1b
	PT44.2	if patients were given realistic time guidelines for resolution of pain either with or without surgical intervention	Provision of realistic time guidelines for resolution of pain with or without surgical intervention	51.2
	PT44.3	if the benefits of nonsurgical intervention were promoted more in general practice and consultant clinics	Promotion of the benefits of non-surgical intervention in GP and consultant clinics	51.3
	PT44.4	that imaging should only be used when absolutely neccessary where there is no improvement with physiotherapy	Imaging should only be used if necessary where there has been no improvement with physiotherapy	51.4
	PT44.5	a standardised protocol that can be followed by patient and practitioner where outcome measures are clear and realistic	Standardised protocol that can be followed by both patients and HCPs	51.5a
			Clear and realistic outcome measures	51.5b
52	PT45.1	patients expectations were managed better in regards to first line treatments and timelines of same	Managing expectations in relation to first-line treatments	52.1a
			Managing expectations in relation to timelines of first-line treatment	52.1b
	PT45.2	**Orthopedic** surgeons/other medics were more cohesive with advice given by PT	Advice given by **Orthopedic** surgeons/other medics more cohesive with advice given by Physiotherapist	52.2
	PT45.3	if clinicians were more confident in approaching their treatment ie. better understanding of shoulder complex	Clinicians more confident in approaching their treatment through having a better understanding of the shoulder complex	52.3
	PT45.4	if patient expectations in regards to surgery were managed better	Managing expectations regarding surgery	52.4
	PT45.5	If type of exercise given was more inline with patients interests	Type of exercise given more in-line with patient interests	52.5
53	PM2.1	patients were fully informed before commencing the programme	Patients fully informed prior to commencing physiotherapy programme	53.1
	PM2.2	patients could access information throughout their programme eg webdased info from the programme provider/ programme	Access to information for patients throughout their programme from the provider e.g., web-based information	53.2

	PM2.3	patients were to have regular virtual contact from their clinician to maintain motivation and maintain correct info input	Regular virtual contact from treating clinician to maintain motivation and maintain correct information input	53.3
54	PT46.1	Understood the causes of their pain more	Understanding of the causes of their pain	54.1
	PT46.2	That not all rehab ex's pain is bad pain	That not all rehabilitation exercise-induced pain is bad pain	54.2
	PT46.3	The basic healing stages and time frames	The basic healing stages and time frames	54.3
	PT46.4	That change / healing takes time	That change and healing takes time	54.4
55	PT47.1	Patients were informed of the strong evidence to support exercise based therapy	Information on the strong evidence to support exercise-based therapy	55.1
	PT47.2	Clinicians give examples of previous clients who improved with exercise	Examples given by clinician of previous clients who improved with exercise	55.2
	PT47.3	The clinician uses motivational interviewing skills to find put whats important to the client and how to help them achieve their goals	Motivational interviewing skills to find out what is important to the clinician and how to help them achieve their goals	55.3
	PT47.4	The clinician explains the rationale of the exercises and why hands on therapy alone may not have a good ooutcome	Explanation for the rationale of the exercises	55.4a
			Explanation for why hands on therapy alone may not have a good outcome	55.4b
	PT47.5	If the clinician listens carefully to what the client wants to achieve	Clinician listens carefully to what the client wants to achieve	55.5
56	PT48.1	Patients understood why their shoulder symptoms took so long to improve	Understanding of why symptoms take so long to improve	56.1
	PT48.2	patients understood the various factors that caused their shoulder pain to occur	Understanding of various factors that caused the occurrence of their pain	56.2
	PT48.3	patients understood why they have to manage their activity levels based on their pain	Understanding of why activity levels must be managed based on their pain	56.3
57	PT49	the therapist understands the goals of the patient	Therapist understands the patients' goals	57.1

58	PT50.1	If patients understood why much of shoulder pain can be classified as non-specific	Understanding why much of shoulder pain can be classified as non-specific	58.1
	PT50.2	If patients were given a potential timeline for how long rehab would take	Given potential timeline for how long rehabilitation would take	58.2
	PT50.3	If HCPs did better understand the results of imaging	HCPs were better able to help patients understand the results of imaging	58.3
59	PT51.1	Patient education about the pathology and prognosis	Education about the shoulder pathology	59.1a
			Education about the prognosis	59.1b
	PT51.2	Knowledge about rehabilitation process and expectation	Knowledge about rehabilitation process and what to expect	59.2
	PT51.3	Need for exercise adherence, reason for the specific exercises	Need for exercise adherence	59.3a
			Rationale for specific exercises	59.3b
	PT51.4	Activity modifications required in short or long term	Information relating to activity modification in the short and long-term	59.4
60	PT52.1	They had reminders or an app to track and remind them of their program	Reminders or APP to track exercise and remind them of their programme	60.1
	PT52.2	To understand how muscle tissue builds and timescales	Understanding of how muscle tissue builds	60.2a
			Understanding of timescale for building up muscles	60.2b
	PT52.3	To understand how pain influences muscle performance and how to pace	Understanding of how pain influences muscle performance	60.3a
			Understanding of how to pace	60.3b
	PT52.4	Facts and figures regarding physio vs injection vs surgery	Provision of facts and figures comparing treatment options (e.g., Physiotherapy Vs Injection Vs Surgery)	60.4
	PT52.5	Facts on imaging and what normal findings are	Facts regarding imaging and what normal findings would be	60.5
61	PT53.1	Shoulder Pain and Rotator Cuff injury is normal	Knowledge that shoulder pain and rotator cuff injury is normal	61.1
	PT53.2	1 in every 2 will likely experience shoulder pain relating to rotator cuff tear at some point in their lives	Knowledge that 1 in 2 people will experience shoulder pain relating to rotator cuff tear at some point in their lives	61.2
	PT53.3		MRI findings do not always correlate with the source of pain	61.3a

		MRI findings do not always correlate with the source of pain and pain generally decreases as strength and function improves	Pain generally decreases as strength and function improves	61.3b
	PT53.4	Pain with exercise is safe once within a 5 out of 10 NRS and does not get worse after exercise	Pain while exercising is safe provided the intensity does not exceed 5/10	61.4a
			Pain while exercising is safe provided pain does not get worse after exercise	61.4b
	PT53.5	Long-term outcomes after surgery are often worse than compared with conservative management.	Long-term outcomes following surgery are often worse than compared to conservative management.	61.5
62	PT54.1	Patients were aware of the typical timeline you would expect to follow to see improvements	Awareness of typical timelines to expect to see improvements	62.1
	PT54.2	Patients were educated on the short-term benefits of manual therapies and that exercise therapy shows more long term results	Educated on short-term benefits of manual therapies	62.2a
			Educated that exercise therapy shows more long-term results	62.2b
	PT54.3	If other HCPs were aware of the first line treatment options	If other HCPs (as well as Physiotherapists) were aware of first-line treatment options	62.3
	PT54.4	Imaging is limited to cases that do not resolve after first line rx or if red flags are present	Imaging only appropriate for cases that do not resolve after first-line treatment or if red flags are present	62.4
63	PT55.1	As per example statement 1 above	Patients were better informed on what to expect in terms of pain when exercising	63.1
	PT55.2	As per example statement 2 above	Patients and HCPs better understood normal imaging findings and their lack of association with shoulder pain	63.2
	PT55.3	Patients/HCPs understood nature, behaviour shoulder MSK presentations, incl. sinister path/disease rare, flare-ups normal (not sinister), management key, no 'fix'.	Patients and HCPs understood nature of shoulder pain	63.3a
			Patients and HCPs understood behaviour of musculoskeletal shoulder pain presentations	63.3b
			Patients and HCPs understood that sinister pathologies or diseases were rare	63.3c
			Patients and HCPs understood flare-ups are normal and do not indicate something sinister	63.3d
			Patients and HCPs understood management is key	63.3e
			Patients and HCPs understood there is no ‘fix’ available	63.3f

	PT55.4	Patients/HCP received approppain education.	Patients and HCPs received appropriate education	63.4
	PT55.5	Management included exposure value based activities.	Management included exposure to value-based activities	63.5
64	PT56	patients were informed on the limit of their exercise in discomfort terms and to be given permission to move their shoulder to that range of movement	Patient awareness of the limit of their exercise in discomfort terms and given permission to move their shoulder to that range of movement	64
65	PT57	Patients have a clear understanding of their issue, what structure is involved, the mechanism of injury , relevance of radiology and what treatment most recent evidence supoorts	Clear understanding of their issue	65a
			Clear understanding of what structure is involved	65b
			Clear understanding of the mechanism of injury	65c
			Clear understanding of the relevance of radiology	65d
			Clear understanding of what treatment most recent research evidence supports	65e
66	PT58	It was explained to patients that it may take up to six weeks to feel the benefit of exercise for their shoulder.e	Explanation that it may take up to six weeks to feel the benefit of exercise	66
67	PT59.1	patients made decisions on their treatment plan early in their care which should be a joint plan between HCP and patient..	Joint decision-making about treatment early in care between HCP and patient	67.1
	PT59.2	patients and HCP's understood normal imaging findings and their lack of association with shoulder pain. They should have their scan explained to them in language they understand by their treating clinician, they should not routinely be sent for scans unless clear indication.	Patients and HCPs understood normal imaging findings and their lack of association with shoulder pain	67.2a
			Provision of explanation of scan provided to patient in understandable language	67.2b
			Patients should not be sent for routine scans unless there is a clear indication	67.2c
	PT59.3	more time is spent on education of their condition and the benefits, commitment required and process of completing an exercise programme.	More time spent providing education of their condition	67.3a
			Education on the benefits of an exercise programme	67.3b
			Education on the commitment required and process involved in completing an exercise programme	67.3c
	PT59.4	more time is spent on discussing other contributing factors to shoulder pain e.g. stress and anxiety, obesity, smoking	More time spent on discussing other contributing factors to shoulder pain e.g. stress, anxiety, obesity, and smoking	67.4
	PT59.5		More time spent discussing realistic goals	67.5a

		more time is spent on discussing realistic goals and often acceptance that pain may not always go away but focus of treatment is on function.	More time spent supporting acceptance that pain may not always go away but focus of treatment is on function	67.5b
68	PT60.1	Patients were better informed of good outcomes following evidence based approach to management	Information on good outcomes following evidence-based management approaches	68.1
	PT60.2	HCPs carry out more specific researches and Randomised trials in order to determine the best approsch to management	HCPs conduct more research (e.g., RCTs) to determine the best approach to management	68.2
	PT60.3	More workshops and seminars	More educational workshops and seminars	68.3
	PT60.4	Funding for researchers	More financial funding for researchers	68.4
	PT60.5	Working together of all HCPs	All HCPs working together	68.5
69	PT61	The public were better inforned on the importance of helping themselves to recover rather than a passive attitude to treatment	Public better informed as to the importance of their role in being actively involved in their recovery rather than having a passive attitude to treatment	69
70	PT63.1	Explain how it will help and why	How the treatment will help and why	70.1
	PT62.2	Explain what response to expect and when	Expectations of what response to expect from treatment and when to expect this	70.2
	PT62.3	Advise for managing a flare up/set back	Managing a flare-up of symptoms or a set-back in their recovery	70.3
	PT62.4	Yes explain imaging findings and relevance to them	Explaining imaging findings and relevance to the person	70.4
	PT62.5	Fit the ideal schedule with what they can commit to	Adapting the ideal exercise schedule with what they can commit to	70.5
71	PT63.1	Patients were educated in the benefits of posture and lifestyle and how they effect muscle function	Education on the benefits of good posture and a healthy lifestyle and the effect this has on muscle function	71.1
	PT63.2	Patients understood aim pathways	Understanding of aim of management pathways	71.2
	PT63.3	Patients were educated about stress response and fear avoidance	Educated about the stress response	71.3a
			Educated about fear avoidance	71.3b
72	PT64.1	that we explain that exercise is necessary for recovery regardless of surgery or not	Explain that exercise is necessary for recovery regardless of whether surgery is required or not	72.1
	PT64.2	that exercising properly over time can yield quicker results than surgery	Exercise completed properly over time can yield quicker results compared to surgery	72.2

	PT64.3	that we select properly, candidates suitable for exercise therapy	Proper selection of candidates suitable for exercise therapy	72.3
	PT64.4	that we assess properly the patients understanding of the condition	Proper assessment of patient understanding of their condition	72.4
73	PT65.1	Patients could self refer to their local health service and be seen by a physiotherapist as a first line treatment when they first present with shoulder pain	Option to self-refer to local health service to access first-line treatment from Physiotherapy	73.1
	PT65.2	GP's were aware of the evidence eg of physios or the new Triage Clinical specialist physios could educate GPs on MSK physio management of shoulder pain	Improved GP awareness of research evidence for physiotherapy	73.2a
			Education for GPs on musculoskeletal management of shoulder pain by clinical specialist physiotherapists working in Triage services.	73.2b
	PT65.3	GPs often refer patients to **Orthopedic**s, patients wait to see doctor. Doctor may or may not have knowledge of first line treatment best practise so if the ortho doctors had knowledge it would help so that we are all singing from same hymn sheet	For GPs and **Orthopedic** consultants to have knowledge of best-practice first line treatment options so all HCPs singing from same hymn sheet	73.3
	PT65.4	Waiting times improved and there was less chronicity. While patients are on waiting list they could attend a one off info session on shoulder pain and rehab before they see 1:1 physio and this might give them a chance to take on some info in advance of seeing physio	Improved waiting times to reduce risk of chronicity of symptoms	73.4a
			If patients on waiting list had option to attend once-off information session on shoulder pain and rehabilitation before initial appointment with physiotherapy to allow time to take in information given	73.4b
74	PT66.1	... HCPs take time to educate patients about likely course, treatment options and time frame	HCPs take time to educate on likely course of treatment	74.1a
			HCPs take time to educate on treatment options	74.1b
			HCPs take time to educate on time frames	74.1c
	PT66.2	HCPs ensure Commuunication is ongoing re abouve and revisited often (patients forget)	Ongoing communication with topics relating to treatment options and timeframes revisited frequently to help information recall	74.2
	PT66.3	Patients are offered reasonable reassurance	Provision of reasonable level of reassurance	74.3
75	PT67.1	Tailored approach to patient	Tailored approach	75.1
	PT67.2	Startinf exercise at apropriate level for patient	Exercise started at appropriate level for patient	75.2
	PT67.3	Providing reassurance regarding prognosis	Reassurance regarding prognosis	75.3

76	PT68.1	Timelines for recovery where explained to patients (not quick fix)	Communication of recovery timelines emphasizing this is not a quick fix	76.1
	PT68.2	GP + Physio's were up to date to latest research, plus we're clear on how to implement	GPs and Physiotherapist up to date with latest research	76.2a
			GPs and Physiotherapist clear on how to implement latest research	76.2b
	PT68.3	Patients knew why they were doing x,y or z for treatment. Education is important for adherence.	Improved adherence if better educated as to the reason why doing specific elements of treatment	76.3
77	PT69.1	Patients could also get sick leave like if they had surgery	Sick leave was also provided for treatment other than surgery	77.1
	PT69.2	The main care provider wasn't a doctor	The main healthcare provider was not a doctor	77.2
	PT69.3	The rehabilitation could be delivered regularly with progress tracking and objective improvements	Regular rehabilitation with tracking of progress and objective improvements	77.3
	PT69.4	Physios were considered equal to medical doctors	Physiotherapists considered equal to medical doctors	77.4
78	PT70.1	If we had a better exercise culture in Ireland / basic knowledge of exercise. This is improving with younger people now	Better exercise culture in Ireland	78.1a
			Basic knowledge of exercise	78.1b
	PT70.2	If patients and more HCP knew many imaging finding are normal and not the cause of their pain	Greater awareness amongst HCPs that imaging findings are normal and not the cause of their pain	78.2
79	PT71.1	GP's were better informed especially when they are first point of contact	Better informed GPs given they are first point of contact	79.1
	PT71.2	all surgeons used a trusted group of specialist shoulder physios to treat more complex patients	**Orthopedic** Surgeons used trust group of specialist shoulder physiotherapists to treat more complex presentations	79.2
	PT71.3	patients had a trusted website with information and exercises that would complement the one-to-one physiotherapy session.	Trusted website for patients with information and exercises that would complement 1:1 physiotherapy sessions	79.3
80	PT72.1	We sit and listen to their whole story of their shoulder pain in a holistic manner. Empathise with them and ask them what they think is going on in their shoulder	Holistic approach and listening to the patients' whole story	80.1a
			Empathise with patient	80.1b
			Explore beliefs surrounding cause of shoulder pain	80.1c
	PT72.2	Timelines for strengthening programs for shoulders are explained on first session so you can set expectations	Setting expectations in first session relating to timelines for strengthening programmes	80.2

	PT72.3	Help patients understood that imaging of their shoulder could be the same as imaging of another person of similar age with no symptoms.	Understanding that imaging findings of symptomatic shoulder can be the same as another person of similar age with no symptoms	80.3
	PT72.4	Educate patients on the available evidence for surgery versus conservative management results for shoulder one year down the line.	Education on the available evidence comparing surgery and conservative management at one year follow-up	80.4
	PT72.5	Give patients a print out of their exercises and work alongside them to figure how they might fit it into their week. Give them choice in exercises etc. they need to be part of the process.	Provision of print out of exercises for patient	80.5a
			Working alongside patient to help them to figure out how they might fit exercises prescribed into their schedule	80.5b
			Involve patient in process of exercise prescription by providing them with a choice in what exercises they complete	80.5c
81	PT73.1	Patients were shown a model of the shoulder and it was explained to them the reason for their pain and how it occurred	Using a shoulder anatomy model to explain reason for pain and how it occurred	81.1
	PT73.2	The reason for the exercise program and the purpose and goal of each exercise	Education to explain reason for exercise programme	81.2a
			Education on the purpose and goal of each exercise	81.2b
	PT73.3	Patients knew and understood how long it could take to see results and the benefit of persevering with the exercises	Knowledge and understanding of timeline to see results with exercise	81.3a
			Knowledge and understanding of the benefits of persevering with the exercises	81.3b
	PT73.4	Patients understood and it was explained to them the reason to avoid surgery initially and the benefits of first live line treatments	Understanding of why surgery should be avoided initially	81.4a
			Understanding of the benefits of first-line treatments	81.4b
	PT73.5	Patients were followed up regularly in order to ensure they were doing the exercises correctly and to reassure them with regards to their progress	Regular follow-up to ensure exercises completed correctly	81.5a
			Provide reassurance regarding progress	81.5b
82	PT74.1	Patients were consistently told that evidence for surgery is no more effective than exercise	Consistent message delivered to patients	82.1a
			Education relating to the evidence that surgery is no more effective than exercise	82.1b
	PT74.2	Patients and clinical staff were aware that they had to exercise for 12 weeks in order to see effective gains	Patients and HCPs aware the need to exercise for 12 weeks in order to see effectiveness and visible gains	82.3

	PT74.3	Guidance on specific, evidence based exercises was provided to Physiotherapists	Guidance for Physiotherapists on specific evidence-based exercises	82.3
83	PT75.1	Well explained comittment required and time frame involved	Explanation of commitment required with exercise therapy	83.1a
			Explanation of timeframe involved with exercise therapy	83.1b
	PT75.2	Each step well explained and reference to scan results and skeleton as required	Each step of treatment explained thoroughly using scan results and skeleton models as required to help educate	83.2
84	PT76.1	...**Orthopedic** consultants encouraged patients to adhere to conservative tx	**Orthopedic** consultants encouraged patients to adhere to conservative management	84.1
	PT76.2	...physiotherapists applied a functional and kinetic chain approach to prescription	Physiotherapists applied functional and kinetic chain approach to exercise prescription	84.2
	PT76.3	...physiotherapists set achievable visible short term goals that gets buy in from patients as they see improvements	Physiotherapists set achievable visible short-term goals	84.3a
			Setting short-term goals so that patients can see improvements to help get buy-in	84.3b
85	PT77	Simple information	Simple information	85
86	PT78.1	Improved technology	Improved technology	86.1
	PT78.2	Increasing health literacy at a young age	Improved health literacy at younger age	86.2
	PT78.3	Promoting physical activity	Promotion of physical activity	86.3
87	PT79.1	Physios clear with patients re timescale for improvement	Physiotherapists clear with patients regarding expected timeframe for improvement	87.1
	PT79.2	GPs were better informed re treatment options	GPs better informed regarding treatment options	87.2
	PT79.3	all healthcare practitioners could speak confidently to patients re pros and cons of all treatment options. Treatment options are not all bad or all good	All HCPs were able to speak confidentially to patients regarding the pros and cons of all treatment options	87.3
	PT79.4	If there was international agreement regarding exercise type, dose etc appropriate for conditions	International agreement regarding exercise type and dosage for various shoulder conditions	87.4
	PT79.5	all physiotherapists kept informed re evidence based practise	All Physiotherapists kept informed regarding evidence-based practice	87.5

88	PT80.1	The patient had a good understanding of why they were doing the exercises	Good understanding of why they are doing the exercises	88.1
	PT80.2	There were not too many exercises.	Avoidance of prescribing too many exercises	88.2
	PT80.3	They strongly believed that doing the exercises will help their pain and Function	Strong patient belief in the potential of exercise to help pain and function	88.3
	PT80.4	Patients were given a management plan with timeframes, rather than just going from session to session	Provision of management plan with clear timeframes	88.4
	PT80.5	HCPs can get the message across that it will take time and effort to change the shoulder pain, and the patient will need to be consistent and patient with the advice/ exercises	Education that it will take time and effort to change shoulder pain	88.5a
			Importance of consistency and patience when adhering to advice and exercise given	88.5b
89	PT81.1	Surgeons ensured all potential patients did a 12 week rehab programme, 3 times per week, prior to be considered for surgery	**Orthopedic** surgeons stipulated any patients completed a 12-week rehabilitation programme (3 x a week) prior consideration for surgery	89.1
	PT81.2	A comprehensive plan of pain man to run in parallel with rehab	Comprehensive pain management running parallel with rehabilitation	89.2
	PT81.3	Public campaign to increase awareness of the 12 week programme	Public campaign to increase awareness of the need to complete a 12-week programme	89.3
	PT81.4	Improve GPS understanding of referring patients with shoulder pain for the programme whilst in pain meds not after they have finished the course	Improved GP understanding of importance to refer patients for physiotherapy once pain medication prescribed and not waiting until course of medication is complete	89.4
	PT81.5	Patient education that on site supervised rehab is the gold standard	Education that on-site supervised rehabilitation is the gold standard	89.5
90	PT82.1	Evidence was more readily accessible to practitioners	Research evidence more readily accessible to HCPs	90.1
	PT82.2	There was less expectation of manual therapy treatment	Less expectation of manual therapy treatment	90.2
91	PT83.1	GP's and **Orthopedic** consultants strongly encouraged conservative management at the outset.	GPs and **Orthopedic** consultants strongly encouraged conservative management at the outset.	91.1
	PT83.2	Patients had a better understanding as to the science of pain.	Understanding of pain science	91.2
	PT83.3	Patients/HCPs better understood normal imaging findings and their lack of association with shoulder pain	Patients and HCPs better understood normal imaging findings and their lack of association with pain	91.3

92	PT84.1	All health professionals were singing off the same hymn sheet as regards thr effectiveness of Conservative treatment	All HCPs were signing off the same hymn sheet as regards the effectiveness of conservative treatment	92.1
	PT84.2	Patients were better informed about the length of time it can take to see results with Conservative treatment	Better informed about timeframe to see results with conservative treatment	92.2
	PT84.3	There was better communication between hospital consultants and primary care physiotherapist	Better communication between hospital consultants and primary care physiotherapists	92.3
	PT84.4	Physiotherapists were better I formed about the type of exercise prescription that is the most effective eg type of exercise, amount of load etc	Physiotherapists were better informed about the most effective type of exercise prescription (e.g., type of exercise, amount of load etc.)	92.4
	PT84.5	High quality Group classes were available in all opd departments	Availability of high-quality group classes in outpatient departments	92.5
93	PT85.1	Patients were able to see results/improvements in their shoulder pain when they comply with exercise programmes	Able to see results/improvements in pain with compliance with exercise programmes	93.1
	PT85.2	They realised the inconsistent and uncertain outcome of surgery	Knowledge of inconsistent and uncertain outcomes following surgery	93.2
	PT85.3	They had a better understanding of the issue and reassurance regarding their 'Rotator cuff tear'.	Better understanding of their shoulder issue	93.3
			Reassurance regarding knowledge of presence of ‘rotator cuff tear’	93.4
	PT85.4	Patients didn't believe other people and their experiences	Patients didn’t believe inaccurate information based on other people’s experiences	93.5
94	PT86.1	A clear explaination is given for why the treatment is prescribef	Clear explanation given as to why treatment is prescribed	94.1
	PT86.2	If an immediate positive result is noticed	If an immediate positive result is noticed	94.2
	PT86.3	The exercises prescribed are simple and easy to follow	Simple and easy to follow exercises prescribed	94.3
95	PT87.1	patient had access to the most up-to-date evidence-bases recommendations communicated in a non-biased way	Access to the most up-to-date evidence-based recommendations communicated in a non-biased way	95.1
	PT87.2	patients had an understanding that the changes seen on imaging don’t necessarily relate to their pain	Understanding that the changes seen on imaging don’t necessarily relate to their pain	95.2
	PT87.3	patients had a clear care pathway with access to someone knowledgable relating to evidence-based management recommendations	Clear pathway with access to someone knowledgeable relating to evidence-based management recommendations	95.3

	PT87.4	patients would be more likely adhere to exercise programmes if they were more functional and relevant to their lifestyle and goals	Exercise programmes that are more functional and relevant to patient lifestyle and goals	95.4
	PT87.5	patients would be more likely to pursue conservative management if physios were acknowledged as equals amongst the HCP team and not something “to try”	Acknowledgment of physiotherapists as equals amongst the HCP team and not something “to try” to improve likelihood of patients pursuing conservative management	95.5
96	PT88.1	Better explanations of imaging findings	Better explanation of imaging findings	96.1
	PT88.2	More promotion of exercise as best treatment intervention	Promotion of exercise as best treatment intervention	96.2
	PT88.3	Greater compliance from gps and surgeons with evidence as opposed to injection surgery as first line treatment	Greater compliance from GPs and **Orthopedic** surgeons to evidence-based recommendations as opposed to injections and surgery as first line treatment	96.3
97	PT89.1	HCPs took more time educating the patient in a format that suited the patient	Educating the patient in a format that suited the patient	97.1
	PT89.2	Expectations for pain and timeframes were communicated clearly to patients	Clear communication of expectations in relation to pain and timeframes	97.2
	PT89.3	Patients were given exercises that were meaningful to them	Exercises prescribed that are more meaningful to patient	97.3
	PT89.4	Meaningful goals were created with the patient	Creation of meaningful goals with the patient	97.4
	PT89.5	Healthcare professional colleagues understood better the role of physio and did not suggest to patients non evidence based management	Better understanding amongst HCPs of the role of physiotherapy and avoidance of directing patients towards non-evidence-based management	97.5
98	PT90.1	patients were told by surgeons to do physio first for at least 3 months and be honest about surgical results	Educated by surgeons to do physiotherapy first for a minimum of 3 months	98.1a
			**Orthopedic** surgeons provide honest information about surgical results	98.1b
	PT90.2	patients could access quality physio quickly, with the emphasis on exercise and education not passive therapies	Access to quality physiotherapy quickly	98.2a
			Emphasis of physiotherapy treatment on exercise and education not passive therapies	98.2b
99	PT91.1	Physios explain in simple language why the non surgical approach is the best first line of RX (tissue healing/muscle rebuilding giving balance to jt kinetics etc)	Physiotherapists provide education in simple language	99.1a
			Why non-surgical approach is the best first-line treatment	99.1b

			When educating on the pros of non-surgical approach providing information relating to its effects on tissue healing, muscle strengthening, and symmetry of joint kinetics.	99.1c
	PT91.2	Explaining the time frame within which to expect improvement	Timelines within which to expect improvement	99.2
	PT91.3	Monitoring the pt over that time frame (whether F2F or by ph/video call) to encourage compliance	Monitoring throughout recovery (either F2F/phone/video call) to encourage compliance	99.3
	PT91.4	Being able to offer empirical examples of process with conservative management (which obviously only comes with years of clinical exposure!)	Provide empirical examples of process of conservative management	99.4
	PT91.5	explaining "normal" age related changes of MRIs/X-Rays etc	Explaining normal age-related changes seen on imaging	99.5
100	PT92.1	All HCPs were in the know of first line treatment for shoulder patients	All HCPs were knowledgeable of first-line treatment	100.1
	PT92.2	Patients to be referred to Physiotherapist with an interest in Shoulder and develop a shoulder sign on their website that would be nationally/internationally recognised	Referral to physiotherapists with a special interest in shoulder pain recognised nationally and internationally with a shoulder sign	100.2
	PT92.3	Stop prescribing all painful exercises for shoulders that we learned at college.	Cease prescription of all painful exercises for shoulders	100.3
	PT92.4	Assess and reassess the client	Continuous assessment and re-assessment of the patient	100.4
	PT92.5	Use a Corticsteroid injection urgently if signs of a frozen shoulder, dec in lat rot, abduction& medial rot.Stop putting shoulder in slings for 2 weeks after injections-advise how long support is needed.	Urgent use of corticosteroid injections if signs of frozen shoulder (reduced lateral rotation, abduction, and medial rotation)	100.5a
			Discontinue use of shoulder slings in 2-week period post-injections and advise how long support needed	100.5b
101	PT93.1	HCPs were more confident in providing education to the patients	Increased HCP confidence in educating patients	101.1
	PT93.2	Easy to access ressources for education and exercises were available for the patients	Easy access to resources containing education and exercise information available for patients	101.2

	PT93.3	HCPs were more skilled to understand the objectives and expectations of the patients	Improved HCP skills to understand patient objectives and expectations	101.3
	PT93.4	HCPs knew which treatments are evidence-based in shoulder pain management	HCP knowledge of evidence-based treatments	101.4
	PT93.5	HCPs were more skilled in indicating a diagnosis without using diagnostic imaging.	Improved skills of HCPs to indicate diagnosis without the need of imaging	101.5
102	PT94.1	people did not attend practitioners who catastrophised their pain	Avoidance of HCPs who catastrophize patient pain	102.1
	PT94.2	patients had access to supervised exercise facilitites	Access to supervised exercise facilities	102.2
	PT94.3	have built up a good relationship of trust with their therapist	Built up a good relationship of trust with treating HCP	102.3
	PT94.4	therapists could provide clinical pilates and exercise classes	Provision of clinical Pilates and exercise classes	102.4
	PT94.5	there was a good database of information available through ISCP	Good database of information available through the Irish Society of Chartered Physiotherapists (ISCP)	102.5
103	PT95.1	all healthcare providers encouraged evidenced based care in RCRSP	Encouragement of HCPs to follow evidenced-based care in rotator cuff-related shoulder pain (RCRSP)	103.1
	PT95.2	GP's didn't refer to **Orthopedic**s at the same time as physiotherapy	GPs didn't refer simultaneously to **Orthopedic**s and physiotherapy	103.2
	PT95.3	Better explanation of MRI and x-ray normal age related findings are supplied to clients	Better explanation of imaging findings and normal age-related findings	103.3
104	OC3.1	patients were aware of the burden of surgical rehab	Awareness of burden of surgical rehabilitation	104.1
	OC3.2	patients were clear on the reasons and rationale behind conservative management	Clear understanding of reasons and rationale behind conservative management	104.2
	OC3.3	patients were provided with clear, concise, understandable information at the time of clinical review and with an opportunity to review that information at a later date (eg via a website/video edication format)	Provision of clear, concise, understandable information at the time of clinical review	104.3a
			Opportunity for patient to review information provided at their initial clinical review at a later date (via website/educational video)	104.3b
	OC3.4	patients had clear testimonials (with video of pre and post Rx pain and ROM) from patients having successfully undertaken conservative management.	Clear testimonials from patients having successfully undertaken conservative management (with video of pre- and post-treatment pain and range of movement)	104.4

105	OC4.1	Patients understood the goal and likely duration of non op management	Understanding of goal of non-operative management	105.1a
			Understanding of likely duration of non-operative management	105.1b
	OC4.2	Imaging doesn't always correlate with clinical problem	Imaging findings don't always correlate with clinical problem	105.2
	OC4.3	Patients need to engage with rehab, the pain won't fix itself	the need to engage with rehabilitation as the pain won't fix itself	105.3
106	OC5.1	patients understand the natural history of atraumatic shoulder pathologies	Understanding of the natural history of atraumatic shoulder pathologies	106.1
	OC5.2	if patients understand that positive findings on imaging/exam can be physiological rather than pathological	Understanding that positive findings on imaging and examination can be physiological rather than pathological	106.2
	OC5.3	if patients have adequate pain control during non-surgical treatment	The need for adequate pain control during non-surgical treatment	106.3
	OC5.4	if patients are reassured that exercises wont damage their shoulders	Reassurance that exercises won't damage shoulder	106.4
	OC5.5	if patients understand that surgery is not a quick fix, that success is not guaranteed and complications may arise	Understanding that surgery is not a quick fix	106.5a
			Understanding that success is not guaranteed	106.5b
			Understanding that complications may arise	106.5c
107	OC6.1	Expectations and natural history explained	Setting expectations	107.1a
			Explained natural history	107.1b
	OC6.2	The rare need for surgery explained	Explanation that surgery is rarely needed	107.2
	OC6.3	Physios in intermediate care are stopped from requesting mri scans	Stopping physiotherapists in intermediate care from requesting MRI scans	107.3
	OC6.4	Physios allowed rapid access to simple shoukder xrays	Allowing physiotherapists rapid access to simple shoulder X-Rays	107.4
	OC6.5	Frozen shoukder treated with pain relief eg steroid injection and not have MRI	Treatment of frozen shoulder with pain relief (e.g., steroid injection) and not the need for MRI	107.5
108	PT96.1	Patients were given clear information about the time frames for improvement with treatment for their condition and how much pain is acceptable	Clear information about the timeframes for improvement with treatment for their condition	108.1a
			Clear information about how much pain is acceptable	108.1b

	PT96.2	Patients were given some choice in their selection of exercises	Given choice in their selection of exercises	108.2
	PT96.3	Patients were given a clear exercise prescription with high quality resources including videos, handouts etc	Given clear exercise prescription with high quality resources (incl. vidoes, handouts etc.)	108.3
	PT96.4	Patients recieved regular reminders to do their exercises, such as from an app	Regular reminders to do exercises (e.g., from an APP)	108.4
	PT96.5	Patients with poor prognosis such as low self efficacy could access more physiotherapy input if required	Patients identified as having poor prognostic risk factors such as low self-efficacy could access more physiotherapy input if required	108.5
109	GP4.1	That the patient has an understanding of the underlying nature of shoulder pains	Understanding of the underlying nature of shoulder pain	109.1
	GP4.2	That they can link this knowledge to actions that can improve their pain	Ability to link knowledge about the nature of pain to actions that can improve their pain	109.2
	GP4.3	If they are given a realistic time line for expected improvement	Given realistic timeframe for expected improvement	109.3
	GP4.4	That they have a pathway back to their healthcare provider in case there is no improvement,.	Pathway back to healthcare provider if no improvement	109.4
110	PT97	Patients had a better understanding that pain is a normal part of the human experience and that with time and understanding the strong likelihood is that it will settle	Better understanding that pain is a normal part of the human experience	110a
			Better understanding that with time and understanding there is a strong likelihood it will settle	110b
111	PT98.1	patients were better informed as to expected time frames when commencing exercise	Better informed regarding expected timeframes when they commence exercise therapy	111.1
	PT98.2	patients were told what the problem was and what needed to be done by way of exercise to help.	Patients told what the problem is and what exercise they need to do to help this.	111.2
112	PT99.1	Patients weren't over diagnosed and given reassurance that their pain is normal	Avoid over-diagnosing patients and instead provide reassurance their pain is normal	112.1
	PT99.2	An increased number of therapy assistants to carry out supervised exercise programme	Increased number of therapy assistants to carry out supervised exercise programmes	112.2
113	PT100.1	Patients were better informed on the role and potential benefits of exercise for RCRSP	Better informed on the role and potential benefits of exercise for rotator-cuff related shoulder pain	113.1

	PT100.2	Patients were more involved in decision making when prescribing exercise i.e. type, load, frequency, levels of pain etc	More involvement in decision-making when prescribing exercise (e.g., type, load, frequency, levels of pain etc.)	113.2
	PT100.3	Patients were aware of all available treatment options for their condition and how exercise compares to wait and see or more interventional treatment options	Awareness of all available treatment options for shoulder condition and how exercise compares to wait and see approach or more interventional treatment options	113.3
	PT100.4	...any concerns regarding exercising with their particular condition were acknowledged and explored	Acknowledging and exploring any concerns regarding exercise	113.4
	PT100.5	If appropriate the proposed mechanisms underpinning exercise therapy for their condition are explained	Explaining the proposed mechanisms underpinning exercise therapy when appropriate	113.5
114	PT101.1	pateints could see a clear process from where they are to where they want to be	Patients able to see clear process to get from where they are to where they want to be	114.1
	PT101.2	Visual process map	Visual map of the process	114.2
115	PT102.1	Patients were educated that they need to load the tendons to promote healing & to maintain good joint function	Educated on the need to load tendons to promote healing and maintain good joint function	115.1
	PT102.2	Patients were better educated on the normal age related findings on MRI	Better educated on the normal age-related findings on MRI	115.2
	PT102.3	They are understanding that the exercise route will take time & patience	Understanding that exercise route will take time and patience	115.3
116	PT 103.1	Patients were given a realistic time frame for improvement with an exercise programme	Given realistic timeframe for improvement with an exercise programme	116.1
	PT103.2	The exercise programme provided should be clear and simple	Clear and simple exercise programme provided	116.2
	PT103.3	The patient should be encouraged to commit to a specific amount of time per day for their exercises	Encouraged to commit to a specific amount of time per day for their exercises	116.3
	PT103.4	The patient should be encouraged to keep a training diary	Encouraged to keep a training diary	116.4
	PT103.5	A timeframe for follow up appointments should be agreed	Agree a timeframe for follow-up appointments	116.5
117	PT104.1	.patients were better informed on what to expect in terms of pain when exercising	Better informed on what to expect in terms of pain when exercising	117.1
	PT104.2	patients are directed to physiotherapist always before injections	Patients always directed to physiotherapy before injections	117.2

	PT104.3	Patients see Physios before waiting (up to 4 weeks and more) for MRI	Patients referred for physiotherapy before waiting for MRI (up to 4 weeks or more)	117.3
	PY104.4	Patients see physio prior to surgery to build up shd and show whats expected after surgery	Patients attend physiotherapy pre-surgically to build up strength and show what is expected after surgery	117.4
118	PT105.1	there was better quality evidence.	Better quality evidence	118.1
	PT105.2	patients were more discerning in their sources of information on shoulder issues.	Patients were more discerning regarding their sources of information to inform their understanding of their shoulder issue	118.2
	PT105.3	clinicians spent more time educating patients and offering patients reliable sources of information.	More time educating patients and offering reliable sources of information	118.3
119	PT106.1	GPs provided support on conservative over medical intervention as being more efficacious	GPs provided support and education relating to the efficacy of conservative management over medical intervention	119.1
	PT106.2	Plans are structured over a period with increasing intensity and check in	Treatment plans are structured over a period with increasing intensity and progress check-ins	119.2
	PT106.3	Clients attended for rehab in a gym/rehab setting with other people	Attendance for rehabilitation in a gym or rehabilitation setting with other people	119.3
	PT106.4	Data on success and failure rates for surgery were presented	Data on success and failure rates of surgery presented to patient	119.4
120	PT107.1	patients had better understanding that certain pain is ok when exercising	Better understanding that certain pain is ok when exercising	120.1
	PT107.2	research was more clinically based and applicable to clinical practice	Research was more clinically based and applicable to practice	120.2
	PT107.3	clinical applications	Clinical applications	120.3
	PT107.4	less survey's and research done to get the marks and degree and more that would put some relevance into what we do	Less research and more that would put some relevance into what we do	120.4
121	PT108.1	If the referrer to physiotherapy was more informed of best evidence	If those referring into physiotherapy were more informed of best evidence	121.1
	PT108.2	If patients were not given unrealistic timelines by others	If patients were not given unrealistic timelines by others	121.2
	PT108.3	If all information was accessible	If all information was accessible	121.3
	PT108.4	Patients had timely access to physiotherapy, GP, **Orthopedic**	Timely access to physiotherapy, GP, and **Orthopedic**s	121.4

	PT108.5	If advanced practice physiotherapy was in place in public service to offer first contact physiotherapy in networks	If advanced practice physiotherapy was in place in public health service to offer first contact physiotherapy networks	121.5
122	PT109	Patients received summaries of best evidence eg infographics	Patients provided with best-evidence summaries (e.g., infographics)	122
123	PT110	Fewer exercise prescriptions to maintain compliance	Fewer exercises prescribed to help maintain compliance	123
124	PT111.1	Patients were better informed of the benefits of strenghtening exercises to reinforce shoulder	Better informed of the benefits of strengthening exercises to reinforce shoulder	124.1
	PT111.2	patients better understood imaging and the lack of association with pain	Better understood imaging and the lack of association with pain	124.2
	PT111.3	patient was advised on posture and strengthening exercises to improve position of GHJ	Advice on posture and strengthening exercises to improve position of the glenohumeral head	124.3
	PT111.4	patients was shown lots of images of torn rotator muscles in relation to above re imaging	Patient shown torn rotator cuff muscles on imaging in asymptomatic people	124.4
	PT111.5	lots of information if available re success of surgery on above	Available information on success of surgery for rotator cuff tears	124.5
125	PT112.1	we were clear with patients about the timescale for improvement	Clear about the timescale for improvement	125.1
	PT112.2	If GPs were better informed regarding evidence based treatment options for shoulder pain	GPs were better informed regarding evidence-based treatment options for shoulder pain	125.2
	PT112.3	if all physiotherapists involved in managment shoulder pain ensured that they were using best evidence treatment	If all Physiotherapists involved in shoulder pain management ensured that they were using best evidence treatment	125.3
	PT112.4	if all healthcare providers were better informed regarding pros and cons of all treatment options and confidently discussed this with patients	If all healthcare providers were better informed regarding pros and cons of all treatment options and confidently discussed this with patients	125.4
	PT112.5	if there was consistency internationally regarding exercise dose, exercise type, exercise intensity for shoulder conditions	If there was consistency internationally regarding exercise dose, exercise type, exercise intensity for shoulder conditions	125.5

126	PT113.1	there was more robust pathways to ensure consistent patient experience. Ensuring that from first contact (GP, Physio, A and E) the message is consistent. As we know once a patient expectation is formed it can be highly difficult to chnage this.	There were more robust pathways to ensure consistent patient experience	126.1a
			Ensuring that from first contact (GP, Physio, A&E) that the setting of patient expectations and overall message to the patient is consistent.	126.1b
	PT113.2	improved understanding from all HCPC's seeing patients with shoulder pain. Understanding that referrals will be bounced back if patients have not gone through an appropriate first line management course.	Improved understanding from all HCP's seeing patients with shoulder pain that referrals will be bounced back if patients have not gone through appropriate first-line management.	126.2
127	PT114.1	Patients were not expecting full short-term resolution	Patients were not expecting short-term full resolution of symptoms.	127.1
	PT114.2	Patients were convinced that their exercise routine was the principle weapon in their recovery	Patients were convinced that their exercise routine was the principal weapon in their recovery	127.2
128	PT115.1	HCPs messages to patients were more unified.	HCPs messages to patients were more unified.	128.1
	PT115.2	Patients have been involved in setting goals.	Patients have been involved in setting goals.	128.2
	PT115.3	Clear evidence based guidelines were made more available to clinicians	Clear evidence-based guidelines were made more available to clinicians	128.3
129	PT116.1	patients had they're condition explained to them and likely outcomes	The condition and likely outcome were explained to patients.	129.1
	PT116.2	Patients were given a realistic time frame for improvement with exercise therapy/conservative treatment	Patients were given a realistic time frame for improvement with exercise therapy/conservative treatment	129.2
	PT116.3	patients were helped with some form of immediate pain relief/decrease	Patients were helped with something to provide immediate pain relief.	129.3
	PT116.4	patients were helped with their night pain	Patients were helped with their night pain	129.4

130	PT117.1	the HCP was able to quote the research findings to support any specific exercise protocol and written reference or the patient to follow-up if they want.	The HCP was able to quote the research findings to support any specific exercise protocol	130.1a
			Patient provided with a written reference to any research findings discussed and the opportunity to or follow-up on these if they want.	130.1b
	PT117.2	the HCP could explain pain and how "hurt" is not "harm" to help manage pain associated with exercise	HCP could explain pain and how “hurt” is not “harm”	130.2a
			HCPs could explain pain to help manage pain associated with exercise	130.2b
	PT117.3	if the patient recieved a booklet containing all the information recieved during the consultation, as patients forget so much of what they hear, especially if in pain	If the patient received a booklet containing all the information received during their consultation to help them to remember this information, as it can be difficult to concentrate when in pain.	130.3
	PT117.4	the patient was asked to set goals for recovery and given a handout to record their progression (or not!)	Patients were asked to set goals for recovery	130.4a
			Patients were given a handout with the option to record their recovery progression	130.4b
	PT117.5	the HCP was enthusiastic and interested in the patient's recovery	If the HCP was enthusiastic and interested in the patient’s recovery	130.5
131	PT118	Regular consistent exercise	The patient participated in regular consistent exercise	131
132	AT1.1	Patients understood Disturbed sleep can exacerbate shoulder pain	If patients understood disturbed sleep can exacerbate shoulder pain	132.1
	AT1.2	Getting rid of shoulder pain takes time, be patient	Getting rid of shoulder pain takes time and they need to be patient.	132.2
	AT1.3	Patients need to know the conservative approach is more beneficial than surgery. ,	Patients need to know that the conservative treatment approach is more beneficial than surgery	132.3
133	OC7.1	Patients understand the time scale involved in rehabilitation	Patients understand the timescale involved in rehabilitation	133.1

	OC7.2	A realistic understanding of likely benefits from treatment	Patients has a realistic understanding of the likely benefits of treatment	133.2
	OC7.3	An understanding of additional treatment options (injections, surgery)	Patients understood additional treatment options (incl. injections and surgery)	133.3

Round 2: Condensing decisions – grouping of revised healthcare provider educational priorities (R1), based on thematic similarities, creating final statements (R2), each reflecting the overall interpretation of grouped R1 priorities.

Priority ID after R1	Revised priority after R1	Other R1 priorities that are same/similar	Final statement after R2	Final Statement ID
1.1	What level of functional return to expect at various intervals along the rehabilitation journey.	1.2a What to expect in terms of healing times.<br>1.2b What to expect in terms of rehabilitation timeline.<br>1.2c What to expect in terms of timeline to return to sport.<br>1.3 What can be expected in terms of pain and function in the long-term when comparing between treatment options available.<br>2.5 Emphasizing the need for patience with rehabilitation programme<br>7.3b Timeline of rehabilitation post-surgery<br>8.1e Manage patient expectations<br>9.1b Setting expectations for how long it might be to feel noticeable improvement<br>15.3 Recovery timeline<br>20.1a Fully informed on recovery timescales<br>20.1b Recovery expectations (i.e., normal to plateau at times, some activities may be uncomfortable for longer periods)<br>22.2 HCPs and patients aware of timescales for functional recovery<br>25.2 Information regarding approximate lengthy timeline to symptom resolution<br>33.4 Body needs time to heal and/or adapt<br>34.1b Early education at initial consultation regarding predicted recovery timeline<br>39.3 What to expect in response to exercise rather than solely information on the exercise<br>41.1a Information on time to recovery<br>42.5b Improved education on the timeframes involved in rehabilitation<br>45.1 Understanding that improvement may take several weeks<br>49.4 Understanding of duration of recovery<br>51.2 Provision of realistic time guidelines for resolution of pain with or without surgical intervention<br>51.5b Clear and realistic outcome measures<br>52.1b Managing expectations in relation to timelines of first-line treatment	Patients were informed on what to expect in terms of level of recovery at various time intervals depending on the chosen treatment, setting expectations that improvement with conservative treatment can often take an extended period (e.g., minimum 12 weeks).	1

		54.3 The basic healing stages and time frames<br>54.4 That change and healing takes time<br>56.1 Understanding of why symptoms take so long to improve<br>58.2 Given potential timeline for how long rehabilitation would take<br>59.1b Education about the prognosis<br>60.2b Understanding of timescale for building up muscles<br>62.1 Awareness of typical timelines to expect to see improvements<br>66 Explanation that it may take up to six weeks to feel the benefit of exercise<br>70.2 Expectations of what response to expect from treatment and when to expect this<br>74.1c HCPs take time to educate on time frames<br>76.1 Communication of recovery timelines emphasizing this is not a quick fix<br>80.2 Setting expectations in first session relating to timelines for strengthening programmes<br>81.3a Knowledge and understanding of timeline to see results with exercise<br>82.3 Patients and HCPs aware the need to exercise for 12 weeks in order to see effectiveness and visible gains<br>83.1b Explanation of timeframe involved with exercise therapy<br>87.1 Physiotherapists clear with patients regarding expected timeframe for improvement<br>88.4 Provision of management plan with clear timeframes<br>88.5b Importance of consistency and patience when adhering to advice and exercise given<br>92.2 Better informed about timeframe to see results with conservative treatment<br>97.2 Clear communication of expectations in relation to pain and timeframes<br>99.2 Timelines within which to expect improvement<br>101.3 Improved HCP skills to understand patient objectives and expectations<br>105.1b Understanding of likely duration of non-operative management<br>106.5b Understanding that success is not guaranteed<br>107.1a Setting expectations<br>108.1a Clear information about the timeframes for improvement with treatment for their condition		

		109.3 Given realistic timeframe for expected improvement<br>110b Better understanding that with time and understanding there is a strong likelihood it will settle<br>111.1 Better informed regarding expected timeframes when they commence exercise therapy<br>115.3 Understanding that exercise route will take time and patience<br>116.1 Given realistic timeframe for improvement with an exercise programme<br>125.1 Clear about the timescale for improvement<br>129.2 Patients were given a realistic time frame for improvement with exercise therapy/conservative treatment<br>132.2 Getting rid of shoulder pain takes time and they need to be patient<br>133.1 Patients understand the timescale involved in rehabilitation		
1.4	Physiotherapists were better able to adapt and individualize rehabilitation	2.4b Avoid giving too many exercises<br>6.1 - Avoidance of overloading with rehabilitation routines<br>8.5b Exercise that was less prescriptive<br>13.5a Exercises that can be completed quickly and easily at home<br>13.5b Exercises performed at home with minimal or no equipment<br>17.3 Provision of simple exercise schedule<br>24.4 Treatments that are quick and could be performed daily (e.g., maximum 3 home exercises)<br>26.1 Individualized exercises specific to ADLs<br>26.4 Low volume of exercise to ensure effectiveness without making adherence tedious<br>32.2 More simplified, quick, and easy-to-do exercises<br>39.1 Patients had less rather than more time to complete<br>39.2 Simple instructions<br>39.4 Exercises built into specific day and demands of the patient<br>44.3a Better understanding amongst physiotherapists in relation to exercise i.e., how to modify dosage based on patient response<br>48.3 Short and easy to follow treatment regimens<br>52.5 Type of exercise given more in-line with patient interests	Physiotherapists were better able to adapt and tailor rehabilitation programmes to suit individual needs, interests, or goals (e.g., considering number of exercises, dosage, available time, level of difficulty, value-based activities etc.).	2

		63.5 Management included exposure to value-based activities<br>70.5 Adapting the ideal exercise schedule with what they can commit to<br>72.3 Proper selection of candidates suitable for exercise therapy<br>75.1 Tailored approach<br>75.2 Exercise started at appropriate level for patient<br>80.5b Working alongside patient to help them to figure out how they might fit exercises prescribed into their schedule<br>84.2 Physiotherapists applied functional and kinetic chain approach to exercise prescription<br>88.2 Avoidance of prescribing too many exercises<br>94.3 Simple and easy to follow exercises prescribed<br>95.4 Exercise programmes that are more functional and relevant to patient lifestyle and goals<br>97.3 Exercises prescribed that are more meaningful to patient<br>116.2 Clear and simple exercise programme provided<br>123 Fewer exercises prescribed to help maintain compliance<br>128.2 Patients have been involved in setting goals.		
2.1a	Use of anatomy models to provide education in relation to shoulder anatomy	4.2 Understanding of shoulder pathology<br>35.4a Education regarding anatomy<br>37.3 Better understanding of shoulder pathology<br>45.3 Understand the nature of the shoulder pain (i.e., impingement, rotator cuff tear, arthritis)<br>52.3 Clinicians more confident in approaching their treatment through having a better understanding of the shoulder complex<br>59.1a Education about the shoulder pathology<br>65b Clear understanding of what structure is involved<br>65c Clear understanding of the mechanism of injury<br>81.1 Using a shoulder anatomy model to explain reason for pain and how it occured<br>83.2 Each step of treatment explained thoroughly using scan results and skeleton models as required to help educate	Healthcare providers educated patients about their shoulder pathology, identifying the specific structure involved to explain the reason for pain and how it occurred. (e.g., using anatomy models and/or scan)	3

2.1b	How exercise can treat the condition.	3 Role of exercise in pain relief<br>7.1b Exercise therapy<br>13.1 Why exercise therapy is the best first-line treatment<br>15.2 Benefits of exercise, specifically strength training.<br>33.1 Understanding of global and specific benefits of exercise<br>34.1d Early education at initial consultation regarding the role of physiotherapy and exercise<br>35.2 First contact practitioner reassurance about the benefits of about the benefits of exercise<br>35.4c Education regarding the benefits of exercise<br>36.5 Recognition that the outcome of physiotherapy may not achieve full resolution of pain and range of movement, but it can significantly improve function and help manage pain.<br>40.1 Understanding that exercise is an effective management strategy<br>40.2 Understand to expect a good recovery if following a suitable exercise programme<br>44.1b All HCPs provided education regarding the benefits of exercise therapy<br>50.1a Informed about exercise therapy<br>55.2 Examples given by clinician of previous clients who improved with exercise<br>61.3b Pain generally decreases as strength and function improves<br>67.3b Education on the benefits of an exercise programme<br>67.5b More time spent supporting acceptance that pain may not always go away but focus of treatment is on function<br>78.1b Basic knowledge of exercise<br>99.4 Provide empirical examples of process of conservative management<br>104.4 Clear testimonials from patients having successfully undertaken conservative management (with video of pre- and post-treatment pain and range of movement)<br>113.1 Better informed on the role and potential benefits of exercise for rotator-cuff related shoulder pain<br>115.1 Educated on the need to load tendons to promote healing and maintain good joint function<br>124.1 Better informed of the benefits of strengthening exercises to reinforce shoulder	Healthcare providers and patients knew the benefits of exercise and its effectiveness as a first-line treatment strategy, providing patient testimonials in support of this.	4

2.2a	Explanation in relation to expected pain patterns	5.2 Understanding of pain threshold<br>7.1e Likelihood of reoccurrence of pain<br>26.2b Fluctuations in pain levels day-to-day and between different exercises is normal part of rehabilitation<br>44.3b Better understanding amongst patients in relation to exercise i.e. manage flare-ups etc<br>56.3 Understanding of why activity levels must be managed based on their pain<br>70.3 Managing a flare-up of symptoms or a set-back in their recovery<br>108.1b Clear information about how much pain is acceptable	Patients were provided with guidance on how to manage flare-ups/fluctuations in pain levels in response to normal daily activities or exercise	5
2.2b	What to expect in terms of pain during exercise	8.1a What to expect in terms of pain when exercising<br>9.1a Setting expectations in terms of pain when exercising<br>23.2b Use shoulder as much as can tolerate<br>32.1 Better information provided to manage expectations relating to pain while exercising<br>36.4 Prescribing exercises and stretching that patients can feel but should be tolerable<br>40.3a Some pain while exercising is acceptable<br>53.1 Patients fully informed prior to commencing physiotherapy programme<br>54.2 That not all rehabilitation exercise-induced pain is bad pain<br>60.3a Understanding of how pain influences muscle performance<br>61.4a Pain while exercising is safe provided the intensity does not exceed 5/10<br>61.4b Pain while exercising is safe provided pain does not get worse after exercise<br>63.1 Patients were better informed on what to expect in terms of pain when exercising<br>64 Patient awareness of the limit of their exercise in discomfort terms and given permission to move their shoulder to that range of movement<br>100.3 Cease prescription of all painful exercises for shoulders<br>117.1 Better informed on what to expect in terms of pain when exercising<br>120.1 Better understanding that certain pain is ok when exercising	Patients knew what to expect in terms of pain during exercise, and what level of exercise-induced pain is acceptable.	6

		130.2b HCPs could explain pain to help manage pain associated with exercise		
2.3	Setting time-based and realistic goals	16.3 Setting realistic expectations for exercise (minimum 4 months consistent adherence)<br>17.2 Realistic view of treatment outcomes<br>29.3 Realistic goals<br>34.4b Realistic short-term goals relating to pain management<br>34.4c Realistic short-term goals relating to sleep<br>34.4d Realistic short-term goals relating to function<br>67.5a More time spent discussing realistic goals<br>84.3a Physiotherapists set achievable visible short-term goals<br>97.4 Creation of meaningful goals with the patient<br>106.5b Understanding that success is not guaranteed<br>127.1 Patients were not expecting short-term full resolution of symptoms.	Patients were given time-based, meaningful, and realistic goals (e.g., relating to sleep, pain management, function)	7
2.4	Regularly changing exercises	8.3 Exercise interventions were more fun and engaging<br>8.5c More choice with exercises<br>8.5d Exercises that were more interesting and enjoyable<br>80.5c Involve patient in process of exercise prescription by providing them with a choice in what exercises they complete<br>108.2 Given choice in their selection of exercises	Involving patients in the process of exercise prescription by providing them with a choice in what exercises they select and complete.	8
4.1a	Understanding of shoulder pain	11.1a GPs had a better understanding of shoulder pain<br>24.3a Understanding of their condition<br>29.1 Full understanding of diagnosis<br>34.1a Early education at initial consultation regarding diagnosis<br>34.3a Understanding of diagnosis and can discuss it<br>35.4b Education regarding diagnosis<br>36.2 A clicking joint can be a good thing<br>49.3 Understanding of pain<br>54.1 Understanding of the causes of their pain	Patients were provided with information relating to their shoulder pain diagnosis, nature of pain, and factors that can cause pain	9

		56.2 Understanding of various factors that caused the occurrence of their pain<br>63.3a Patients and HCPs understood nature of shoulder pain<br>63.3b Patients and HCPs understood behaviour of musculoskeletal shoulder pain presentations<br>65a Clear understanding of their issue<br>72.4 Proper assessment of patient understanding of their condition<br>80.1c Explore beliefs surrounding cause of shoulder pain<br>93.3 Better understanding of their shoulder issue<br>109.1 Understanding of the underlying nature of shoulder pain		
4.1b	Understanding of evidence-based research recommendations	7.1c Shift in treatment focus away from manual therapy treatment<br>11.1b GPs had a better understanding of evidence-based treatment.<br>18.2 Healthcare providers had better understanding of mounting research evidence supporting exercise therapy<br>19.1 Healthcare providers informed about current evidence, clinical practice guidelines and recommended care pathways<br>19.3b Awareness of different management options<br>22.1 Awareness of substantial role of non-operative therapy<br>30.1 If Physiotherapists were better informed<br>31.3 HCPs educated on clinically applicable evidence-based practice.<br>42.4a Improved education around the role of injections and surgery<br>42.5a Improved education on the role of exercise<br>51.1a GPs had a better understanding of the benefits of exercise<br>55.1 Information on the strong evidence to support exercise-based therapy<br>55.4b Explanation for why hands on therapy alone may not have a good outcome<br>62.2a Educated on short-term benefits of manual therapies<br>62.2b Educated that exercise therapy shows more long-term results	Healthcare providers and patients had a better understanding of evidence-based treatment recommendations for shoulder pain, highlighting to patients the support for exercise therapy as first-line treatment rather than 'hands on' therapy, surgery, or injections (incl. which type of exercise and particular dosage is most evidence-based).	10

		62.3 If other HCPs (as well as Physiotherapists) were aware of first-line treatment options<br>65e Clear understanding of what treatment most recent research evidence supports<br>72.2 Exercise completed properly over time can yield quicker results compared to surgery<br>73.2a Improved GP awareness of research evidence for physiotherapy<br>74.1b HCPs take time to educate on treatment options<br>76.2a GPs and Physiotherapist up to date with latest research<br>76.2b GPs and Physiotherapist clear on how to implement latest research<br>81.4a Understanding of why surgery should be avoided initially<br>81.4b Understanding of the benefits of first-line treatments<br>82.3 Guidance for Physiotherapists on specific evidence-based exercises<br>87.2 GPs better informed regarding treatment options<br>87.5 All Physiotherapists kept informed regarding evidence-based practice<br>90.2 Less expectation of manual therapy treatment<br>92.4 Physiotherapists were better informed about the most effective type of exercise prescription (e.g., type of exercise, amount of load etc.)<br>97.5 Better understanding amongst HCPs of the role of physiotherapy and avoidance of directing patients towards non-evidence-based management<br>98.2b Emphasis of physiotherapy treatment on exercise and education not passive therapies<br>100.1 All HCPs were knowledgeable of first-line treatment<br>100.5b Discontinue use of shoulder slings in 2-week period post-injections and advise how long support needed<br>101.4 HCP knowledge of evidence-based treatments<br>101.5 All HCPs were knowledgeable of first-line treatment<br>103.1 Encouragement of HCPs to follow evidenced-based care in rotator cuff-related shoulder pain (RCRSP)<br>105.1a Understanding of goal of non-operative management<br>107.2 Explanation that surgery is rarely needed<br>125.2 GPs were better informed regarding evidence-based treatment options for shoulder pain		

		125.3 If all Physiotherapists involved in shoulder pain management ensured that they were using best evidence treatment<br>130.1a The HCP was able to quote the research findings to support any specific exercise protocol<br>133.3 Patients understood additional treatment options (incl. injections and surgery)		
4.4	Experiences reduced pain levels in response to treatment	4.3 Experiences positive outcomes of treatment<br>24.1 Treatment that demonstrated early signs of improvement in range of motion or pain<br>24.5 Feel improvement post-exercise and see slow gains/improvement<br>34.4a Able to understand and see progress<br>84.3a Setting short-term goals so that patients can see improvements to help get buy-in<br>93.1 Able to see results/improvements in pain with compliance with exercise programmes<br>94.2 If an immediate positive result is noticed	Patients were able to see early signs of progress in relation to pain and range of motion in response to treatments, such as exercise therapy.	11
4.5	Importance of compliance with treatment	20.4a Rehabilitation to continue to late stages of recovery<br>59.3a Need for exercise adherence<br>81.3b Knowledge and understanding of the benefits of persevering with the exercises<br>83.1a Explanation of commitment required with exercise therapy<br>104.1 Awareness of burden of surgical rehabilitation<br>116.3 Encouraged to commit to a specific amount of time per day for their exercises<br>131 The patient participated in regular consistent exercise	Patients understood the benefits and importance of persevering with exercise therapy.	12
5.1	Given statistical results of what outcome to expect post evidence-based treatment	9.1c Understanding what to expect in the short and long-term comparing exercise with surgery and/or injection<br>15.5 Long-term outcomes of physiotherapy better than surgery<br>16.4 Awareness of research evidence supporting exercise combined with addressing lifestyle factors as effective as surgery for atraumatic shoulder pain<br>17.5 Lack of benefit associated with surgery<br>18.3b All HCPs provided advice regarding the lack of effectiveness of surgery<br>28.2 Efficacy of treatment provided	Healthcare providers and patients understood the research evidence comparing the effectiveness, expected long-term outcomes and the benefits and risks associated with different treatment options available.	13

		34.3c Question available treatment routes<br>38.1a Awareness of evidence<br>42.2 Broader understanding of the role of physiotherapy<br>45.2 Understanding of other treatment options available in addition to physiotherapy, such as injections and surgery<br>50.2 Patient preferred steroid injections<br>59.2 Knowledge about rehabilitation process and what to expect<br>60.4 Provision of facts and figures comparing treatment options (e.g., Physiotherapy Vs Injection Vs Surgery)<br>61.5 Long-term outcomes following surgery are often worse than compared to conservative management.<br>68.1 Information on good outcomes following evidence-based management approaches<br>80.4 Education on the available evidence comparing surgery and conservative management at one year follow-up<br>82.1b Education relating to the evidence that surgery is no more effective than exercise<br>87.3 All HCPs were able to speak confidentially to patients regarding the pros and cons of all treatment options<br>93.2 Knowledge of inconsistent and uncertain outcomes following surgery<br>98.1b **Orthopedic** surgeons provide honest information about surgical results<br>99.1c When educating on the pros of non-surgical approach providing information relating to its effects on tissue healing, muscle strengthening, and symmetry of joint kinetics.<br>104.1 Awareness of burden of surgical rehabilitation<br>106.5c Understanding that complications may arise<br>113.3 Awareness of all available treatment options for shoulder condition and how exercise compares to wait and see approach or more interventional treatment options<br>119.4 Data on success and failure rates of surgery presented to patient<br>124.5 Available information on success of surgery for rotator cuff tears<br>125.4 If all healthcare providers were better informed regarding pros and cons of all treatment options and confidently discussed this with patients<br>132.2 Patients need to know that the conservative treatment approach is more beneficial than surgery			

		133.2 Patients has a realistic understanding of the likely benefits of treatment		
5.3a	Provided regular feedback	9.2 Practicing exercises in-person (full sets and reps) during initial appointment.<br>13.3 Allocate time during session to teach exercises and ensure patient confidence in performing these.<br>17.4 Provided with follow-up during course of treatment<br>20.4b Advice provided throughout rehabilitation at each stage<br>25.5b Understanding of how to accurately carry out exercises<br>27.2 Regular input from their HCP throughout treatment programme on an interval basis (virtually or F2F)<br>29.4 Time and ressources to practice exercise<br>74.2 Ongoing communication with topics relating to treatment options and timeframes revisited frequently to help information recall	Patients had opportunities to practice exercises with their Physiotherapist and get ongoing support and feedback throughout their treatment programme (e.g., virtually, or face-to-face).	14
5.3b	Provided regular reassurance	8.1d Provide reassurance<br>34.3b Address patient fears and questions<br>45.4 Understanding that many other patients have similar symptoms<br>74.3 Provision of reasonable level of reassurance<br>75.3 Reassurance regarding prognosis<br>81.5b Provide reassurance regarding progress<br>93.4 Reassurance regarding knowledge of presence of ‘rotator cuff tear’<br>102.1 Avoidance of HCPs who catastrophize patient pain<br>106.4 Reassurance that exercises won’t damage shoulder<br>112.1 Avoid over-diagnosing patients and instead provide reassurance their pain is normal<br>113.4 Acknowledging and exploring any concerns regarding exercise	Healthcare providers acknowledged and explored patient fears and concerns (e.g., regarding exercise), and provided regular reassurance in relation to treatment progress and prognosis.	15
5.4	Clear and unambiguous information on what will improve shoulder pain and what will exacerbate symptoms	7.1d What helps progression<br>13.4 Provision of clear written, visual, and oral information about what patients should do<br>23.3 Rest is not necessarily helpful<br>34.1f Early education at initial consultation on what is required for their injury	Patients were provided with clear and unambiguous information on what will improve shoulder pain and what will exacerbate symptoms (e.g., activity modification, healthy lifestyle etc.)	16

		59.4 Information relating to activity modification in the short and long-term<br>63.3e Patients and HCPs understood management is key<br>70.1 How the treatment will help and why<br>71.1 Education on the benefits of good posture and a healthy lifestyle and the effect this has on muscle function<br>109.2 Ability to link knowledge about the nature of pain to actions that can improve their pain<br>111.2 Patients told what the problem is and what exercise they need to do to help this.<br>114.1 Patients able to see clear process to get from where they are to where they want to be<br>114.2 Visual map of the process<br>124.3 Advice on posture and strengthening exercises to improve position of the glenohumeral head		
5.5	Honest patient-therapist relationship	12.1 Physiotherapists had specific training to enhance and optimise the therapeutic relationship<br>26.3 Strong therapeutic relationship in which patient feels their fears and concerns have been listened to and addressed<br>28.1 Understood relationship between treatment and their goals<br>28.4 HCPs make effort to have a good relationship with their patient<br>29.2 Good relationship with their healthcare provider<br>50.3 Provided patient can manage basic tasks and pain is not severe, patients will rarely opt for surgery<br>55.3 Motivational interviewing skills to find out what is important to the clinician and how to help them achieve their goals<br>55.5 Clinician listens carefully to what the client wants to achieve<br>57.1 Therapist understands the patients' goals<br>80.1a Holistic approach and listening to the patient's whole story<br>80.1b Empathise with patient<br>102.3 Built up a good relationship of trust with treating HCP<br>130.5 If the HCP was enthusiastic and interested in the patient's recovery	Healthcare providers and patients developed and maintained a strong therapeutic relationship, built on honesty and trust, where patients feel their healthcare provider has empathised and listened carefully to their whole story, their goals, and has addressed all their fears and concerns.	17

6.2	More education provided by practitioner to support patient.	19.2 HCPs took their time to explain current evidence<br>30.3 If Physiotherapists could spend more time with the patient<br>37.5 Taking time to educate patients regarding imaging findings or other investigations they've had done<br>67.3a More time spent providing education of their condition<br>74.1a HCPs take time to educate on likely course of treatment<br>97.1 Educating the patient in a format that suited the patient<br>118.3 More time educating patients and offering reliable sources of information<br>129.1 The condition and likely outcome was explained to patients.<br>130.1b Patient provided with a written reference to any research findings discussed and the opportunity to or follow-up on these if they want.<br>130.3 If the patient received a booklet containing all the information received during their consultation to help them to remember this information, as it can be difficult to concentrate when in pain.	Healthcare providers took more time to educate patients regarding their condition, imaging findings and effective treatments, directing them towards reliable sources of information.	18
7.1a	Self-management	8.5a Sense of ownership over rehabilitation<br>13.2 Ownership over exercise programme through giving options and control over progressions/regressions.<br>34.3d Feel they are participating in their recovery process<br>49.5 Understanding of motivation required<br>60.3b Understanding of how to pace<br>67.3c Education on the commitment required and process involved in completing an exercise programme<br>88.5a Education that it will take time and effort to change shoulder pain<br>105.3 the need to engage with rehabilitation as the pain won't fix itself<br>130.4b Patients were given a handout with the option to record their recovery progression	Patients understood the motivation, effort and commitment required for exercise therapy, and felt a sense of ownership and had the confidence to self-manage their rehabilitation (e.g., how to pace, regress and progress exercises)	19

7.2	Recommended treatment pathway to facilitate realistic attempt at physiotherapy before GP referral for imaging and surgical opinion	11.2 **Orthopedic** surgeons referred back to physiotherapy more instead of opting for surgery.<br>21 First point of primary care contact setting patient expectations for physiotherapy and exercise therapy<br>31.2a More of a barrier to second-line treatment options<br>31.2b Better triaging for second-line treatment<br>35.1 Reduced unnecessary referral for imaging<br>42.3 **Orthopedic** consultants promoted the role of physiotherapy and conservative management<br>46.1a GPs better informed about the need for rehabilitation prior to more invasive treatments<br>46.1b GPs prioritised explaining to patients they will need to engage in rehabilitation before considering invasive treatments<br>47.1 GPs better understanding of the evidence behind shoulder pain rather than referring everyone for injections<br>51.1b GPs did not automatically refer for surgery based wholly on imaging findings<br>51.3 Promotion of the benefits of non-surgical intervention in GP and consultant clinics<br>52.1a Managing expectations in relation to first-line treatments<br>52.4 Managing expectations regarding surgery<br>67.2c Patients should not be sent for routine scans unless there is a clear indication<br>71.2 Understanding of aim of management pathways<br>79.1 Better informed GPs given they are first point of contact<br>84.1 **Orthopedic** consultants encouraged patients to adhere to conservative management<br>89.1 **Orthopedic** surgeons stipulated any patients completed a 12-week rehabilitation programme (3 x a week) prior consideration for surgery<br>91.1 GPs and **Orthopedic** consultants strongly encouraged conservative management at the outset.<br>95.3 Clear pathway with access to someone knowledgeable relating to evidence-based management recommendations<br>96.2 Promotion of exercise as best treatment intervention<br>96.3 Greater compliance from GPs and **Orthopedic** surgeons to evidence-based recommendations as opposed to injections and surgery as first line treatment	All HCPs, in particular first-contact HCPs (e.g., GPs), promoted and set early patient expectations for first-line treatment options, while also reducing the frequency of unnecessary referral for imaging and surgical opinion.	20

		98.1a Educated by surgeons to do physiotherapy first for a minimum of 3 months<br>99.1b Why non-surgical approach is the best first-line treatment<br>101.1 Increased HCP confidence in educating patients<br>103.2 GPs didn't refer simultaneously to **Orthopedic**s and physiotherapy<br>117.2 Patients always directed to physiotherapy before injections<br>117.3 Patients referred for physiotherapy before waiting for MRI (up to 4 weeks or more)<br>119.1 GPs provided support and education relating to the efficacy of conservative management over medical intervention<br>121.1 If those referring into physiotherapy were more informed of best evidence<br>126.1a There were more robust pathways to ensure consistent patient experience<br>126.2 Improved understanding from all HCP's seeing patients with shoulder pain that referrals will be bounced back if patients have not gone through appropriate first-line management.		
7.3a	Implications of surgery	7.3c Emphasize surgery is not a quick-fix solution<br>20.2 Surgery is not a "quick fix"<br>41.3 Surgery is not a quick solution<br>42.4b Improved education that injection and surgery do not provide a quick fix approach<br>63.3f Patients and HCPs understood there is no 'fix' available<br>106.5a Understanding that surgery is not a quick fix<br>117.4 Patients attend physiotherapy pre-surgically to build up strength and show what is expected after surgery	Healthcare providers and patients understood that injection and surgery don't offer a 'quick-fix' solution	21
8.1b	Feeling supported by healthcare providers	8.1c Had identified point of contact to engage with if there was any uncertainty about rehabilitation journey.<br>9.3 Follow-up phone call to check-in with patient between appointments.<br>27.1 Appropriate information provided prior to and throughout treatment programme<br>38.2 Provision of aids to help e.g., apps, resources, diaries<br>41.2 Reminder to do exercises (e.g., app)<br>41.4 Provide support to help control compliance and avoid leaving patients alone (e.g., videocall)	Patients had regular follow-up contact with their healthcare providers throughout the rehabilitation journey, as well as supportive resources to help them to continue exercising (e.g., reminders, exercise tracker APPs, diaries, printed exercise sheets)	22

		48.4 Regular re-assessment with exercise prescribers<br>53.3 Regular virtual contact from treating clinician to maintain motivation and maintain correct information input<br>60.1 Reminders or APP to track exercise and remind them of their programme<br>77.3 Regular rehabilitation with tracking of progress and objective improvements<br>80.5a Provision of print out of exercises for patient<br>81.5a Regular follow-up to ensure exercises completed correctly<br>99.3 Monitoring throughout recovery (either F2F/phone/video call) to encourage compliance<br>100.4 Continuous assessment and re-assessment of the patient<br>104.3b Opportunity for patient to review information provided at their initial clinical review at a later date (via website/educational video)<br>108.4 Regular reminders to do exercises (e.g., from an APP)<br>109.4 Pathway back to healthcare provider if no improvement<br>116.4 Encouraged to keep a training diary<br>116.5 Agree a timeframe for follow-up appointments<br>119.2 Treatment plans are structured over a period with increasing intensity and progress check-ins		
8.2a	Understand lack of association between shoulder pain and imaging findings	12.4 If GPs were more knowledgeable about the lack of correlation between structural pathology and clinical symptoms.<br>16.2 Awareness that in the absence of significant trauma, often morphological changes on imaging not the reason for symptoms<br>25.4 HCPs focus on identifying the driver for the problem rather than the pathological tissue<br>40.5a Adverse findings on imaging do not equate to symptoms<br>43.2b HCPs educated patients regarding pain and the lack of correlation with structural pathology on MRI scans<br>61.3a MRI findings do not always correlate with the source of pain<br>63.2 Patients and HCPs better understood normal imaging findings and their lack of association with shoulder pain	Healthcare providers and patients understood that imaging findings do not always correlate with pain	23

		67.2a Patients and HCPs understood normal imaging findings and their lack of association with shoulder pain<br>70.4 Explaining imaging findings and relevance to the person<br>78.2 Greater awareness amongst HCPs that imaging findings are normal and not the cause of their pain<br>80.3 Understanding that imaging findings of symptomatic shoulder can be the same as another person of similar age with no symptoms<br>91.3 Patients and HCPs better understood normal imaging findings and their lack of association with pain<br>95.2 Understanding that the changes seen on imaging don't necessarily relate to their pain<br>101.5 Improved skills of HCPs to indicate diagnosis without the need of imaging<br>105.2 Imaging findings don't always correlate with clinical problem<br>106.2 Understanding that positive findings on imaging and examination can be physiological rather than pathological<br>124.2 Better understood imaging and the lack of association with pain		
8.2b	Understanding of what normal imaging findings to expect	15.4 Scan education<br>34.1e Early education at initial consultation regarding role of imaging<br>49.1 Understanding of imaging findings<br>58.3 HCPs were better able to help patients understand the results of imaging<br>60.5 Facts regarding imaging and what normal findings would be<br>65d Clear understanding of the relevance of radiology<br>67.2b Provision of explanation of scan provided to patient in understandable language<br>96.1 Better explanation of imaging findings<br>99.5 Explaining normal age-related changes seen on imaging<br>103.3 Better explanation of imaging findings and normal age-related findings<br>115.2 Better educated on the normal age-related findings on MRI<br>124.4 Patient shown torn rotator cuff muscles on imaging in asymptomatic people	Patients had a clear understanding of the relevance of radiology (if any) and what normal age-related changes would be expected.	24

8.4a	Unified and consistent message from healthcare providers in relation to imaging findings	8.4b Unified and consistent message delivered from healthcare providers in relation to best management approach<br>10.1 Healthcare providers communicating the same management strategy.<br>12.3 Greater collaboration and agreement between **Orthopedic** surgeons and physiotherapists in how to present evidence-based findings.<br>16.1 Coherent message delivered from all involved<br>18.3a Cohesive advice from all healthcare providers<br>19.3b Provision of clear management plan<br>20.3 Surgeons on board with the same message<br>31.1 Consistent information provided by different HCPs<br>32.3b Evidence-based guidelines that were agreed<br>43.2a HCPs were consistent in their messaging to patients<br>44.1a Same message from all HCPs caring for patient<br>46.2a Consistent message delivered from all HCPs<br>48.2 Consistent information from all healthcare providers<br>52.2 Advice given by **Orthopedic** surgeons/other medics more cohesive with advice given by Physiotherapist<br>73.3 For GPs and **Orthopedic** consultants to have knowledge of best-practice first line treatment options so all HCPs singing from same hymn sheet<br>82.1a Consistent message delivered to patients<br>87.4 International agreement regarding exercise type and dosage for various shoulder conditions<br>92.1 All HCPs were signing off the same hymn sheet as regards the effectiveness of conservative treatment<br>125.5 If there was consistency internationally regarding exercise dose, exercise type, exercise intensity for shoulder conditions<br>126.1b Ensuring that from first contact (GP, Physio, A&E) that the setting of patient expectations and overall message to the patient is consistent.<br>128.1 HCPs messages to patients were more unified.	Healthcare providers delivered a unified and consistent message in relation to the best management approach, with greater collaboration and agreement on how best to present evidence-based findings to patients.	25
8.5e	Exercise with a social component	47.4 More specific shoulder classes for patients to attend<br>49.2 Access to group supervised exercise classes<br>89.5 Education that on-site supervised rehabilitation is the gold standard<br>92.5 Availability of high-quality group classes in outpatient departments<br>102.2 Access to supervised exercise facilities	Patients had access to high-quality supervised group-based exercise classes (e.g., specific shoulder classes, clinical Pilates)	26

		102.4 Provision of clinical pilates and exercise classes<br>112.2 Increased number of therapy assistants to carry out supervised exercise programmes<br>119.3 Attendance for rehabilitation in a gym or rehabilitation setting with other people		
10.2	Not only **Orthopedic** surgeons were considered 'specialists' in this field.	30.2 If Physiotherapists were better paid<br>77.2 The main healthcare provider was not a doctor<br>77.4 Physiotherapists considered equal to medical doctors<br>95.5 Acknowledgment of physiotherapists as equals amongst the HCP team and not something "to try" to improve likelihood of patients pursuing conservative management	All members of the healthcare team were considered equal.	27
10.3	Popular media informing 'our' story	14.3 Multi-model campaign involving media to educate public about shoulder pain, the role of exercise and why MRI and surgery are not the answer.<br>47.2 More public knowledge of managing shoulder pain<br>69 Public better informed as to the importance of their role in being actively involved in their recovery rather than having a passive attitude to treatment<br>78.1a Better exercise culture in Ireland<br>86.3 Promotion of physical activity<br>89.3 Public campaign to increase awareness of the need to complete a 12-week programme<br>93.5 Patients didn't believe inaccurate information based on other people's experiences<br>118.2 Patients were more discerning regarding their sources of information to inform their understanding of their shoulder issue<br>121.2 If those referring into physiotherapy were more informed of best evidence	The public were better informed about shoulder pain, the role of exercise, benefits of physical activity, why MRI and surgery are not the answer, and the importance of active involvement in the recovery process (e.g., via public/media campaigns)	28
12.2	Specific training for healthcare providers on how to discuss evidenced-based treatments with patients, including pros/cons/risks/benefits of treatments, to support informed choices.	16.5 Shared decision-making in relation to management plan and its implementation (e.g., if exercise therapy– what's realistic in terms of frequency?)<br>19.2b HCPs engaged in shared decision-making<br>25.5a Checking patient understanding of explanation given<br>34.2 Good communication between all involved HCPs and patient as appropriate	There was better communication amongst HCPs and the patient in relation to discussing evidence-based treatments and outcomes, supporting shared decision-making and more informed treatment choices.	29

		34.5 Better feedback and communication with referrers irrespective of outcome (i.e., Good, or bad recovery in response to treatment)<br>37.2 Team-based approach to managing care<br>67.1 Joint decision-making about treatment early in care between HCP and patient<br>68.5 All HCPs working together<br>92.3 Better communication between hospital consultants and primary care physiotherapists<br>113.2 More involvement in decision-making when prescribing exercise (e.g., type, load, frequency, levels of pain etc.)		
14.1b	HCPs given access to guidelines that they could read on their desktops	14.1a HCPs given clear, easy to read guidelines<br>14.2 Short and concise GP-orientated educational workshops, ran at suitable times<br>19.4a Consumer access to evidence-based information<br>32.3a Evidence-based guidelines were easily accessible<br>37.4 Access to evidence-based standardised information regarding managing shoulder pain<br>46.2b All HCPs using national shoulder advice booklet or website to refer to<br>51.5a Standardised protocol that can be followed by both patients and HCPs<br>53.2 Access to information for patients throughout their programme from the provider e.g., web-based information<br>63.4 Patients and HCPs received appropriate education<br>68.3 More educational workshops and seminars<br>79.3 Trusted website for patients with information and exercises that would complement 1:1 physiotherapy sessions<br>86.1 Improved technology<br>90.1 Research evidence more readily accessible to HCPs<br>95.1 Access to the most up-to-date evidence-based recommendations communicated in a non-biased way<br>101.2 Easy access to resources containing education and exercise information available for patients<br>102.5 Good database of information available through the Irish Society of Chartered Physiotherapists (ISCP)<br>118.1 Better quality evidence<br>120.2 Research was more clinically based and applicable to practice<br>120.3 Clinical applications	Evidence-based guidelines and information for HCPs and patients regarding managing shoulder pain were more easily accessible (e.g., national advice booklet, website, workshops, seminars etc.)	30

		120.4 Less research and more that would put some relevance into what we do<br>121.3 If all information was accessible<br>128.3 Clear evidence-based guidelines were made more available to clinicians		
15.1	Pain education	18.1 Healthcare providers improved understanding of neurophysiology of pain<br>23.2a Understanding that pain does not equal harm<br>26.2a Pain does not mean damage<br>28.3 Pain science<br>36.1 Pain does not equal damage<br>40.3b Pain during exercise is neither harmful of something to be anxious about<br>41.1b Information on pain during the recovery period<br>63.3c Patients and HCPs understood that sinister pathologies or diseases were rare<br>63.3d Patients and HCPs understood flare-ups are normal and do not indicate something sinister<br>91.2 Understanding of pain science<br>130.2a HCP could explain pain and how “hurt” is not “harm”<br>132.1 If patients understood disturbed sleep can exacerbate shoulder pain	Healthcare providers and patients had an understanding of pain science, in particular, that pain does not always equal harm or indicate the presence of something sinister (e.g., pain during exercise)	31
17.1	Timely appointment with physiotherapy or similar	35.3 Reduced length of waiting lists to avoid patients being disheartened and frustrated by the time they are seen<br>37.1 Early access to first-line treatment<br>42.1 Provision of treatment in a timely fashion<br>47.3 Reduced length of waiting lists in primary care<br>73.1 Option to self-refer to local health service to access first-line treatment from Physiotherapy<br>73.4a Improved waiting times to reduce risk of chronicity of symptoms<br>73.4b If patients on waiting list had option to attend once-off information session on shoulder pain and rehabilitation before initial appointment with physiotherapy to allow time to take in information given<br>89.4 Improved GP understanding of importance to refer patients for physiotherapy once pain medication prescribed and not waiting until course of medication is complete	Healthcare providers understood the need to refer for first-line treatment in a timely manner, and to provide patients with information about shoulder pain and rehabilitation for those on waiting lists to help reduce risk of chronicity and patient frustration.	32

		98.2a Access to quality physiotherapy quickly<br>121.4 Timely access to physiotherapy, GP, and **Orthopedic**s		
18.4	Immediate provision of adequate pain management options	34.1c Early education at initial consultation regarding the role of analgesia<br>39.3 Advice on how to manage pain levels<br>50.1b Informed about pain relief<br>89.2 Comprehensive pain management running parallel with rehabilitation<br>100.5a Urgent use of corticosteroid injections if signs of frozen shoulder (reduced lateral rotation, abduction, and medial rotation)<br>106.3 The need for adequate pain control during non-surgical treatment<br>107.5 Treatment of frozen shoulder with pain relief (e.g., steroid injection) and not the need for MRI<br>129.3 Patients were helped with something to provide immediate pain relief.<br>129.4 Patients were helped with their night pain	Patients were better informed about how to manage pain, including the role of analgesia to relieve pain and support engagement with rehabilitation.	33
19.4b	Evidence-based information in simple plain language for consumers	24.2 Easy to understand infographics from evidence-based research<br>48.1 Written information on their shoulder problem<br>85 Simple information<br>99.1a Physiotherapists provide education in simple language<br>104.3a Provision of clear, concise, understandable information at the time of clinical review<br>108.3 Given clear exercise prescription with high quality resources (incl. vidoes, handouts etc.)<br>122 Patients provided with best-evidence summaries (e.g., infographics)	Healthcare providers and patients were provided with clear, concise understandable evidence-based information (e.g., infographics, videos, handouts)	34
22.3	Patients and HCPs understanding that imaging findings are not the only factor involved in management decisions	25.3 Lack of correlation between imaging and outcomes<br>36.3 Knowledge that irrespective of outcome of imaging, will need to get the shoulder moving and start using it again.<br>40.4 Imaging is not necessary for the successful management of shoulder pain<br>40.5b Adverse findings on imaging do not correlate with time to recovery	Healthcare providers and patients understood that imaging is not necessary for the successful management of shoulder pain and is only appropriate for cases that do not resolve after first-line treatment, or if red flags are present.	35

		51.4 Imaging should only be used if necessary where there has been no improvement with physiotherapy<br>62.4 Imaging only appropriate for cases that do not resolve after first-line treatment or if red flags are present		
23.1	Better information provided to explain rationale for type of exercise prescribed	43.1 Greater emphasis on patient education as part of rehabilitation programme (i.e., group information)<br>55.4a Explanation for the rationale of the exercises<br>59.3b Rationale for specific exercises<br>60.2a Understanding of how muscle tissue builds<br>72.1 Explain that exercise is necessary for recovery regardless of whether surgery is required or not<br>76.3 Improved adherence if better educated as to the reason why doing specific elements of treatment<br>81.2a Education to explain reason for exercise programme<br>81.2b Education on the purpose and goal of each exercise<br>88.1 Good understanding of why they are doing the exercises<br>94.1 Clear explanation given as to why treatment is prescribed<br>104.2 Clear understanding of reasons and rationale behind conservative management<br>113.5 Explaining the proposed mechanisms underpinning exercise therapy when appropriate	Physiotherapists placed greater emphasis on patient education as part of rehabilitation, with clear understanding of rationale behind conservative management and proposed mechanisms underpinning these	36
24.3b	Understanding of the difficulties in making a diagnosis	58.1 Understanding why much of shoulder pain can be classified as non-specific	Patients knew that shoulder pain is difficult to diagnose, and much pain can be classified as non-specific.	37
25.1	Lifestyle factors and their influences	34.3e Understanding of the psychological factors that can influence their pain<br>35.5 Acceptance that psychological factors influence outcomes<br>38.5 Not dealing with other joint issues and comorbidities<br>67.4 More time spent on discussing other contributing factors to shoulder pain e.g., stress, anxiety, obesity, and smoking<br>71.3a Educated about the stress response<br>71.3b Educated about fear avoidance	Patients were provided with more education relating to factors that can impact recovery potential and contribute to shoulder pain (e.g., stress, obesity, smoking, fear avoidance, comorbidities etc.)	38

31.4	Health care policy relating to shoulder pain more aligned with current best evidence.	44.2 Emphasis throughout the healthcare system on active treatment and management rather than on investigation, in particular, within primary care<br>46.3 Better funding or compensation for patients to engage in rehabilitation irrespective of private or public healthcare<br>77.1 Sick leave was also provided for treatment other than surgery	Healthcare policy relating to shoulder pain was more aligned with current best-evidence, with improved financial support for patients to engage in rehabilitation irrespective of public or private healthcare.	39
33.2	Understanding of age-related changes and accept these are normal part of life	33.3 Understanding can't be asymptomatic all their life<br>61.1 Knowledge that shoulder pain and rotator cuff injury is normal<br>61.2 Knowledge that 1 in 2 people will experience shoulder pain relating to rotator cuff tear at some point in their lives<br>110a Better understanding that pain is a normal part of the human experience	Patients understood that shoulder pain is common and age-related changes are a normal part of life.	40
38.1b	Patient belief that evidence-based treatment will work	38.4 High self-efficacy<br>88.3 Strong patient belief in the potential of exercise to help pain and function<br>108.5 Patients identified as having poor prognostic risk factors such as low self-efficacy could access more physiotherapy input if required<br>127.1 Patients were convinced that their exercise routine was the principal weapon in their recovery	Healthcare providers and patients had an awareness of positive prognostic indicators such as high self-efficacy and the belief that evidence-based treatment will work.	41
68.2	HCPs conduct more research (e.g., RCTs) to determine the best approach to management	68.4 More financial funding for researchers	There was more Information for healthcare providers to support research exploring the best management approach for shoulder pain, with more financial funding available to support these activities.	42
86.2	Improved health literacy at younger age		There were more resources available to improve public health literacy from a young age.	43
106.1	Understanding of the natural history of atraumatic shoulder pathologies	107.1b Explained natural history	Healthcare providers and patients understood the natural history of atraumatic shoulder pain	44
100.2	Referral to physiotherapists with a special interest in shoulder pain recognised nationally and internationally with a shoulder sign	73.2b Education for GPs on musculoskeletal management of shoulder pain by clinical specialist physiotherapists working in Triage services.	If a patient's first contact involves access to specialist or advanced-practice shoulder physiotherapists with the ability to directly access imaging, if required.	45

		79.2 **Orthopedic** Surgeons used trust group of specialist shoulder physiotherapists to treat more complex<br>107.3 Stopping physiotherapists in intermediate care from requesting MRI scans<br>107.4 Allowing physiotherapists rapid access to simple shoulder X-Rays<br>presentations<br>121.5 If advanced practice physiotherapy was in place in public health service to offer first contact physiotherapy networks		

Healthcare Provider Final Statement List (for inclusion in importance rating survey)

Final Statement ID	Final Statements

1	Patients were informed on what to expect in terms of level of recovery at various time intervals depending on the chosen treatment, setting expectations that improvement with conservative treatment can often take an extended period (e.g., minimum 12 weeks).
2	Physiotherapists were better able to adapt and tailor rehabilitation programmes to suit individual needs, interests, or goals (e.g., considering number of exercises, dosage, available time, level of difficulty, value-based activities etc.).
3	Healthcare providers educated patients about their shoulder pathology, identifying the specific structure involved to explain the reason for pain and how it occurred. (e.g., using anatomy models and/or scan).
4	Healthcare providers and patients knew the benefits of exercise and its effectiveness as a first-line treatment strategy, providing patient testimonials in support of this.
5	Patients were provided with guidance on how to manage flare-ups/fluctuations in pain levels in response to normal daily activities or exercise.
6	Patients knew what to expect in terms of pain during exercise, and what level of exercise-induced pain is acceptable.
7	Patients were given time-based, meaningful, and realistic goals (e.g., relating to sleep, pain management, function)
8	Involving patients in the process of exercise prescription by providing them with a choice in what exercises they select and complete.
9	Patients were provided with information relating to their shoulder pain diagnosis, nature of pain, and factors that can cause pain.
10	Healthcare providers and patients had a better understanding of evidence-based treatment recommendations for shoulder pain, highlighting to patients the support for exercise therapy as first-line treatment rather than 'hands on' therapy, surgery, or injections (incl. which type of exercise and particular dosage is most evidence-based).
11	Patients were able to see early signs of progress in relation to pain and range of motion in response to treatments, such as exercise therapy.
12	Patients understood the benefits and importance of persevering with exercise therapy.
13	Healthcare providers and patients understood the research evidence comparing the effectiveness, expected long-term outcomes and the benefits and risks associated with different treatment options available.
14	Patients had opportunities to practice exercises with their Physiotherapist and get ongoing support and feedback throughout their treatment programme (e.g., virtually, or face-to-face).
15	Healthcare providers acknowledged and explored patient fears and concerns (e.g., regarding exercise), and provided regular reassurance in relation to treatment progress and prognosis
16	Patients were provided with clear and unambiguous information on what will improve shoulder pain and what will exacerbate symptoms (e.g., activity modification, healthy lifestyle etc.)
17	Healthcare providers and patients developed and maintained a strong therapeutic relationship, built on honesty and trust, where patients feel their healthcare provider has listened carefully to their whole story, their goals, and has addressed all their fears and concerns.
18	Healthcare providers took more time to educate patients regarding their condition, imaging findings and effective treatments, directing them towards reliable sources of information.
19	Patients understood the motivation, effort and commitment required for exercise therapy, and felt a sense of ownership and had the confidence to self-manage their rehabilitation (e.g., how to pace, regress and progress exercises)
20	All HCPs, in particular first-contact HCPs (e.g., GPs), promoted and set early patient expectations for first-line treatment options, while also reducing the frequency of unnecessary referral for imaging and surgical opinion.
21	Healthcare providers and patients understood that injection and surgery don't offer a 'quick-fix' solution.
22	Patients had regular follow-up contact with their healthcare providers throughout the rehabilitation journey, as well as supportive resources to help them to continue exercising (e.g., reminders, exercise tracker APPs, diaries, printed exercise sheets)
23	Healthcare providers and patients understood that imaging findings do not always correlate with pain.
24	Patients had a clear understanding of the relevance of radiology (if any) and what normal age-related changes would be expected.
25	Healthcare providers delivered a unified and consistent message in relation to the best management approach, with greater collaboration and agreement on how best to present evidence-based findings to patients.
26	Patients had access to high-quality supervised group-based exercise classes (e.g., specific shoulder classes, clinical Pilates)
27	All members of the healthcare team were considered equal.
28	The public were better informed about shoulder pain, the role of exercise, benefits of physical activity, why MRI and surgery are not the answer, and the importance of active involvement in the recovery process (e.g., via public/media campaigns)
29	There was better communication amongst HCPs and the patient in relation to discussing evidence-based treatments and outcomes, supporting shared decision-making and more informed treatment choices.
30	Evidence-based guidelines and information for HCPs and patients regarding managing shoulder pain were more easily accessible (e.g., national advice booklet, website, workshops, seminars etc.)
31	Healthcare providers and patients had an understanding of pain science, in particular, that pain does not always equal harm or indicate the presence of something sinister (e.g., pain during exercise)
32	Healthcare providers understood the need to refer for first-line treatment in a timely manner, and to provide patients with information about shoulder pain and rehabilitation for those on waiting lists to help reduce risk of chronicity and patient frustration.
33	Patients were better informed about how to manage pain, including the role of analgesia to relieve pain and support engagement with rehabilitation.

34	Healthcare providers and patients were provided with clear, concise understandable evidence-based information (e.g., infographics, videos, handouts)
35	Healthcare providers and patients understood that imaging is not necessary for the successful management of shoulder pain and is only appropriate for cases that do not resolve after first-line treatment, or if red flags are present.
36	Physiotherapists placed greater emphasis on patient education as part of rehabilitation, with clear understanding of rationale behind conservative management and proposed mechanisms underpinning these
37	Patients knew that shoulder pain is difficult to diagnose, and much pain can be classified as non-specific.
38	Patients were provided with more education relating to factors that can impact recovery potential and contribute to shoulder pain (e.g., stress, obesity, smoking, fear avoidance, comorbidities etc.)
39	Healthcare policy relating to shoulder pain was more aligned with current best-evidence, with improved financial support for patients to engage in rehabilitation irrespective of public or private healthcare.
40	Patients understood that shoulder pain is common and age-related changes are a normal part of life.
41	Healthcare providers and patients had an awareness of positive prognostic indicators such as high self-efficacy and the belief that evidence-based treatment will work.
42	There was more Information for healthcare providers to support research exploring the best management approach for shoulder pain, with more financial funding available to support these activities.
43	There were more resources available to improve public health literacy from a young age.
44	Healthcare providers and patients understood the natural history of atraumatic shoulder pain.
45	If a patient's first contact involves access to specialist or advanced-practice shoulder physiotherapists with the ability to directly access imaging, if required.

Supplementary Material 3: Preliminary categories circulated to Public & Patient Involvement (PPI) Advisory Panel

(green=very important, yellow=important, orange=somewhat important)

Categories	Priorities*	
	**Patient priorities**	**Healthcare Provider priorities**
**Treatment options & supporting evidence**	Treatment options & supporting evidence	Evidence-based treatment recommendations highlighting support for exercise therapy as first-line treatment rather than 'hands on' therapy, surgery, or injections.
	Surgical success rates, risks & that it is contraindicated	
	Benefits of exercise Vs pharmacological treatment	Research evidence comparing the effectiveness, long-term outcomes & risks/benefits of treatment
	Inclusion of manual therapy/dry needling	All HCPs promoted & set early patient expectations for first-line treatment options; reducing unnecessary imaging & surgical referral.
		Easily accessible information/evidence-based guidelines on shoulder pain management
		Understood need for timely referral for first-line treatment, and to provide information about shoulder pain & rehabilitation for those on surgical waiting lists.
		Clear, concise & understandable evidence-based information (e.g., infographics, videos, handouts)
		Benefits of exercise and its effectiveness as first-line treatment (testimonials to support)
		Understood natural history of atraumatic shoulder pain
		Providing clear rationale behind conservative management & proposed mechanisms underpinning these
**Treatment for specialist HCP**	Treated by specialist shoulder physiotherapist	All members of the healthcare team were considered equal.
		First contact involving access to specialist/advanced practice shoulder physiotherapist
**Expected progress & recovery timeline**	Exact healing process	Level of recovery to expect at various time intervals depending on treatment, setting expectations that improvement with conservative treatment can often take an extended period (e.g., minimum 12 weeks).
	Time to improvement & difficulty in predicting this	Time-based, meaningful & realistic goals (e.g., relating to sleep, pain management, function)
	Realistic timeframe for recovery & return to normal activities	Understood that injection & surgery don't offer a 'quick-fix' solution.
	Time off work required post-surgery	Able to see early signs of progress in relation to pain & range of motion in response to treatments (e.g. exercise therapy).
	Exercise not a quick fix but best for long-term symptoms relief	
	Before & after visual of improvements achieved with treatment	
**A specific diagnosis**	Specific diagnosis for pain & what the injury was	Information relating to diagnosis, nature of pain, and factors that can cause pain.
		Difficult to diagnose & often classified as non-specific.
	Visual of body to help understand pain and identify offending body part	Education on shoulder pathology, identifying the specific structure & reason for pain/how it occurred using anatomy models/scan.
	Clear on cause of pain	
**The role of imaging in treatment planning.**	Understood MRI information & medical terminology to enable discussion with HCP	Imaging is not necessary for successful management & is only appropriate for those non-responsive to first-line treatment/presence of red flags.
	Referral for imaging to know specific cause for pain & recommended treatment	Imaging findings do not always correlate with pain.
		Relevance of radiology (if any) & what normal age-related changes would be expected.

		Shoulder pain is common & age-related changes are a normal part of life.
**Unified & consist message in relation to diagnosis & best management pathway**	What HCP to be referred to for treatment & how to access	Unified & consistent message in relation to the best management approach, with greater collaboration & agreement on how best to present evidence-based findings to patients.
	Clear care pathway	
	Unified diagnosis/message	Clear & unambiguous information on what will improve/exacerbate symptoms (e.g., activity modification, healthy lifestyle etc.)
**Strong Therapeutic Relationship: Trust & reassurance**	Reassured by HCP likelihood of positive treatment response	Strong therapeutic relationship built on honesty and trust, with healthcare provider listening carefully to whole story, goals, and addressing all fears & concerns.
	Good rapport & trust in competence of HCP	Acknowledged & explored patient fears and concerns (e.g., regarding exercise), and provided reassurance in relation to treatment progress & prognosis.
	HCP less abrupt and rushed with time	More time to educate patients regarding their condition, imaging findings & effective treatments, directing them towards reliable information sources.
**Tailoring exercise therapy**	Meaningful & convenient exercises	Better able adapt & tailor rehabilitation programmes to suit individual needs, interests, or goals.
		Involvement in exercise prescription by providing choice in what exercises they select and complete.
		Access to high-quality supervised group-based exercise classes.
**Shared decision-making**	HCP opinion to guide what treatment to explore first	Better communication amongst HCPs/HCPs and patients in relation to discussing evidence-based treatments & outcomes, supporting shared decision-making & informed treatment choices.
	Collaborative decision on treatment path with HCP	
**Commitment & perseverance involved in exercise therapy**	Level of commitment required to engage in rehab programme	Understood the motivation, effort and commitment required for exercise therapy, felt a sense of ownership, & had confidence to self-manage their rehabilitation (e.g., how to pace, regress and progress exercises)
	Importance of following treatment advice & exercise consistency to prevent reoccurrence	Benefits & importance of persevering with exercise therapy.
**Follow-up and support throughout rehab journey**	Additional resources and support to complete exercises	Regular follow-up contact with healthcare provider & resources to support exercise adherence throughout rehabilitation journey (e.g., reminders, exercise tracker APPs, diaries, printed exercise sheets)
	Reviewed by HCP to monitor progress, help motivate & provide feedback	Opportunities to practise exercises with their Physiotherapist and get ongoing support & feedback.
**12. Risk factors for developing pain & their**	Activities to increase/decrease risk of developing pain	More education relating to factors that can impact recovery potential and contribute to shoulder pain (e.g., stress, obesity, smoking, fear avoidance, comorbidities etc.)
		Awareness of positive prognostic indicators such as high self-efficacy & the belief that evidence-based treatment will work.

**influence on recovery**		
**13. Pain Management**	How to prevent boom-and-bust cycle	Guidance on how to manage flare-ups/fluctuations in pain in response to daily activities/exercise.
		What to expect in terms of pain during exercise, & what level of exercise-induced pain is acceptable.
		Understanding of pain science, in particular, that pain does not always equal harm or indicate the presence of something sinister (e.g., pain during exercise)
		Pain management & role of analgesia to support engagement with rehabilitation.
**14. Public healthcare information, policy & research aligned with current best-evidence**	Knowledge of long surgical waiting list & need to pay privately	Public better informed about shoulder pain, role of exercise, benefits of physical activity, why MRI & surgery are not the answer, and the importance of active involvement in the recovery process (e.g., via public/media campaigns)
		There was more Information for healthcare providers to support research exploring the best management approach for shoulder pain, with more financial funding available to support these activities.
		Healthcare policy relating to shoulder pain was more aligned with current best-evidence, with improved financial support for patients to engage in rehabilitation irrespective of public or private healthcare.
		There were more resources available to improve public health literacy from a young age.

* **NOTE**: Educational priorities are colour-coded based on outcome of importance rating survey (i.e., green=very important, yellow=important, orange=somewhat important)

Supplementary Material 4: Preliminary concept map circulated to Public & Patient Involvement (PPI) Advisory Panel, displaying educational categories (green) identified to influence adherence with evidence-based treatment recommendations for musculoskeletal shoulder pain (yellow), and also the perceived influence directional relationship between categories (red lines), based on PPI advisory panel commentary and feedback.

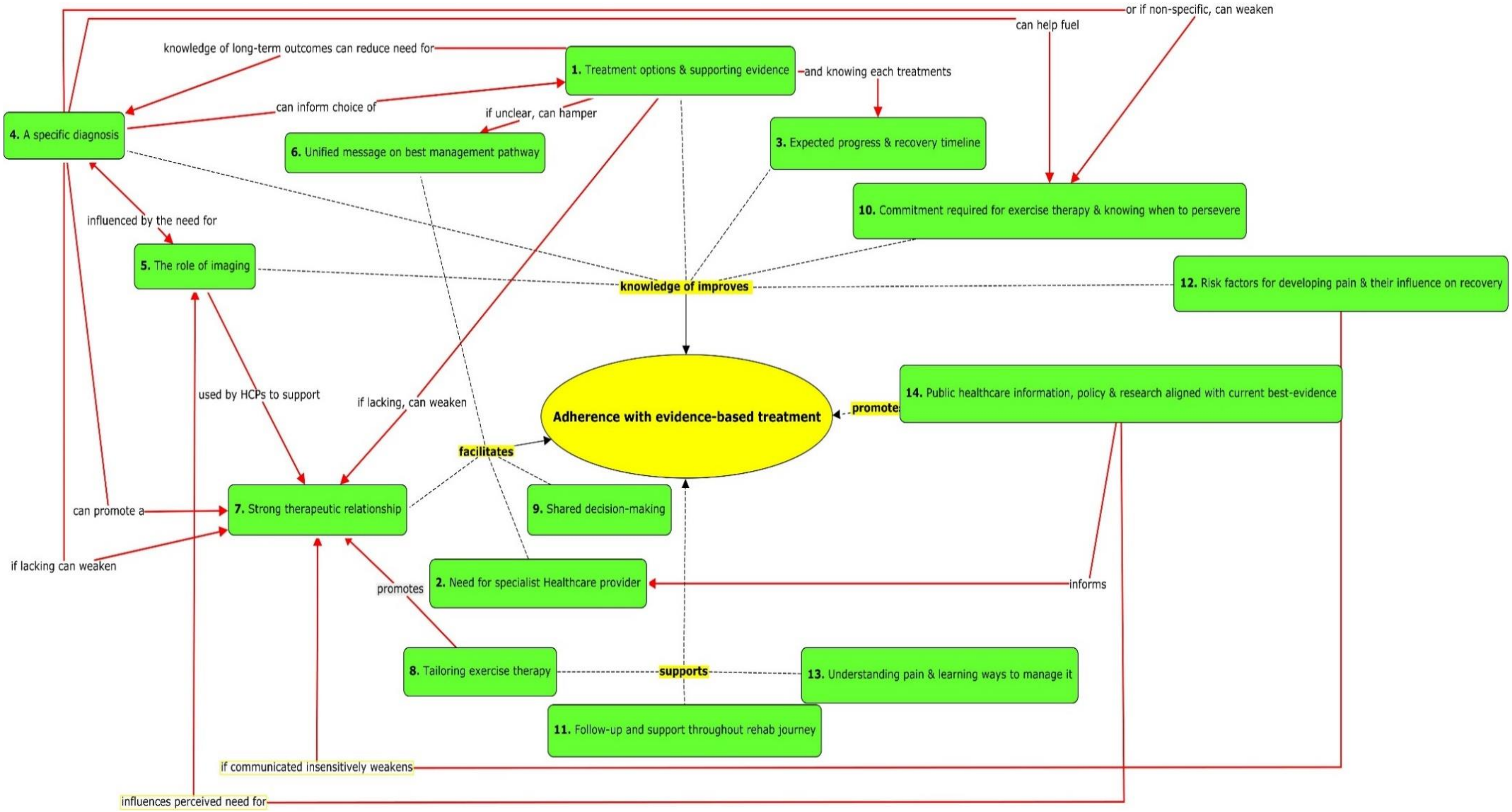

Supplementary Material 5. Revised Draft Concept Map illustrating Categories prioritised amongst Stakeholders as Important for Adherence to Evidence-based Recommendations, including their Perceived Directional Relationship.

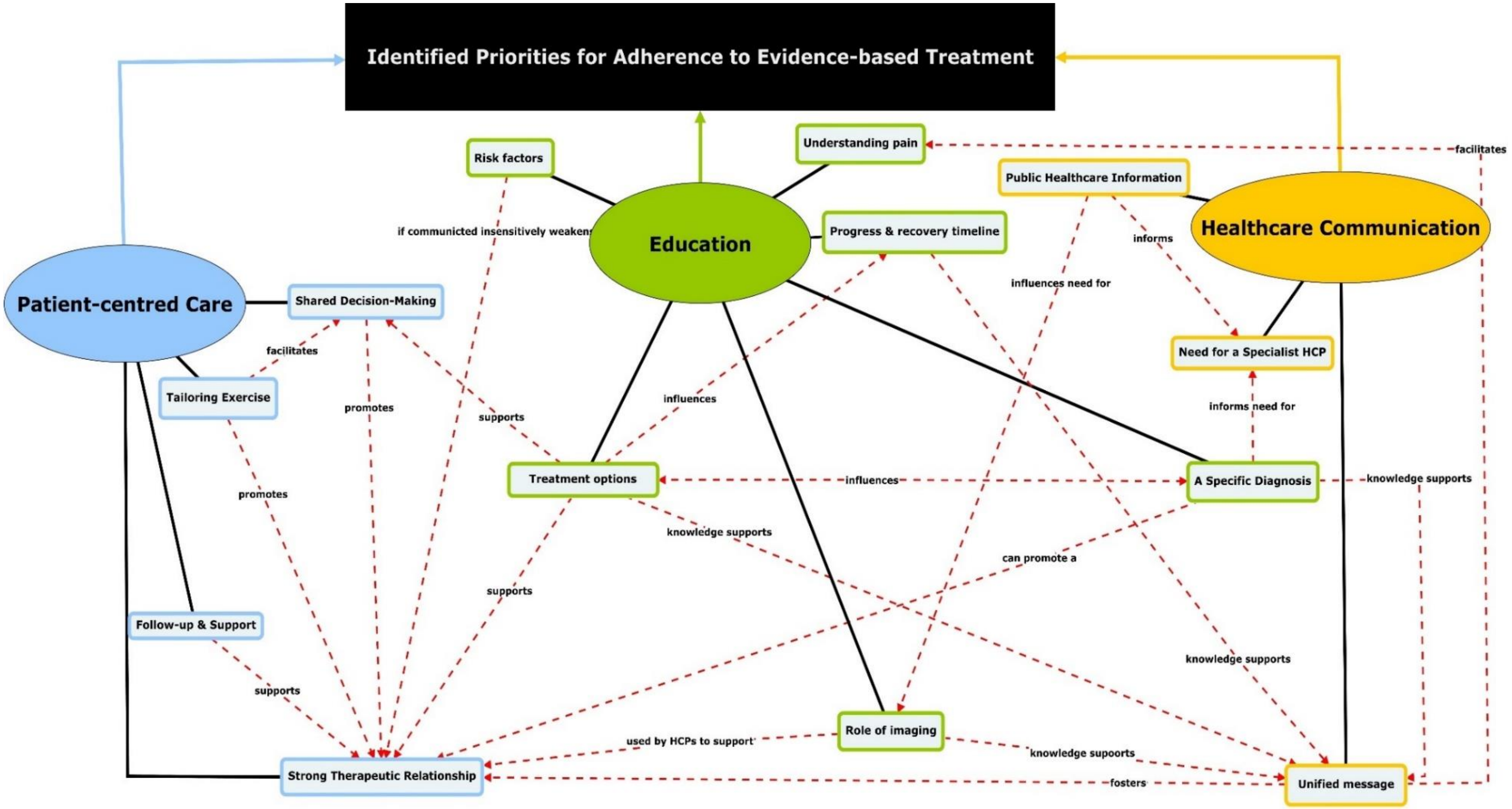

Supplement: 2023-0709_R1_Supplementary_Material_pzae176 [file 2023-0709_r1_supplementary_material_pzae176.pdf]
